# Two-Dimensional Infrared Spectroscopy Resolves the Vibrational Landscape in Donor–Bridge–Acceptor Complexes with Site-Specific Isotopic Labeling

**DOI:** 10.1021/acsphyschemau.4c00073

**Published:** 2024-10-29

**Authors:** James D. Shipp, Ricardo J. Fernández-Terán, Alexander J. Auty, Heather Carson, Andrew J. Sadler, Michael Towrie, Igor V. Sazanovich, Paul M. Donaldson, Anthony J. H. M. Meijer, Julia A. Weinstein

**Affiliations:** † Department of Chemistry, University of Sheffield. Sheffield S3 7HF, U.K.; ‡ Department of Physical Chemistry, University of Geneva, CH-1205 Geneva, Switzerland; § STFC Central Laser Facility, Research Complex at Harwell, Research Complex at Harwell, Harwell Science and Innovation Campus, Didcot, Oxford OX11 0QX, U.K.

**Keywords:** 2D-IR spectroscopy, donor–bridge–acceptor Pt(II) acetylides, vibrational relaxation, vibrational coupling, dynamic anharmonicity, vibrational coherences

## Abstract

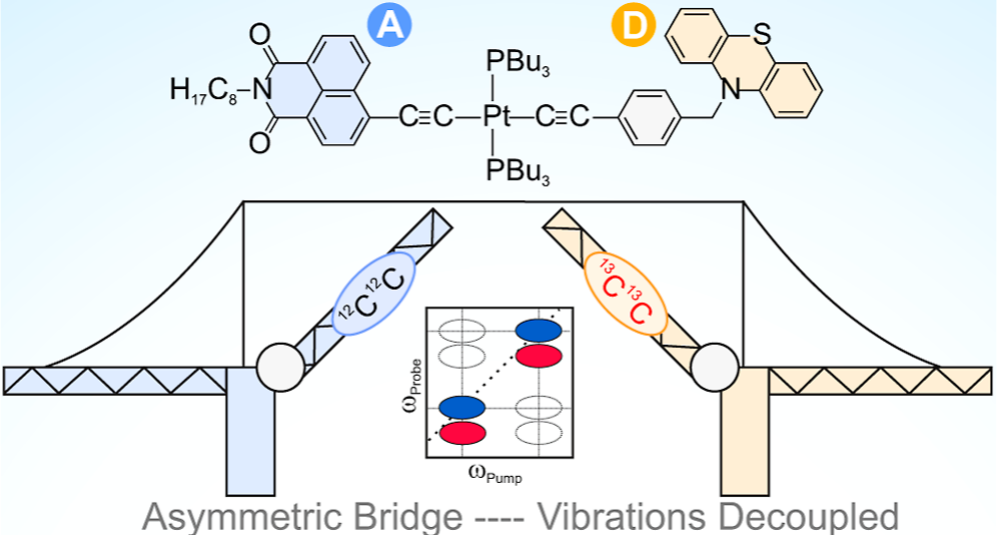

Donor–bridge–acceptor complexes (D–B–A) are important model systems for understanding of light-induced processes. Here, we apply two-color two-dimensional infrared (2D-IR) spectroscopy to D–B–A complexes with a *trans*-Pt(II) acetylide bridge (D–C≡C–Pt–C≡C–A) to uncover the mechanism of vibrational energy redistribution (IVR). Site-selective ^13^C isotopic labeling of the bridge is used to decouple the acetylide modes positioned on either side of the Pt-center. Decoupling of the D-acetylide- from the A-acetylide- enables site-specific investigation of vibrational energy transfer (VET) rates, dynamic anharmonicities, and spectral diffusion. Surprisingly, the asymmetrically labeled D–B–A still undergoes intramolecular IVR between acetylide groups even though they are decoupled and positioned across a heavy atom usually perceived as a “vibrational bottleneck”. Further, the rate of population transfer from the bridge to the acceptor was both site-specific and distance dependent. We show that vibrational excitation of the acetylide modes is transferred to ligand-centered modes on a subpicosecond time scale, followed by VET to solvent modes on the time scale of a few picoseconds. We also show that isotopic substitution does not affect the rate of spectral diffusion, indicating that changes in the vibrational dynamics are not a result of differences in local environment around the acetylides. Oscillations imprinted on the decay of the vibrationally excited acceptor-localized carbonyl modes show they enter a coherent superposition of states after excitation that dephases over 1–2 ps, and thus cannot be treated as independent in the 2D-IR spectra. These findings elucidate the vibrational landscape governing IR-mediated electron transfer and illustrate the power of isotopic labeling combined with multidimensional IR spectroscopy to disentangle vibrational energy propagation pathways in complex systems.

## 1 Introduction

Molecular systems capable of selectively populating charge-separated excited states as a result of photoinduced electron transfer (ET) are highly desirable for artificial photosynthesis and solar energy conversion.^1^ However, the efficiency of charge-separation is often limited by low quantum yields of ET, or unproductive back ET processes. An exciting prospect that could solve these problems is to direct the flow of ET in the excited state by mode-specific IR excitation. This form of controlling charge separation, which is chemically innocent and specific to particular vibrational modes, has been investigated theoretically,^2−7^ and demonstrated experimentally.^8−12^

Examples of systems where controllable ET has been demonstrated include hydrogen-bonded dimers of organic compounds,^4,13^ fullerenes bound to metallic surfaces,^14^ Re(I) tricarbonyl complexes,^15^ and Pt(II) bis-acetylide donor–bridge–acceptor (D–B–A) complexes.^9,11,16,17^

The experiments which demonstrate “vibrational control” of ET can be briefly summarized as follows. The D–B–A molecules are first electronically excited with a UV/vis pump pulse, leading to the population of an intermediate excited state that can decay over several pathways including formation of a charge-separated state. Once the intermediate state is populated, a second, mid-IR, pump pulse selectively excites a mode that is perceived to be coupled to the charge separation pathway, inducing changes in the rates of charge transfer^9,11,13,15−18^ and in some cases altering the branching ratio.^9,11,17^

**Figure 1:**
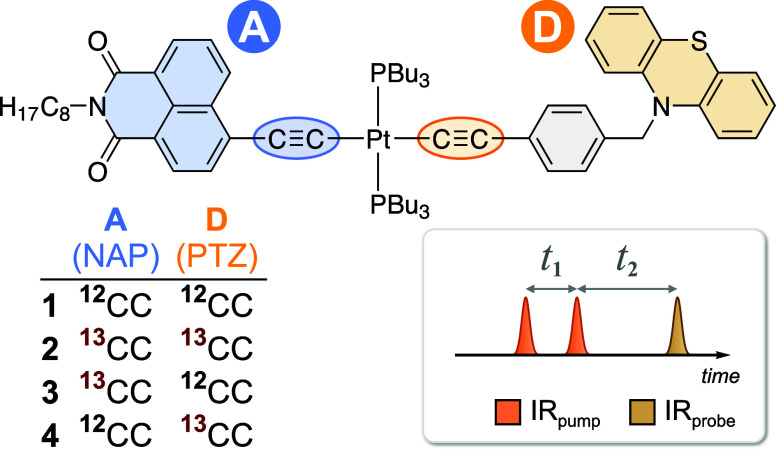
Structures of the studied complexes, illustrating the labeled acetylide groups on the donor (D, orange) and acceptor (A, blue) moieties. Inset: pulse sequence for time-domain 2D-IR, two IR-pump pulses separated by time delay *t*_1_ are followed by a probe pulse at time delay *t*_2_.

Despite the experimental demonstration of vibrational control, its underlying mechanism is not yet understood. Resolving intermode anharmonic coupling, intramolecular vibrational energy redistribution (IVR) and mode (de)localization could be crucial in determining which modes contribute to the reaction coordinate that drives charge separation, and hence identifying which modes could act as a handle to modify the rates and yields of charge separation. Here, we report on vibrational coupling and vibrational energy transfer (VET) in a series of *trans*-Pt(II) D–B–A complexes with a phenothiazine-based electron donor (PTZ) and a naphthalene monoimide acceptor (NAP) unit, each joined to the central {Pt(PBu_3_)_2_} moiety by an acetylide bridge for which vibrational control has been demonstrated (Figure 1).^9^

We use a combination of ultrafast time-domain two-dimensional infrared spectroscopy (2D-IR) and density functional theory (DFT) calculations to understand the vibrational dynamics of this important class of D–B–A complexes. 2D-IR spectroscopy is a powerful spectroscopic technique, which provides detailed information on the vibrational lifetimes, anharmonicities, energy transfer pathways, quantum coherences, intermolecular bonding interactions, anisotropic responses, and solvation dynamics.^19−28^ These spectroscopic properties offer insight into how excited vibrational states relax through intramolecular energy transfer and intermolecular interactions with the local environment.

2D-IR spectroscopy uses two ultrafast pump pulses to populate vibrational excited states (Figure 1, inset), followed by a probe pulse that detects the evolution of these states over the pump–probe delay time (*t*_2_). In this case,^29^ we use a pulse shaper to generate the two required pump pulses, which are separated by the coherence time delay (*t*_1_). The *t*_1_ delay is scanned to obtain a vibrational free induction decay at each probe frequency, which is then Fourier transformed to produce a 2D contour map. The resulting 2D-IR spectra represent correlation maps of ω_pump_ against ω_probe_ taken at each *t*_2_. In this work, we use 2D-IR spectroscopy to understand how the vibrational modes of the *trans*-Pt(II) D–B–A complexes relax after IR excitation.

Our previous studies have shown that vibrational excitation of the acetylide stretching vibration of these complexes in the branching charge-transfer excited state fully deactivates the ET process,^9^ with the excited-state population being redirected to a NAP-localized triplet or to the ground state. In a related *cis*-Pt(II) complex bearing two structurally identical PTZ–C≡C electron donor ligands and a bidentate bipyridyl acceptor, ^13^C isotopic substitution of only one acetylide group was found to decouple the two acetylide stretching vibrations, enabling selective suppression of ET along the pathway with the vibrationally excited acetylide and demonstrating the possibility of pathway-specific ET control in forked molecular systems.^17^

Here, we use isotopic substitution to gain further insight into vibrational coupling in the ground state of the linear D–B–A complexes. The ^13^C labeling offers a tool to vibrationally decouple the acetylide group on the donor and the acceptor side of the Pt center. Developing an understanding of the vibrational couplings in the electronic ground state provides key information on how isotopic labeling of the acetylide groups influences vibrational coupling and the energy flow through the D–B–A system that is crucial for resolving the more complex processes involving VET in the electronic excited states.

The parent complex, *trans*-[(NAP–C≡C)Pt^II^(PBu_3_)_2_(C≡C–PTZ)] (**1**) was ^13^C-labeled either at both acetylides (**2**), only on the NAP–C≡C (**3**), or only on the PTZ–C≡C moiety (**4**). Isotopic substitution of a single acetylide provides a vibrational spectroscopic handle for selective mid-IR excitation of either the electron acceptor or donor side, by decoupling (and thus localizing) the vibrational modes. The doubly labeled complex (**2**) provided a reference point to investigate the isotope effect of ^13^C labeling on the dynamics of the acetylide groups in the absence of vibrational decoupling. The resulting detailed picture of the vibrational landscape provided by 2D-IR spectroscopy demonstrates how energy transfer across the bridge is hindered by isotopic substitution due to localization of the vibrational excited states on either side of the metal center. We evidence this decoupling effect through comparison of energy transfer rates between the bridge and the ligands and dynamic changes in anharmonicity during vibrational relaxation, supported by anharmonic frequency calculations.

## 2 Results and Discussion

### 2.1 FTIR Spectroscopy and Normal Mode Analysis

**Figure 2:**
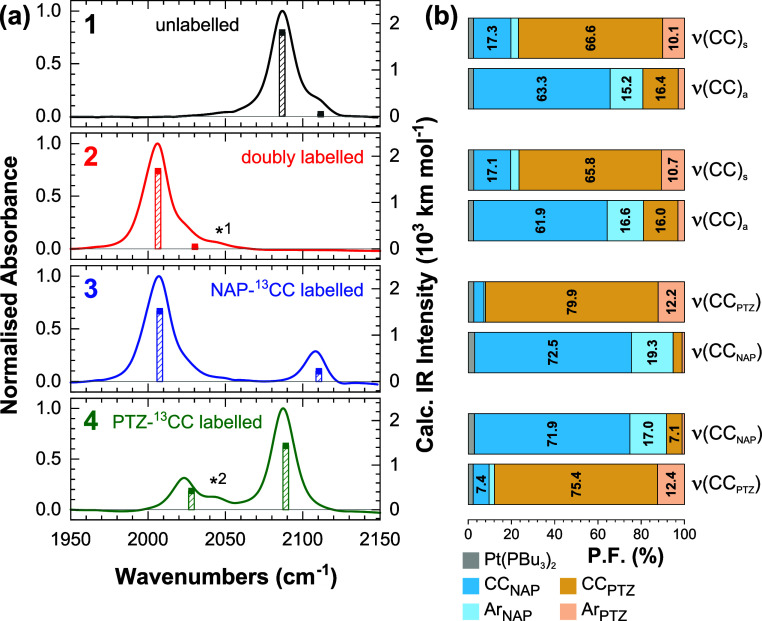
(a) Normalized ground state FTIR spectra of complexes **1**–**4** in CH_2_Cl_2_ (solid lines), overlaid with calculated harmonic frequencies (scaled by 0.935) and intensities (bars) in the ν(C≡C) region. (b) Participation factors (P.F.) of the corresponding molecular fragments in the C≡C stretching normal modes (P.F. < 5% not shown). Additional features marked as *1 and *2 are discussed in the text.

The experimental FTIR spectra and calculated vibrational transitions of **1**–**4** in the 1950–2150 cm^–1^ region, which corresponds to the acetylide group vibrations [ν(CC)], are shown in Figure 2A. In **1**, antisymmetric [ν(CC)_a_] and symmetric [ν(CC)_s_] group vibrations of the acetylide bridges were observed at 2087 and 2107 cm^–1^, respectively. In the doubly ^13^C-labeled complex (**2**), both modes are downshifted by ca. 80 cm^–1^, consistent with the changes in reduced mass (Supporting Information Section S3.1).

To quantify the spatial localization of the acetylide vibrations, the contribution of each C≡C group to the acetylide normal modes (Figure 2B) was calculated as P.F. The P.F. is defined as the sum of the vibrational displacement vectors (in mass-weighted coordinates) for the atom(s) of interest, normalized to the total displacement of all atoms in the associated normal mode.^30^

In **1**, the antisymmetric acetylide stretching group vibration is primarily localized on the CC_NAP_ moiety, while the symmetric group vibration is largely localized on the CC_PTZ_ group, consistent with the previous DFT^31^ and mode projection analyses.^5^ The P.F. for **1** and **2** are very similar, showing that full isotopic substitution does not affect the character of the acetylide group vibrations, only their frequencies. In all complexes, the aromatic groups (Ar_NAP_, Ar_PTZ_) contribute up to 20% in the acetylide vibrations.

The FTIR spectra of the singly labeled complexes **3** and **4** are significantly different to those of **1** and **2**. The FTIR spectra of **1** and **2** exhibit a large peak associated with ν(CC)_a_ and a small shoulder associated with ν(CC)_s_, while in **3** and **4** we observe two well-resolved bands that are separated by 101 and 64 cm^–1^, respectively. These two bands now correspond to localized CC_NAP_ and CC_PTZ_ stretching vibrations. When the contribution of the corresponding Ar_*x*_ moieties is included, the ν(CC) modes localize up to 93% on the associated ligand. Whether these modes are coupled is not clear from linear spectroscopy, thus demonstrating the need for multidimensional spectroscopy.

Regardless of isotopic substitution, the absorption band associated with ν(CC_NAP_) is significantly more intense than the corresponding ν(CC_PTZ_) band. This effect is attributed to a greater vibrational transition dipole moment caused by conjugation of the acetylide π-bond with the electron-deficient NAP group. The C≡C bond on the PTZ side is less polarized, as it is conjugated with a relatively more electron-rich phenyl group. In the fully labeled or unlabeled “symmetric” complexes (**1** and **2**), the ν(CC)_s_ mode has a much lower oscillator strength, as expected from the local symmetry. These intensity patterns are also reproduced by the harmonic frequency calculations.

When the CC_PTZ_ group was ^13^C labeled (i.e., in **2** and **4**), the ν(^13^CC_PTZ_) associated absorption band splits into a doublet (features marked as *1 and *2 in Figure 2A, respectively). A similar effect was observed in *cis*-(diimine)Pt^II^ bis-acetylides with identical donor groups, where it was attributed to an accidental Fermi resonance due to mixing with a Ph–CH_2_–PTZ mode.^17^ Given that the donor ligand is the same for both *cis* and *trans* complexes, we attribute the observed splitting of the IR-absorption band in **2** and **4** to the same effect.

**Figure 3:**
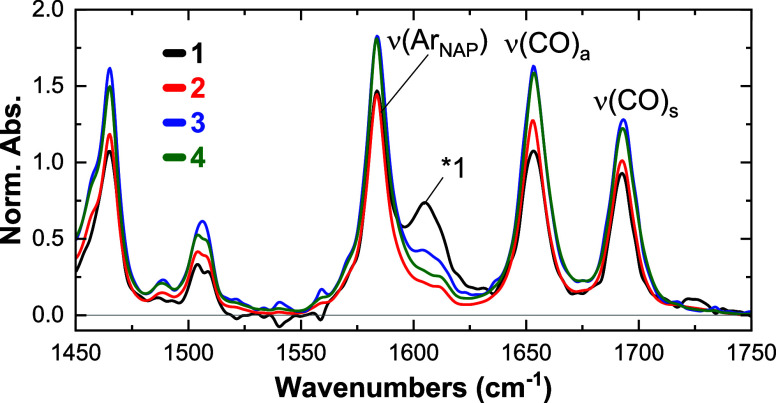
FTIR spectra of complexes **1**–**4** in CH_2_Cl_2_ in the 1450–1750 cm^–1^ region. *1 is attributed to residual water content in the samples. Spectra are normalized relative to the acetylide region.

The vibrational modes of the NAP and Ph–CH_2_–PTZ groups in the 1450–1750 cm^–1^ region are not significantly altered by acetylide isotopic substitution, Figure 3. In all complexes, three prominent vibrational absorption bands of the NAP ligand were found at 1583, 1653, and 1692 cm^–1^, corresponding respectively to the NAP C=C aromatic stretching [ν(Ar_NAP_)], antisymmetric C=O [ν(CO)_a_], and symmetric C=O stretching [ν(CO)_s_] modes. The calculated harmonic IR spectra (Supporting Information, Figure S3) allow us to assign the lower intensity bands in the 1450–1750 cm^–1^ region to aromatic stretching/bending modes of the NAP and PTZ ligands. The P.F. of the two C=O groups show full delocalization of the ν(CO)_a_ and ν(CO)_s_ group vibrations between the two carbonyls (with some contribution from the naphthalene ring), in contrast with the localized P.F. of the acetylide modes.

### 2.2 Vibrational Couplings and Relaxation in the Acetylide Region

Analysis of the FTIR spectra has established that single isotopic labeling of the D–B–A complex separated the asymmetric stretching mode observed in **1** and **2** into two individual modes that are primarily associated with the donor or acceptor acetylide groups. With this information, we apply 2D-IR spectroscopy to examine the vibrational dynamics of the D–B–A compounds and evaluate the overall impact of isotopic labeling as well as the effect of asymmetric labeling on the vibrational coupling in **1**–**4**.

**Figure 4:**
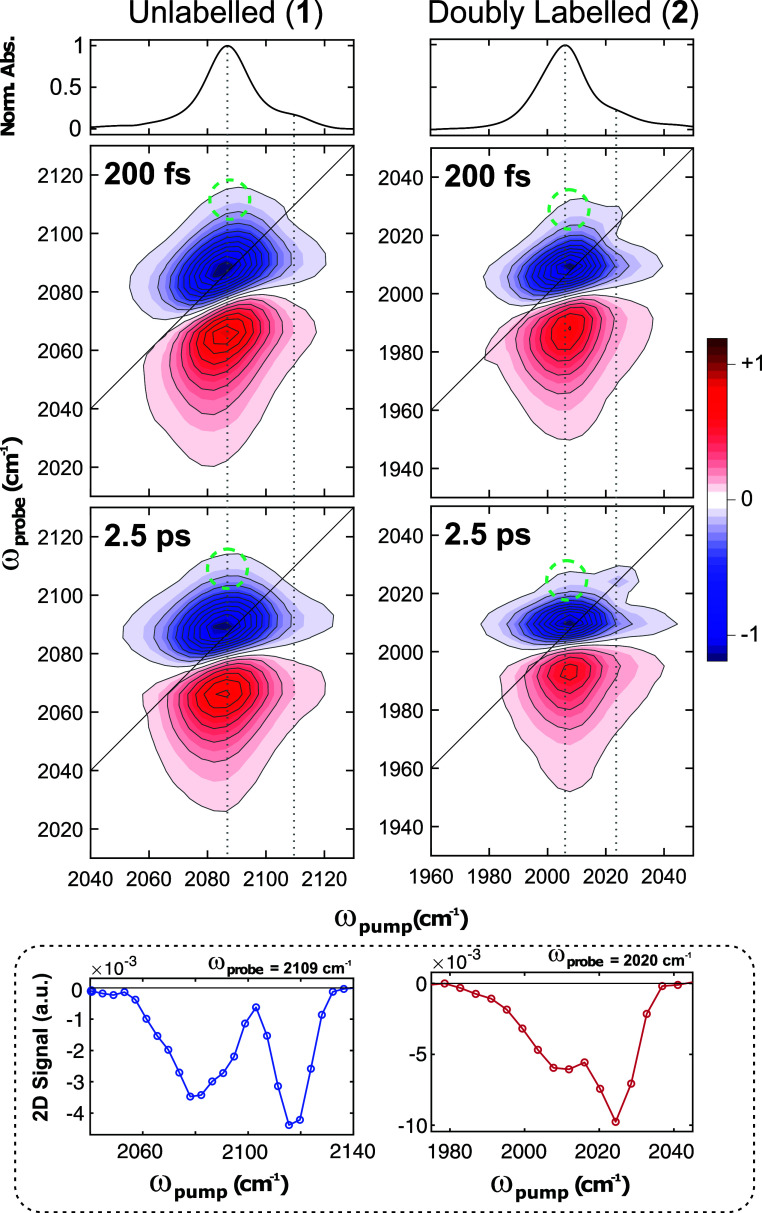
FTIR (top row) and 2D-IR spectra of **1** (left column) and **2** (right column) in the acetylide stretching region. Vertical lines guide the eye to the main absorption bands, green circles denote a cross peak between the two modes. Cross sections of the 2D-IR spectra along the excitation axis (*t*_2_ = 2.5 ps) are shown in the inset that more clearly highlight the cross-peaks. Additional cross sections are shown in the Supporting Information (Figures S12 and S13).

The 2D-IR spectra of **1** and **2** primarily consist of a peak pair arising from excitation of the ν(CC)_a_ mode, with small shoulder peaks at higher frequency that correspond to the ν(CC)_s_ vibration, Figure 4. 2D-IR spectra taken in the range of lower ω_probe_ frequencies are included in the Supporting Information (Figures S4 and S5). In the 2D-IR spectra shown here, the negative peaks (blue) correspond to ground state bleaches (GSB) of the ν_0–1_ transitions, while the positive (red) peaks correspond to excited state absorption bands (ESA) of the ν_1–2_ transitions.

In the symmetrically labeled complexes (**1** and **2**), the acetylide group vibrations [ν(CC)_a_ & ν(CC)_s_] are strongly mechanically coupled due to their delocalization over both C≡C groups, as shown by the calculated P.F. This coupling is demonstrated by the presence of cross-peaks between these modes, which were observed in the 2D-IR spectra at the first available time delay of 200 fs. The signals at shorter time delays are convolved with the instrument response function and such perturbed free induction decay signals and are not analyzed here.^32,33^ Restricted-space anharmonic DFT calculations were used to estimate the diagonal and off-diagonal anharmonicities (Supporting Information, Figure S1), yielding Δ_*ii*_^calc^ ≈ 15–16 cm^–1^ and Δ_ij_^calc^ ≈ 4 cm^–1^, from which we can estimate an initial cross-peak intensity of ca. 16% [as the initial cross-peak amplitudes scale with ].^19,34^ Due to the difference in intensities of the two transitions, these cross-peaks are evidenced only as off-diagonal weak shoulders in the GSB signal of the spectra (Figure 4, green circles). Cross sections of the 2D-IR spectra taken through the cross-peak maxima that more clearly show the cross-peaks are given in the Supporting Information (Figures S12 and S13). The experimental anharmonicities of the diagonal signals for both **1** and **2** were Δ_*ii*_ ≈ 19–20 cm^–1^, in good agreement with calculations. The off-diagonal anharmonicity could not be determined experimentally due to the strong overlap and difference in intensities between the two modes.

The kinetic traces of the diagonal ν(CC)_a_ peaks show a biexponential decay, with time constants of 0.91 ps (95%) and 3.7 ps (5%) for **1**; and values of 0.88 ps (91%) and 7.7 ps (9%) for **2**. Toward lower frequencies, rapid growth of cross-peaks corresponding to the NAP ligand ν(CO)_s_ and ν(CO)_a_ modes is observed on the 1–2 ps time scale (Supporting Information, Table S2 and Figure S14). The formation of cross-peaks during the *t*_2_ delay period is typically attributed to population transfer, where vibrational energy is transferred from one mode to another during IVR.^35−37^ Therefore, the initial relaxation step is assigned to population transfer from the initially excited acetylide modes to ligand-centered modes. The 2D-IR spectra of the ligand mode cross-peaks are shown in the Supporting Information (Figures S4 and S5). The ν(CO)_s_, ν(CO)_a_, and ν(NAP_Ar_) cross-peaks have very similar lineshapes to their diagonal counterparts, which further supports assignment of the acetylide to ligand IVR process as population transfer (Figure S11). The rate of population transfer is slightly faster in **2**, due to the smaller energy separation between the acetylide and ligand vibrational levels, which is also evidenced by a faster growth rate for the ν(CO) cross-peaks (Supporting Information, Table S2). The second decay component of the acetylide vibrational excited states takes place on the picosecond time scale and corresponds to VET to solvent bath modes (ν_*i*_ < *k*_B_*T* ≈ 200 cm^–1^ at 298 K), as is typically observed for transition metal complexes in solution.^27,34,38−40^

**Figure 5:**
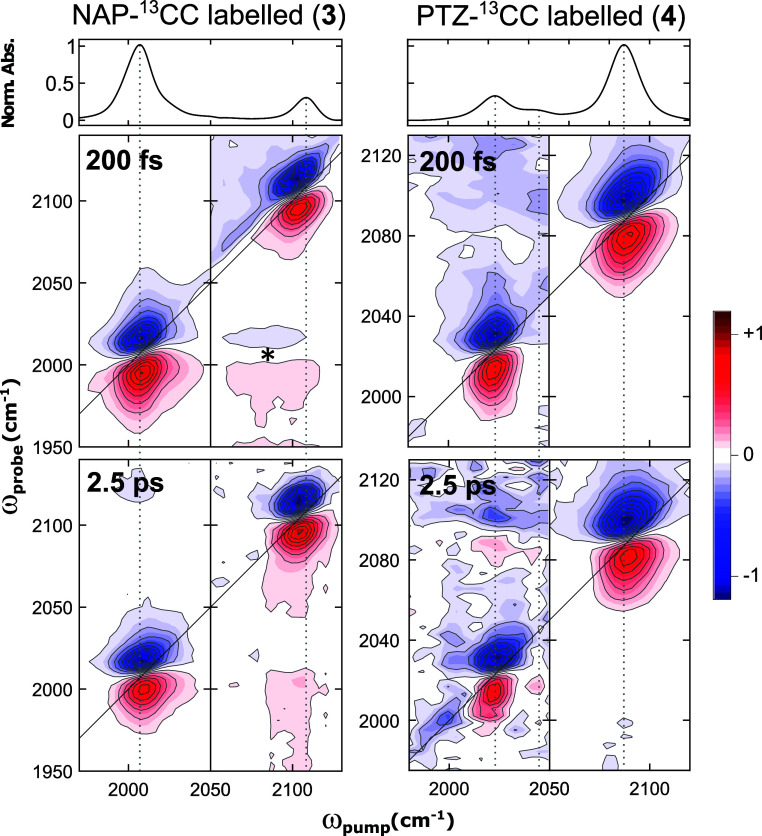
FTIR (top row) and 2D-IR spectra of **3** (left column) and **4** (right column) in the acetylide stretching region. Each 2D-IR data set was recorded by centring the pump (fwhm ≈80 cm^–1^) at the corresponding mode. The data are normalized to the maximum diagonal intensity of each spectrum. Vertical lines guide the eye to the main absorption bands. The feature labeled with a * is an artifact that likely results from aliasing of the diagonal peak pair at ω_pump_ = 2010 cm^–1^.

The 2D-IR spectra of the singly labeled complexes (**3**–**4**), where the two vibrational modes become localized, are significantly different to those of **1**–**2**. Due to their larger frequency separation and the limited pump bandwidth of our setup (ca. 80 cm^–1^ fwhm)^29^ we measured two data sets for each complex, centering the pump spectrum around the corresponding ν(CC) band (Figure 5). In both complexes, two well resolved peak pairs were observed, that correspond to either the ν(CC_NAP_) or ν(CC_PTZ_) modes. The initial (*t*_2_ = 200 fs) cross-peak amplitudes were negligible, showing that no direct anharmonic coupling exists between the localized acetylide modes—in agreement with our calculations (Δ_*ij*_ ≈ 0). The negligible cross-peak intensity at small *t*_2_ delays is also in agreement with the calculated P.F. for the acetylide modes of **3** and **4**, demonstrating near-complete localization of the acetylide vibrations to either the donor or acceptor ligands in the asymmetrically labeled complexes.

**Scheme 1:**
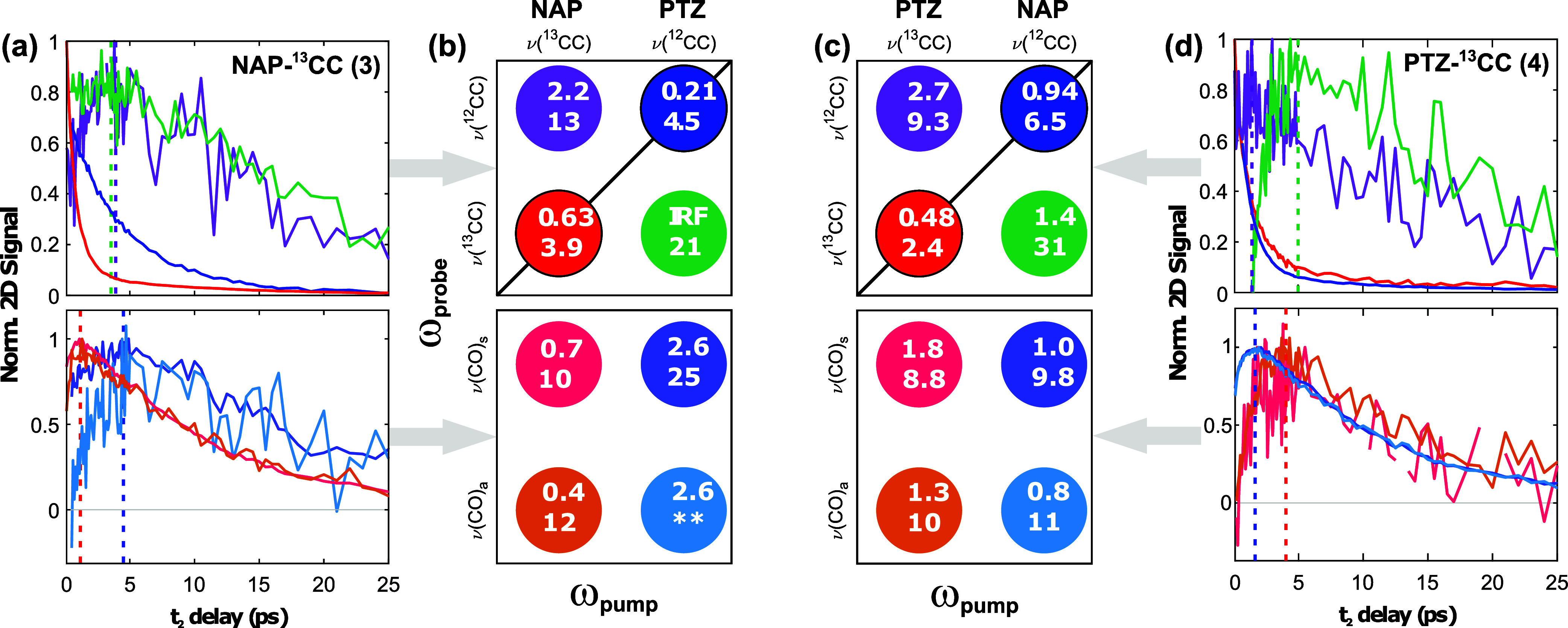
Normalised Kinetic Traces for the Diagonal and Cross Peaks Obtained from the 2D-IR Spectra Following Acetylide Excitation of Complex **3** (a) and **4** (d) [Probe: Top Row: Acetylide Region; Bottom Row: ν(CO) Region] Vertical dashed lines illustrate the approximate position of the maximum intensity for the corresponding cross peaks. (b,c) Colour legend and schematic summary of the fitted time constants (in ps) for **3** and **4**, Respectively. ↑/↓ Indicate the rise/decay Lifetimes, respectively. The lifetimes are obtained from biexponential fitting of the kinetic traces (Supporting Information, Section S6). The exponential amplitudes and time constants are provided in the Supporting Information, Table S2. The ** lifetime is associated with a large fit error, making it unreliable. Cross sections and additional 2D-IR spectra are provided in the Supporting Information that show the peaks associated with the kinetic traces of the acetylide cross peaks more clearly (Figures S9 and S10).

As in **1** and **2**, the diagonal peaks of **3** and **4** decay biexponentially. Scheme 1 shows the kinetic traces of the diagonal and off-diagonal peaks of interest for **3**–**4** (both in the acetylide and C=O stretching regions), with the associated time constants; the fit parameters are tabulated in the Supporting Information, Section S6.

As above, we attribute the first component of the decay of the diagonal signal to population transfer into ligand-centered modes, and the second to vibrational relaxation/VET to the solvent bath. The 2D-IR spectra obtained in the lower frequency probe region are shown in the Supporting Information (Figures S6 and S8).

Cross-peaks between the ν(CC_NAP_) and ν(CC_PTZ_) acetylide modes form on the ps time scale, reach their maximum intensities around 1–4 ps, and decay on the 10–20 ps time scale (Scheme 1, top panels). The formation of these cross-peaks is a result of IVR between the acetylide vibrational modes, where the energy required for uphill energy transfer is provided by the bath of low-frequency solvent modes. However, the low intensity of the cross-peaks indicates that this is a minor IVR pathway. As expected,^41,42^ the downhill cross peaks grow faster than the uphill ones, while their decay rates are similar. Contour maps, which are cropped to more clearly show these low intensity cross-peaks, are provided in the Supporting Information, Figure S9.

### 2.3 Vibrational Couplings and Relaxation to Ligand-Centred Modes

To assess the rates of VET between the acetylide bridge and the NAP and PTZ ligands, we utilize two different probe regions, which are centered at either 2050 or 1650 cm^–1^, enabling simultaneous measurement of energy transfer from the initially excited high frequency acetylide modes to the lower frequency ligand modes.

The calculated off-diagonal anharmonicities between acetylide and ligand-centered modes were negligible (Δ_*ij*_ ≈ 0) regardless of isotopic substitution, suggesting that the ligand-centered vibrational manifolds are not anharmonically coupled to the acetylide stretching manifold.

As population transfer from the vibrationally excited acetylide modes to ligand-centered modes takes place, we observe the growth of cross peaks at ω_probe_ frequencies of ν(CO)_s_, ν(CO)_a_, and ν(Ar_NAP_) modes on the 1–4 ps time scale for all complexes. Importantly, the kinetics of the ν(CC) → ν(CO) cross-peaks do not depend on the isotopic labeling but rather on the identity of the acetylide mode being excited. After excitation of the ν(CC_NAP_) modes, the ν(CO) cross peaks reach their maximum amplitude at ca. 1 ps, while they take somewhat longer (ca. 5 ps) to completely grow in when the corresponding ν(CC_PTZ_)—centered modes are excited instead. This can be explained by the conjugation between the C≡C and C=O moieties in the NAP ligand, and their closer location in space compared to the PTZ-associated moiety.

### 2.4 Dynamic Anharmonicities

In addition to the cross-peak dynamics, another indication of population transfer comes from the time-dependent evolution of the diagonal anharmonicities of the ν(CC) peaks. We utilize these dynamic changes in the diagonal anharmonicity to demonstrate the decoupling effect of isotopic substitution on energy transfer between the NAP and PTZ ligands, as discussed in the following.

**Figure 6:**
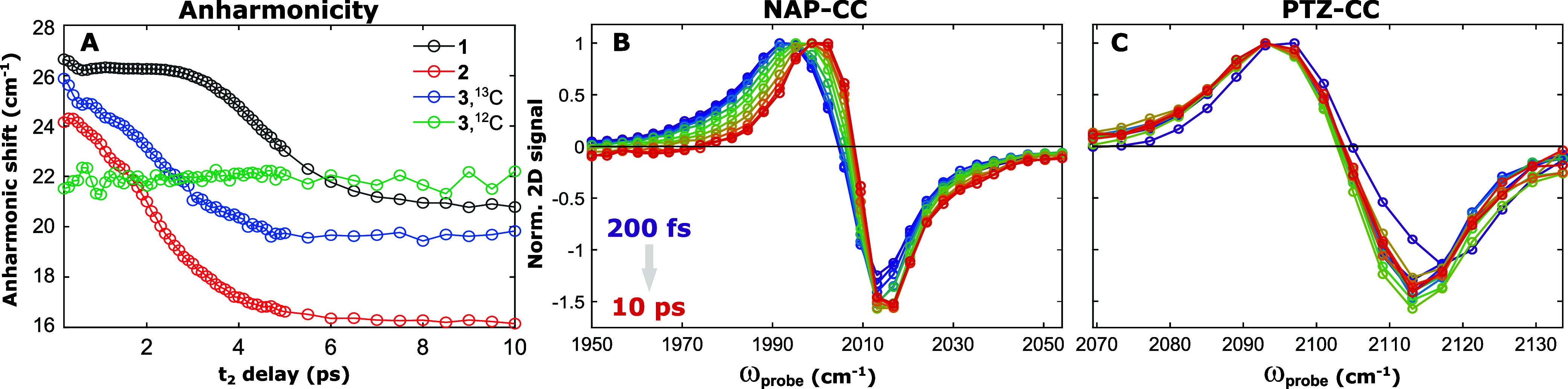
(a) *t*_2_-Dependent anharmonic shifts obtained for **1**–**3** by fitting of two 2D-Gaussian peaks to each 2D-IR spectrum. (b,c) Normalized cross sections of the 2D-IR spectra along the probe axis, intersecting the pump frequency maximum of the NAP-CC (b) or PTZ-CC (c) peak pair of complex **3** (data for **1**, **2**, and **4** are shown in the Supporting Information, Figure S25), demonstrating the dynamic changes in peak position with increasing *t*_2_ (purple → red).

In **1** and **2**, the apparent anharmonicity of the diagonal ν(CC)_a_ peak pair decreases with increasing *t*_2_ time (Figure 6A). The center positions of the ESA and GSB bands were obtained by fitting of 2D Gaussian peaks to the peak pair at each *t*_2_ time. The maximum shifts relative to those at *t*_2_ = 200 fs are in the order of 9 cm^–1^, and take place with time constants of 1.6–1.8 ps. Similar dynamics were observed in **3** and **4**. The ESA of the ν(^12^CC_NAP_)-centered modes shift by ca. 9 cm^–1^ over approximately 10 ps. In contrast, no shift was observed for the ν(CC_PTZ_)-centered modes (Figure 6B,C). This CC_NAP_ specific behavior was independent of isotopic labeling, and is consistent with that observed in the symmetric complexes (**1**–**2**), where the main peak pair corresponds to the antisymmetric stretching mode—primarily localized on the CC_NAP_ group.

Similar changes in the apparent anharmonic shift have been attributed previously to rapid population transfer to lower frequency modes.^10,43−46^ In a similar fashion, for **1**–**4** we ascribe these dynamics to population transfer from the initially excited CC_NAP_—localized mode to the NAP ligand modes [ν(CO)_s_, ν(CO)_a_, ν(NAP_Ar_)]. The shift of CC_NAP_ is not observed following CC_PTZ_ excitation due to decoupling of the two ligands by isotopic substitution.

**Figure 7:**
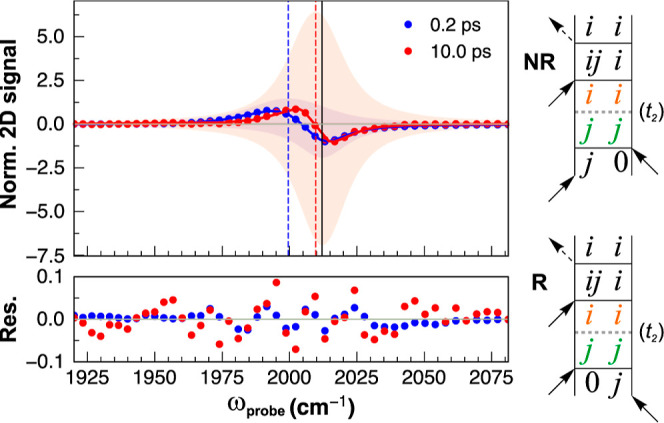
Left: normalized probe cross sections of the 2D-IR spectra taken at early (0.2 ps) and late (10 ps) population delays for ω_pump_ = ν(^13^CC_NAP_) in **3**, with fitted Voigt profiles for the GSB/ESA contributions. Vertical bars show the position of the fitted GSB (black), and early/late ESA (blue/red, respectively). Right: double-sided Feynman diagrams (rephasing and non-rephasing) that contribute to the observed ESA signal after population transfer, especially relevant at late *t*_2_ delays.

The ESA shift results from synchronous decay of the initial ν_1–2_(CC) ESA and grow-in of a blue-shifted ESA that corresponds to the response of the ν(CC_NAP_) mode when the ligand modes are vibrationally excited—analogous to the ν(CC) cross-peak formed following ν(CO) excitation (Supporting Information, Figure S7). As the growth and decay processes are simultaneous, a continuous shift of the ESA to higher frequencies is observed over time. The frequency of the GSB does not change during this process, and the small observed shift of the GSB is thought to be a result of increased peak cancellation of the positive and negative signal contributions at smaller anharmonic shifts. Indeed, fitting of the early (0.2 ps) and late (10 ps) *t*_2_ delay probe cross sections of the 2D-IR spectra of complex **3** (as a representative example) with Voigt profiles (Figure 7) reveals the change in apparent anharmonicity (Further details on the Voigt fitting procedure are provided in the Supporting Information, Section S8).

To illustrate how the three ultrafast pulses lead to the formation of different peaks in the 2D-IR spectra, we utilize double-sided third order Feynman diagrams, which show how different population or coherent states are generated as well as the frequencies they will be detected at.^19,47^ The rephasing and non-rephasing Feynman diagrams that contribute to the observed signals after population transfer during *t*_2_ (i.e., |*j*⟩⟨*j*| → |*i*⟩⟨*i*|) are presented in Figure 7. The absorptive 2D-IR signal we measure here is the sum of the rephasing (R) and non-rephasing (NR) contributions, hence both the R and NR pathways will contribute to the observed shift in the ESA bands. In this context, *j* refers to the acetylide modes and *i* to the ν(CO) modes. The diagrams in Figure 7 show how the two pump pulses (arrows) create population states (e.g., |*j*⟩⟨*j*|). After population transfer in *t*_2_ to form |*i*⟩⟨*i*|, the probe pulse then creates a coherence during the *t*_3_ detection time (|*ij*⟩⟨*i*|). The resulting ESA is therefore detected at the same frequency as if we initially excited mode *i*, and detected at mode *j*. Considering this, the origin of the time-dependent shift of the diagonal ESA lies in the difference between the diagonal (Δ_*jj*_) and off-diagonal (Δ_*ij*_) anharmonicities: the former contributing at early times and the latter becoming more prominent after population transfer has taken place. Similar observations have been made in the past by Garrett-Roe and co-workers, who detected an unexpected additional ESA with a smaller anharmonic shift for CO_2_ in viscous media, which they attributed to a “hot” ground state.^48,49^ In our case, the coupling (Δ_*ij*_^exp^) between the acetylide and carbonyl modes in **3** and **4** is very small (ca. 1 cm^–1^) but not exactly zero, leading to a large difference (Δ_*jj*_ – Δ_*ij*_). We were able to observe this weak effect due to the very high signal-to-noise ratio of our spectrometer.^29^

It should also be noted that the decrease in the apparent anharmonic shift also results in a larger cancellation of the positive and negative contributions to the signal, leading to an overall decrease in intensity (the traces in Figure 7 are normalized). This shows how the time scale associated with population transfer is intertwined with the decay kinetics of the diagonal peak pairs.

The fact that the ESA shifts are only observed for NAP-localized vibrational modes implies the modes on either side of the Pt(II) center cannot equilibrate within the vibrational lifetime. If this were the case, one would observe similar shifts in the transients of the PTZ-localized modes. An alternative explanation would be that the difference in diagonal and off-diagonal anharmonicities of ν(CC_PTZ_) is very small (Δ_*jj*_ ≈ Δ_*ij*_), which would imply very strong coupling between the modes and the presence of cross peaks at very early *t*_2_ delays, contrary to our observations. Our results are consistent with some of the previous accounts of transition metal centers,^17,45,50^ or other heavy atoms^51^ acting as “vibrational bottlenecks” that inhibit VET between decoupled modes. In **3**–**4** the Pt(II) center acts as a similar bottleneck, preventing IVR between the two acetylide groups. However, in **1** and **2**, the strong anharmonic coupling between the two acetylide group vibrations allows for VET across the Pt(II) center.

### 2.5 Spectral Diffusion

2D-IR spectroscopy is also able to follow ultrafast fluctuations in the vibrational frequency of a mode. These fluctuations arise from the dynamic nature of the solvent–solute interactions and from the motion of the nuclei involved in the vibrational mode of interest, and manifest in the observed line shapes of both linear (FTIR) and nonlinear (2D-IR) spectra. The time scale of these fluctuations can be estimated from the decay of the (normalized) frequency fluctuation correlation function (FFCF). There are several methods that can be used to obtain the FFCF from the 2D-IR spectra, for example, the nodal line slope,^52^ center line slope (CLS),^53^ inverse CLS,^26,54,55^ inhomogeneity index,^56^ 2D-Gaussian fitting,^57^ as well as Kubo model fitting techniques.^58^ Here, we use the CLS method to approximate the normalized FFCF (Supporting Information, eq S12), providing an estimate of the spectral diffusion time scale for the vibrational modes (Supporting Information, Section S7).

The spectral diffusion dynamics of the acetylide peak pairs were studied to determine how the decoupled acetylide vibrational modes interact with their local environment. At early *t*_2_ times, the acetylide diagonal peak pairs in **1**–**4** are slightly elongated along the diagonal axis (Figures 4 and 5, top rows). As *t*_2_ increases, spectral diffusion takes place and the bands become rounder (Figures 4 and 5, bottom rows). This change results from the excited molecules resampling their available vibrational frequency space during solvent and molecular motion.

**Figure 8:**
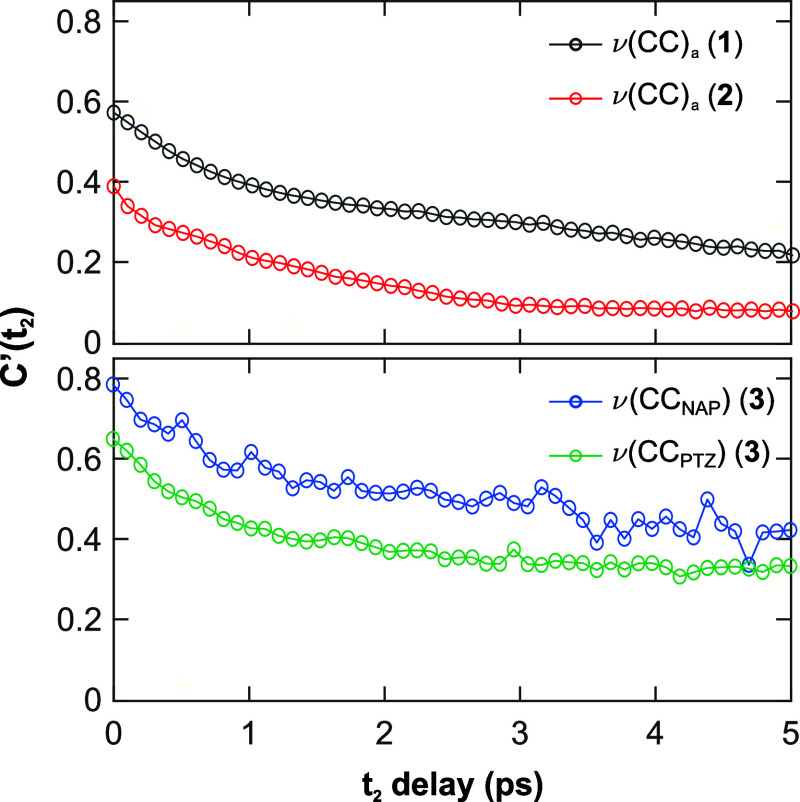
Normalized FFCFs for the diagonal acetylide peak pairs of complexes **1**, **2**, and **3** (as a representative example). These FFCFs are reproduced in the Supporting Information with error bars included. Data for **4** are shown in the Supporting Information, Figure S19.

The CLS method used here approximates the “normalized FFCF”, but the correlation curves start at values below 1.0 at *t*_2_ = 200 fs. This shows that some ultrafast spectral diffusion takes place during the IRF-convolved *t*_2_ delays (0–200 fs). The spectral diffusion time constants of the acetylide modes were obtained by monoexponential fitting of the FFCFs. The time constants were similar for **1**–**4**, ranging between 0.93–2.2 ps (Figure 8; Table S3). This similarity in the rate of spectral diffusion is clearer if the FFCFs are normalized to their initial values (Supporting Information, Figure S21). The similar FFCF kinetics show that the local environment around the NAP-CC and PTZ-CC groups fluctuate on similar time scales. Thus, the observed changes in the vibrational relaxation kinetics upon asymmetric labeling (**3** and **4**) likely do not result from differences in the local environment dynamics around the decoupled acetylide groups.

**Figure 9:**
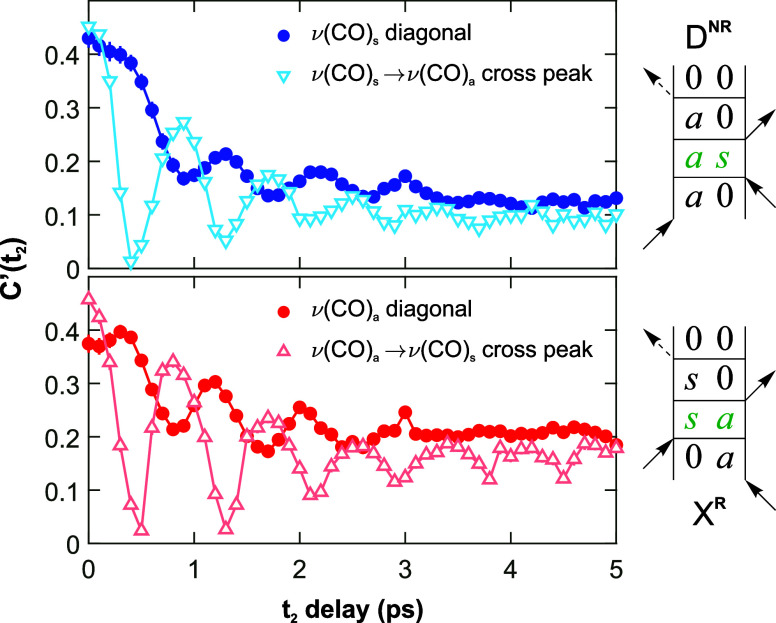
Normalized FFCFs for the diagonal and cross-peaks of the ν(CO)_s_ and ν(CO)_a_ modes of complex **3** (as a representative example). Also shown are example double-sided Feynman diagrams for the diagonal (**D**^NR^) and cross-peak (**X**^R^) GSBs that lead to coherent oscillations during the population time (highlighted in green). The FFCFs are reproduced with the fit error bars included in Figure S22 in the Supporting Information.

The FFCF decays of the symmetric [ν(CO)_s_] and antisymmetric [ν(CO)_a_] carbonyl stretching modes are imprinted with an oscillatory component, whose period corresponds to ca. 40 cm^–1^ (Figure 9), matching the frequency separation of the two vibrations (Supporting Information, Section S7.3). The same oscillations were also observed in the kinetic traces of the corresponding peak pairs (Supporting Information, Section S9). We attribute these oscillations to a ν(CO)_s_/ν(CO)_a_ coherence present during *t*_2_. Similar observations have been made previously for [Cp′Mn(CO)_3_], where the imprinted oscillation was attributed to a coherence between vibrational modes.^56^ The oscillating coherence shows that the two ν(CO) vibrations form a quantum superposition state during the *t*_2_ delays, and cannot be treated as independent modes after broadband IR excitation of both transitions.^22,24,42^

The coherent oscillation in the FFCFs can be explained with the third order Feynman diagrams in Figure 9, where the quantum superposition state is represented by |*a*⟩ ⟨*s*| or |*s*⟩ ⟨*a*|. The coherence causes oscillations in the rephasing and non-rephasing contributions to the absorptive 2D-IR spectra, where only diagonal peaks oscillate in the non-rephasing spectra (**D**^NR^), and only cross-peaks oscillate in the rephasing spectra (**X**^R^). Thus, there is a superimposed oscillation in the kinetics of all four peak pairs in the absorptive (A) 2D-IR spectrum of the NAP CO-modes (*S*_A_ = *S*_R_ + *S*_NR_). The oscillating signal becomes imprinted onto the FFCF because it is related to the relative amplitude of the rephasing and non-rephasing spectra, as quantified by the inhomogeneity index (Supporting Information, eqs S13 and S14).^56^ The FFCFs of the two diagonal peak pairs oscillate in phase with one another, and out of phase with the cross-peaks. We attribute this to the fact that, on the corresponding Feynamn diagrams, the *t*_2_ coherences appear as |*a*⟩ ⟨*s*| and |*s*⟩ ⟨*a*|, respectively, thus having a π relative phase shift.

To obtain the spectral diffusion rates of the carbonyl modes of the NAP ligand, an exponentially damped cosine function (Supporting Information, eq S15) was used to account for oscillations present in the FFCF.^59^ The resulting time constants for the carbonyl modes of **1**–**4** were found to be approximately 0.7 ps (Supporting Information, Table S4). The FFCFs obtained in both the ν(C≡C) and ν(C=O) regions of the 2D-IR spectra have a long-lived offset after the exponential decay, with an amplitude of 0.1–0.5. This is attributed to long-lived inhomogeneities that decay beyond the 5 ps time scale shown in Figures 8 and 9.^27^

## 3 Conclusions

The mechanism of vibrational relaxation in Pt^II^*trans*-acetylide D–B–A complexes, where the bridge is a –C≡C–Pt–C≡C– group, has been elucidated with two-color time-domain 2D-IR spectroscopy. Here, we employ site-selective ^13^C isotopic substitution in order to resolve the contributions of individual acetylide groups connecting the donor or the acceptor to the Pt center during vibrational energy propagation in the ground state. We show that the vibrationally excited acetylides relax by ultrafast IVR (<1 ps) to lower frequency ligand modes, followed by VET to the solvent bath (3.7 ps). Symmetric isotopic substitution of both –C≡C– of the bridge did not alter the vibrational relaxation mechanism, but decreased the rate of VET two-fold. However, labeling only one C≡C group leads to decoupling of the two acetylide ligands, which permits individual perturbation of an acetylide group on either D or A side of the molecule. Surprisingly, in the asymmetrically labeled D–B–A, IVR still takes place between the acetylide groups even though they are decoupled (^13^C vs ^12^C, Δν = 80 cm^–1^), and positioned across a heavy atom “vibrational bottleneck”. Further, the rate of population transfer from the bridge to the C=O groups on the acceptor was site-specific, with much faster IVR rates from the nearby ν(CC_NAP_) group compared to that from ν(CC_PTZ_) across the Pt-center.

The fluctuation rate of the local environment of the acetylide modes is not affected by decoupling, as evident from the similarity of the frequency fluctuation correlation functions obtained from the time dependence of the 2D-IR lineshapes. The similar line shape changes of the two acetylide groups indicates that the local environment dynamics on either side of the Pt-center are similar, and do not lead to the observed differences in the acetylide VET kinetics upon decoupling. The two strongly coupled C=O group vibrations of the acceptor moiety, separated by 40 cm^–1^, enter a coherent state after being simultaneously excited by a broadband IR pump, as evidenced by an imprinted oscillation in the vibrational decay kinetics and FFCF kinetics of these modes. The formation of a coherent state between the C=O modes shows that they cannot be treated independently in the 2D-IR spectra.

Altogether, the analysis of the ground state 2D-IR spectra presented here shows how changes to the molecular vibrations of a D–B–A complex, introduced by isotopic labeling of the bridging group, can significantly alter the vibrational relaxation dynamics and intermode couplings. This approach allows for selective IR excitation of the vibrationally decoupled donor or acceptor ligands, and presents a useful tool in the interpretation and understanding of the vibrational kinetics obtained by transient 2D-IR spectroscopy of the electronically excited states in this class of D–B–A molecules.

## 4 Materials and Methods

The synthesis of complexes **1**–**4** follows a previously reported protocol,^31^ and full details are provided in the Supporting Information (Section S1). FTIR spectra were recorded on a PerkinElmer Spectrum One spectrometer at 2 cm^–1^ resolution in a demountable liquid cell equipped with two 2 mm CaF_2_ windows, in dichloromethane solution. 2D-IR spectra were recorded at the LIFEtime facility CLF-STFC, Rutherford Appleton Laboratory.^29^ Full details of the 2D-IR method, as well as the experimental parameters used for data collection are provided in the Supporting Information, Section S10. DFT calculations were performed at the University of Sheffield following a previously developed protocol,^11^ where geometry optimization and harmonic frequency calculations were performed in Gaussian 09 revision D.01.^60^ Anharmonic frequency calculations were performed on the Yggdrasil HPC cluster of the University of Geneva in Gaussian 16 revision A.03^61^ (full details are provided in the Supporting Information, section S2).

## Data Availability

The FTIR and 2D-IR data supporting the findings from this study are available upon request.

## Supporting Information Available

Additional figures and tables, discussions and full experimental details including synthesis, characterization, computational details, and details about ultrafast and steady-state spectroscopic methods (PDF)

## References

[ref1] Wang Z.; Hu Y.; Zhang S.; Sun Y. Artificial photosynthesis systems for solar energy conversion and storage: platforms and their realities. Chem. Soc. Rev. 2022, 51, 6704–6737. 10.1039/D1CS01008E. 35815740

[ref2] Skourtis S. S.; Waldeck D. H.; Beratan D. N. Inelastic electron tunneling erases coupling-pathway interferences. J. Phys. Chem. B 2004, 108, 15511–15518. 10.1021/jp0485340.

[ref3] Xiao D.; Skourtis S. S.; Rubtsov I. V.; Beratan D. N. Turning charge transfer on and off in a molecular interferometer with vibronic pathways. Nano Lett. 2009, 9, 1818–1823. 10.1021/nl8037695. 19435376

[ref4] Ma Z.; Lin Z.; Lawrence C. M.; Rubtsov I. V.; Antoniou P.; Skourtis S. S.; Zhang P.; Beratan D. N. How can infra-red excitation both accelerate and slow charge transfer in the same molecule?. Chem. Sci. 2018, 9, 6395–6405. 10.1039/C8SC00092A. 30310568 PMC6115705

[ref5] Yang X.; Keane T.; Delor M.; Meijer A. J.; Weinstein J.; Bittner E. R. Identifying electron transfer coordinates in donor-bridge-acceptor systems using mode projection analysis. Nat. Commun. 2017, 8, 14554 10.1038/ncomms14554. 28233775 PMC5333094

[ref6] Valianti S.; Skourtis S. S. Vibrational control of molecular electron transfer reactions. Mol. Phys. 2019, 117, 2618–2631. 10.1080/00268976.2018.1504997.

[ref7] Mandal S.; Daniel C. Ultrafast Excited-State Nonadiabatic Dynamics in Pt(II) Donor–Bridge–Acceptor Assemblies: A Quantum Approach for Optical Control. J. Phys. Chem. A 2024, 128, 3126–3136. 10.1021/acs.jpca.4c00646. 38619836

[ref8] Bakulin A. A.; Rao A.; Pavelyev V. G.; van Loosdrecht P. H. M.; Pshenichnikov M. S.; Niedzialek D.; Cornil J.; Beljonne D.; Friend R. H. The Role of Driving Energy and Delocalized States for Charge Separation in Organic Semiconductors. Science 2012, 335, 1340–1344. 10.1126/science.1217745. 22362882

[ref9] Delor M.; Scattergood P. A.; Sazanovich I. V.; Parker A. W.; Greetham G. M.; Meijer A. J. H. M.; Towrie M.; Weinstein J. A. Toward control of electron transfer in donor-acceptor molecules by bond-specific infrared excitation. Science 2014, 346, 1492–1495. 10.1126/science.1259995. 25525241

[ref10] Bakulin A. A.; Selig O.; Bakker H. J.; Rezus Y. L.; Müller C.; Glaser T.; Lovrincic R.; Sun Z.; Chen Z.; Walsh A.; Frost J. M.; Jansen T. L. Real-Time Observation of Organic Cation Reorientation in Methylammonium Lead Iodide Perovskites. J. Phys. Chem. Lett. 2015, 6, 3663–3669. 10.1021/acs.jpclett.5b01555. 26722739

[ref11] Delor M.; Keane T.; Scattergood P. A.; Sazanovich I. V.; Greetham G. M.; Towrie M.; Meijer A. J.; Weinstein J. A. On the mechanism of vibrational control of light-induced charge transfer in donor-bridge-acceptor assemblies. Nat. Chem. 2015, 7, 689–695. 10.1038/nchem.2327. 26291939

[ref12] Gallop N. P.; Maslennikov D. R.; Mondal N.; Goetz K. P.; Dai Z.; Schankler A. M.; Sung W.; Nihonyanagi S.; Tahara T.; Bodnarchuk M. I.; Kovalenko M. V.; Vaynzof Y.; Rappe A. M.; Bakulin A. A. Ultrafast vibrational control of organohalide perovskite optoelectronic devices using vibrationally promoted electronic resonance. Nat. Mater. 2024, 23, 88–94. 10.1038/s41563-023-01723-w. 37985838 PMC10769873

[ref13] Lin Z.; Lawrence C. M.; Xiao D.; Kireev V. V.; Skourtis S. S.; Sessler J. L.; Beratan D. N.; Rubtsov I. V. Modulating unimolecular charge transfer by exciting bridge vibrations. J. Am. Chem. Soc. 2009, 131, 18060–18062. 10.1021/ja907041t. 19928957

[ref14] Pasupathy A. N.; Park J.; Chang C.; Soldatov A. V.; Lebedkin S.; Bialczak R. C.; Grose J. E.; Donev L. A.; Sethna J. P.; Ralph D. C.; McEuen P. L. Vibration-assisted electron tunneling in C_140_ transistors. Nano Lett. 2005, 5, 203–207. 10.1021/nl048619c. 15794596

[ref15] Yue Y.; Grusenmeyer T.; Ma Z.; Zhang P.; Schmehl R. H.; Beratan D. N.; Rubtsov I. V. Electron transfer rate modulation in a compact Re(I) donor–acceptor complex. Dalton Trans. 2015, 44, 8609–8616. 10.1039/C4DT02145B. 25600849

[ref16] Scattergood P. A.; Delor M.; Sazanovich I. V.; Towrie M.; Weinstein J. A. Ultrafast charge transfer dynamics in supramolecular Pt(II) donor-bridge-acceptor assemblies: The effect of vibronic coupling. Faraday Discuss. 2015, 185, 69–86. 10.1039/C5FD00103J. 26428717

[ref17] Delor M.; Archer S. A.; Keane T.; Meijer A. J. H. M.; Sazanovich I. V.; Greetham G. M.; Towrie M.; Weinstein J. A. Directing the path of light-induced electron transfer at a molecular fork using vibrational excitation. Nat. Chem. 2017, 9, 1099–1104. 10.1038/nchem.2793. 29064501

[ref18] Mendis K. C.; Li X.; Valdiviezo J.; Banziger S. D.; Zhang P.; Ren T.; Beratan D. N.; Rubtsov I. V. Electron transfer rate modulation with mid-IR in butadiyne-bridged donor-bridge-acceptor compounds. Phys. Chem. Chem. Phys. 2024, 26, 1819–1828. 10.1039/D3CP03175F. 38168814

[ref19] Hamm P.; Zanni M. Concepts and Methods of 2D Infrared Spectroscopy; Cambridge University Press: Cambridge, 2011.

[ref20] Hochstrasser R. M. Two-dimensional IR-spectroscopy: Polarization anisotropy effects. Chem. Phys. 2001, 266, 273–284. 10.1016/S0301-0104(01)00232-4.

[ref21] Golonzka O.; Khalil M.; Demirdöven N.; Tokmakoff A. Vibrational anharmonicities revealed by coherent two-dimensional infrared spectroscopy. Phys. Rev. Lett. 2001, 86, 2154–2157. 10.1103/PhysRevLett.86.2154. 11289878

[ref22] Khalil M.; Demirdöven N.; Tokmakoff A. Vibrational coherence transfer characterized with Fourier-transform 2D IR spectroscopy. J. Chem. Phys. 2004, 121, 362 10.1063/1.1756870. 15260555

[ref23] Naraharisetty S. R. G.; Kasyanenko V. M.; Rubtsov I. V. Bond connectivity measured via relaxation-assisted two-dimensional infrared spectroscopy. J. Chem. Phys. 2008, 128, 104502 10.1063/1.2842071. 18345901

[ref24] Nee M. J.; Baiz C. R.; Anna J. M.; McCanne R.; Kubarych K. J. Multilevel vibrational coherence transfer and wavepacket dynamics probed with multidimensional IR spectroscopy. J. Chem. Phys. 2008, 129, 084503 10.1063/1.2969900. 19044831

[ref25] Anna J. M.; Ross M. R.; Kubarych K. J. Dissecting enthalpic and entropic barriers to ultrafast equilibrium isomerization of a flexible molecule using 2DIR chemical exchange spectroscopy. J. Phys. Chem. A 2009, 113, 6544–6547. 10.1021/jp903112c. 19514782

[ref26] Fenn E. E.; Fayer M. D. Extracting 2D IR frequency-frequency correlation functions from two component systems. J. Chem. Phys. 2011, 135, 074502 10.1063/1.3625278. 21861571

[ref27] Kiefer L. M.; Kubarych K. J. Two-dimensional infrared spectroscopy of coordination complexes: From solvent dynamics to photocatalysis. Coord. Chem. Rev. 2018, 372, 153–178. 10.1016/j.ccr.2018.05.006.

[ref28] Askelson P. G.; Meloni S. L.; Hoffnagle A. M.; Anna J. M. Resolving the Impact of Hydrogen Bonding on the Phylloquinone Cofactor through Two-Dimensional Infrared Spectroscopy. J. Phys. Chem. B 2022, 126, 10120–10135. 10.1021/acs.jpcb.2c03556. 36444999

[ref29] Donaldson P. M.; Greetham G. M.; Shaw D. J.; Parker A. W.; Towrie M. A 100 kHz Pulse Shaping 2D-IR Spectrometer Based on Dual Yb:KGW Amplifiers. J. Phys. Chem. A 2018, 122, 780–787. 10.1021/acs.jpca.7b10259. 29250947

[ref30] Fernández-Terán R.; Ruf J.; Hamm P. Vibrational Couplings in Hydridocarbonyl Complexes: A 2D-IR Perspective. Inorg. Chem. 2020, 59, 7721–7726. 10.1021/acs.inorgchem.0c00750. 32410448

[ref31] Scattergood P. A.; Delor M.; Sazanovich I. V.; Bouganov O. V.; Tikhomirov S. A.; Stasheuski A. S.; Parker A. W.; Greetham G. M.; Towrie M.; Davies E. S.; Meijer A. J.; Weinstein J. A. Electron transfer dynamics and excited state branching in a charge-transfer platinum(II) donor-bridge-acceptor assembly. Dalton Trans. 2014, 43, 17677–17693. 10.1039/C4DT01682C. 25361227

[ref32] Hamm P. Coherent effects in femtosecond infrared spectroscopy. Chem. Phys. 1995, 200, 415–429. 10.1016/0301-0104(95)00262-6.

[ref33] Yan S.; Seidel M. T.; Tan H. S. Perturbed free induction decay in ultrafast mid-IR pump-probe spectroscopy. Chem. Phys. Lett. 2011, 517, 36–40. 10.1016/j.cplett.2011.10.013.

[ref34] Fernández-Terán R.; Hamm P. A Closer Look Into the Distance Dependence of Vibrational Energy Transfer on Surfaces Using 2D IR Spectroscopy. J. Chem. Phys. 2020, 153, 154706 10.1063/5.0025787. 33092354

[ref35] Kasyanenko V. M.; Lin Z.; Rubtsov G. I.; Donahue J. P.; Rubtsov I. V. Energy transport via coordination bonds. J. Chem. Phys. 2009, 131, 154508 10.1063/1.3246862. 20568873

[ref36] Rubtsov I. V. Relaxation-assisted two-dimensional infrared (ra 2dir) method: accessing distances over 10 å and measuring bond connectivity patterns. Acc. Chem. Res. 2009, 42, 1385–1394. 10.1021/ar900008p. 19462972

[ref37] Marroux H. J.; Orr-Ewing A. J. Distinguishing population and coherence transfer pathways in a metal dicarbonyl complex using pulse-shaped two-dimensional infrared spectroscopy. J. Phys. Chem. B 2016, 120, 4125–4130. 10.1021/acs.jpcb.6b02979. 27070852

[ref38] Kiefer L. M.; Kubarych K. J. Solvent-dependent dynamics of a series of rhenium photoactivated catalysts measured with Ultrafast 2DIR. J. Phys. Chem. A 2015, 119, 959–965. 10.1021/jp511686p. 25607849

[ref39] Kiefer L. M.; King J. T.; Kubarych K. J. Dynamics of rhenium photocatalysts revealed through ultrafast multidimensional spectroscopy. Acc. Chem. Res. 2015, 48, 1123–1130. 10.1021/ar500402r. 25839193

[ref40] Delor M.; Sazanovich I. V.; Towrie M.; Spall S. J.; Keane T.; Blake A. J.; Wilson C.; Meijer A. J.; Weinstein J. A. Dynamics of ground and excited state vibrational relaxation and energy transfer in transition metal carbonyls. J. Phys. Chem. B 2014, 118, 11781–11791. 10.1021/jp506326u. 25198700

[ref41] Park S.; Ji M. Ultrafast Vibrational Population Transfer Dynamics in 2-Acetylcyclopentanone Studied by 2D IR Spectroscopy. ChemPhysChem 2011, 12, 799–805. 10.1002/cphc.201000794. 21302339

[ref42] Eckert P. A.; Kubarych K. J. Vibrational coherence transfer illuminates dark modes in models of the FeFe hydrogenase active site. J. Chem. Phys. 2019, 151, 054307 10.1063/1.5111016.

[ref43] Wu Y.; Yu P.; Chen Y.; Zhao J.; Liu H.; Li Y.; Wang J. Intensified C≡C Stretching Vibrator and Its Potential Role in Monitoring Ultrafast Energy Transfer in 2D Carbon Material by Nonlinear Vibrational Spectroscopy. J. Phys. Chem. Lett. 2019, 10, 1402–1410. 10.1021/acs.jpclett.9b00027. 30848918

[ref44] Taylor V. C.; Tiwari D.; Duchi M.; Donaldson P. M.; Clark I. P.; Fermin D. J.; Oliver T. A. Investigating the Role of the Organic Cation in Formamidinium Lead Iodide Perovskite Using Ultrafast Spectroscopy. J. Phys. Chem. Lett. 2018, 9, 895–901. 10.1021/acs.jpclett.7b03296. 29389137

[ref45] Fedoseeva M.; Delor M.; Parker S. C.; Sazanovich I. V.; Towrie M.; Parker A. W.; Weinstein J. A. Vibrational energy transfer dynamics in ruthenium polypyridine transition metal complexes. Phys. Chem. Chem. Phys. 2015, 17, 1688–1696. 10.1039/C4CP04166F. 25463745

[ref46] Rubtsov I. V.; Hochstrasser R. M. Vibrational dynamics, mode coupling, and structural constraints for acetylproline-NH_2_. J. Phys. Chem. B 2002, 106, 9165–9171. 10.1021/jp020837b.

[ref47] Khalil M.; Demirdöven N.; Tokmakoff A. Coherent 2D IR spectroscopy: Molecular structure and dynamics in solution. J. Phys. Chem. A 2003, 107, 5258–5279. 10.1021/jp0219247.

[ref48] Brinzer T.; Berquist E. J.; Ren Z.; Dutta S.; Johnson C. A.; Krisher C. S.; Lambrecht D. S.; Garrett-Roe S. Ultrafast vibrational spectroscopy (2D-IR) of CO_2_ in ionic liquids: Carbon capture from carbon dioxide’s point of view. J. Chem. Phys. 2015, 142, 212425 10.1063/1.4917467. 26049445

[ref49] Kelsheimer C. J.; Garrett-Roe S. Intramolecular vibrational energy relaxation of CO_2_ in cross-linked poly(ethylene glycol) diacrylate-based ion gels. J. Phys. Chem. B 2021, 125, 1402–1415. 10.1021/acs.jpcb.0c06685. 32955891

[ref50] Leong T. X.; Collins B. K.; Dey Baksi S.; Mackin R. T.; Sribnyi A.; Burin A. L.; Gladysz J. A.; Rubtsov I. V. Tracking Energy Transfer across a Platinum Center. J. Phys. Chem. A 2022, 126, 4915–4930. 10.1021/acs.jpca.2c02017. 35881911 PMC9358659

[ref51] Hassani M.; Mallon C. J.; Monzy J. N.; Schmitz A. J.; Brewer S. H.; Fenlon E. E.; Tucker M. J. Inhibition of vibrational energy flow within an aromatic scaffold via heavy atom effect. J. Chem. Phys. 2023, 158, 224201 10.1063/5.0153760. 37309893 PMC10275622

[ref52] Demirdöven N.; Khalil M.; Tokmakoff A. Correlated Vibrational Dynamics Revealed by Two-Dimensional Infrared Spectroscopy. Phys. Rev. Lett. 2002, 89, 237401 10.1103/PhysRevLett.89.237401. 12485039

[ref53] Kwak K.; Rosenfeld D. E.; Fayer M. D. Taking apart the two-dimensional infrared vibrational echo spectra: More information and elimination of distortions. J. Chem. Phys. 2008, 128, 204505 10.1063/1.2927906. 18513030

[ref54] Kwak K.; Park S.; Finkelstein I. J.; Fayer M. D. Frequency-frequency correlation functions and apodization in two-dimensional infrared vibrational echo spectroscopy: A new approach. J. Chem. Phys. 2007, 127, 124503 10.1063/1.2772269. 17902917

[ref55] Yuan R.; Fayer M. D. Dynamics of Water Molecules and Ions in Concentrated Lithium Chloride Solutions Probed with Ultrafast 2D IR Spectroscopy. J. Phys. Chem. B 2019, 123, 7628–7639. 10.1021/acs.jpcb.9b06038. 31402658

[ref56] Duan R.; Mastron J. N.; Song Y.; Kubarych K. J. Direct comparison of amplitude and geometric measures of spectral inhomogeneity using phase-cycled 2D-IR spectroscopy. J. Chem. Phys. 2021, 154, 174202 10.1063/5.0043961. 34241049

[ref57] Guo Q.; Pagano P.; Li Y. L.; Kohen A.; Cheatum C. M. Line shape analysis of two-dimensional infrared spectra. J. Chem. Phys. 2015, 142, 212427 10.1063/1.4918350. 26049447 PMC4409623

[ref58] Robben K. C.; Cheatum C. M. Least-Squares Fitting of Multidimensional Spectra to Kubo Line-Shape Models. J. Phys. Chem. B 2021, 125, 12876–12891. 10.1021/acs.jpcb.1c08764. 34783568 PMC8630800

[ref59] Pagano P.; Guo Q.; Kohen A.; Cheatum C. M. Oscillatory Enzyme Dynamics Revealed by Two-Dimensional Infrared Spectroscopy. J. Phys. Chem. Lett. 2016, 7, 2507–2511. 10.1021/acs.jpclett.6b01154. 27305279 PMC4939886

[ref60] Frisch M. J.; Gaussian 09. Revision D.01, 2009. http://gaussian.com/.

[ref61] Frisch M. J.; Gaussian 16. Revision A.03, 2016. http://gaussian.com/.

