# Supplementary material for: Two-Dimensional Infrared Spectroscopy Resolves the Vibrational Landscape in Donor–Bridge–Acceptor Complexes with Site-Specific Isotopic Labeling
Source: ACS Phys Chem Au. 2024 Oct 29;4(6):761–72. doi: 10.1021/acsphyschemau.4c00073 (PMC11613348; doi:10.1021/acsphyschemau.4c00073)
Supplement: Supplementary file 1 — pg4c00073_si_001.pdf [file pg4c00073_si_001.pdf]

Supplementary Information for

Two-Dimensional Infrared Spectroscopy Resolves the Vibrational Landscape in Donor-Bridge-Acceptor Complexes with Site-Specific Isotopic Labelling

James D. Shipp,[a*‡] Ricardo J. Fernández-Terán,[ab*] Alexander J. Auty,[a], Heather Carson,[a] Andrew J. Sadler,[a] Mike Towrie,[c] Igor V. Sazanovich,[c] Paul M. Donaldson,[c] Anthony J. H. M. Meijer,[a] and Julia A. Weinstein[a*]

a) Department of Chemistry, University of Sheffield, S3 7HF, UK

b) Department of Physical Chemistry, University of Geneva. CH-1205, Geneva, Switzerland.

c) Central Laser Facility, Research Complex at Harwell, Rutherford Appleton Laboratory, Harwell Science and Innovation Campus, STFC, Chilton, Oxfordshire, OX11 0QX, UK

‡Current Address: Department of Chemistry, University of Pittsburgh, Chevron Science Center, 219 Parkman Ave, Pittsburgh, Pennsylvania, 15260, USA.

Email: james.shipp@pitt.edu, Ricardo.FernandezTeran@unige.ch, and Julia.Weinstein@sheffield.ac.uk

Table of Contents

1. Synthetic details 2
2. Computational Details 11
3. Ground State FTIR spectral analysis 13
4. Supplementary 2D-IR Spectra 16
5. Vibrational coupling between ν(CC)$_a$ and ν(CC)$_s$ 23
6. Kinetics 25
7. Spectral diffusion 28
8. Dynamic Anharmonicities 33
9. Oscillations in the kinetic traces of the NAP carbonyls 35
10. Experimental details for 2D-IR spectroscopy 35
11. References 37

1. Synthetic details

1.1 Materials and General Procedures

Commercially available starting materials were obtained from Sigma-Aldrich, AlfaAesar, Acros Organics, Manchester Organics, Fluorochem, Apollo Scientific and were used without further purification unless otherwise stated. All solvents (Fisher Scientific, Sigma-Aldrich, VWR) were HPLC grade and used without further purification unless otherwise stated. Dry solvents were obtained from the University of Sheffield Grubbs solvent purification system and stored under a nitrogen atmosphere. Solvent deaeration was performed by sparging with argon for a period of at least 20 min. Column chromatography was performed using either silica gel (60 Å mesh, Fluorochem) or aluminium oxide (Brockmann III grade, Acros). Unless otherwise stated, all syntheses were carried out under an atmosphere of argon using Schlenk line techniques.

1.2 Synthesis of [*cis*-$Pt(PBu_3)_2Cl_2$]

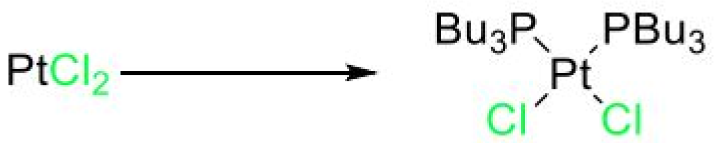

Tri-*n*-butylphosphine (1.85 ml, 1.52 g, 7.519 mmol) was added to a suspension of platinum(II) chloride (1.00 g, 3.760 mmol) in deaerated, dry CH_2Cl_2 (80 cm^3) and stirred at room temperature under an inert argon atmosphere for seven days. The reaction mixture was filtered to remove solid particulates and then the solvent was removed *in vacuo* to leave a grey solid. The solid was washed with hexane (50 cm^3), sonicated for one minute, and then filtered. The filter residue was washed with cold hexane (4 x 20 cm^3). The solid product was washed through the filter with diethyl ether until no solid remained (ca. 100 cm^3), and then the solvent was removed *in vacuo* to give the product as an off-white solid (2.01 g, 80 %).

Found: **^{1}H NMR** ($CDCl_3$, 250 MHz): 0.85-1.04 (m, 18H), 1.34-1.66 (m, 24H), 1.75-2.17 (m, 12H); **^{31}P NMR** ($CDCl_3$, 162 MHz): 0.95 (J_{Pt-P} = 3518 Hz).

1.3 Synthesis of 4-bromo-N-octyl-1,8-naphthalimide

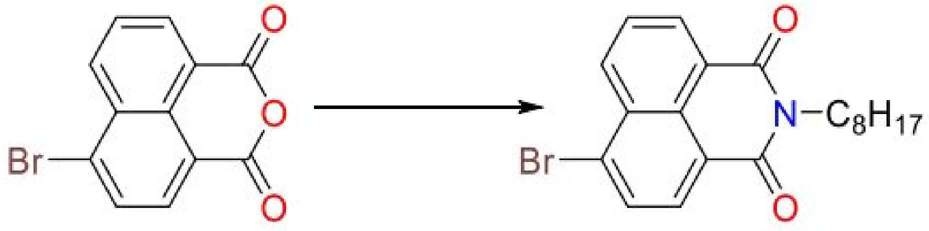

4-bromo-1,8-naphthalic anhydride (4.9 g, 17.7 mmol) and n-octylamine (6.05 g, 7.8 cm^3, 46.8 mmol) were added to deaerated isopropanol (100 cm^3). The reaction mixture was then heated under reflux for 24 hours. The volume of the reaction solution was reduced *in vacuo* and then cooled to 0 °C. Methanol (*ca*. 200 ml) was added to induce precipitation of the product which was then collected by vacuum filtration and washed with cold methanol (3 x 50 cm^3) until the filtrate stopped staining thin layer silica plates yellow. The pale-yellow solid was dried *in vacuo* to give the product, 4-bromo-N-octyl-1,8-naphthalimide (5.25 g, 76 %).

Found: ^{1}**H NMR** (400 MHz, $CDCl_3$) δ 8.66 (d, J = 7.2 Hz, 1H), 8.57 (d, J = 8.4 Hz, 1H), 8.41 (d, J = 7.8 Hz, 1H), 8.04 (d, J = 7.8 Hz, 1H), 7.89 – 7.81 (m, 1H), 4.22 – 4.05 (m, 2H), 1.72 (dt, J = 15.1, 7.5 Hz, 2H), 1.48 – 1.14 (m, 10H), 0.87 (t, J = 6.7 Hz, 3H). **ESMS**: m/z = 388.1 (MH+, 100 %).

1.4 Synthesis of 4-trimethylsilyl-C≡C-N-octyl-1,8-naphthalimide

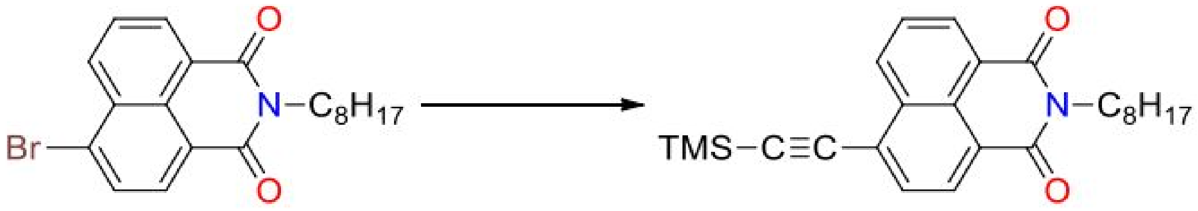

4-bromo-N-octyl-1,8-naphthalimide (1.01 g, 2.601 mmol), copper(I) iodide (51 mg, 0.267 mmol, 10 mol%), bis(triphenylphosphine)palladium chloride (91 mg, 0.130 mmol, 5 mol%) and triphenylphosphine (34 mg, 0.130 mmol, 5.0 mol%) were added to deaerated triethylamine (45 cm^3) and stirred at room temperature for 15 minutes. Trimethylsilylacetylene (1.48 cm^3, 0.69 g cm^{-3}, 10.4 mmol) was added which resulted in a colour change from yellow to dark brown/black. The reaction solution was heated at 75 °C for 48 hours. The reaction mixture was then allowed to cool to room temperature, then the solvent was removed *in vacuo*. The crude product was then dissolved in diethyl ether and passed down through a silica plug to yield a bright yellow filtrate which was dried *in vacuo* to give 4-trimethylsilyl-C≡C-N-octyl-1,8-naphthalimide (0.95 g, 90 %).

Found: ^{1}**H NMR** (400 MHz, $CDCl_3$) δ 8.62 (d, J = 2.0 Hz, 1H), 8.60 (d, J = 0.9 Hz, 1H), 8.49 (d, J = 7.6 Hz, 1H), 7.87 (d, J = 7.6 Hz, 1H), 7.83 – 7.77 (m, 1H), 4.18 – 4.12 (m, 2H), 1.72 (dt, J = 15.3, 7.5 Hz, 3H), 1.49 – 1.18 (m, 14H), 0.91 – 0.82 (m, 4H), 0.36 (s, 9H).

1.5 Synthesis of 4-trimethylsilyl-$^{13}C{\equiv}^{13}C$-N-octyl-1,8-naphthalimide

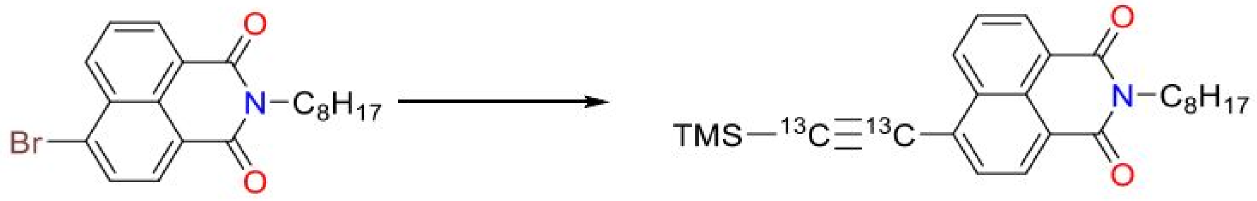

4-bromo-N-octyl-1,8-naphthalimide (0.28 g, 0.723 mmol), copper(I) iodide (14 mg, 0.072 mmol, 10 mol%), bis(triphenylphosphine)palladium chloride (25 mg, 0.036 mmol, 5 mol%) and triphenylphosphine (9.48 mg, 0.036 mmol, 5 mol%) were added to deaerated trimethylamine (20 cm^3) and stirred at room temperature for 15 minutes. Carbon-13 labelled trimethylsilylacetylene (0.42 cm^3, 0.69 g cm^{-3}, 2.89 mmol) was added which resulted in a colour change from yellow to dark brown/black. The reaction solution was heated at 75 °C for 48 hours, and then allowed to cool to room temperature. The solvent was then removed *in vacuo*. The crude product was dissolved in diethyl ether and passed through a silica plug to yield a bright yellow filtrate which was dried *in vacuo* to give 4-trimethylsilyl-$^{13}C{\equiv}^{13}C$-N-octyl-1,8-naphthalimide (0.22 g, 75 %).

Found: **^{1}H NMR** (400 MHz, $CDCl_3$) δ 8.63 (d, J = 1.8 Hz, 1H), 8.61 (s, 1H), 8.51 (d, J = 7.6 Hz, 1H), 7.89 (dd, J = 7.6, 5.6 Hz, 1H), 7.84 – 7.78 (m, 1H), 4.19 – 4.12 (m, 2H), 1.80 – 1.64 (m, 2H), 1.48 – 1.19 (m, 10H), 0.95 – 0.78 (m, 3H), 0.36 (s, 9H).

1.6 Synthesis of 4-C≡CH-N-octyl-1,8-naphthalimide

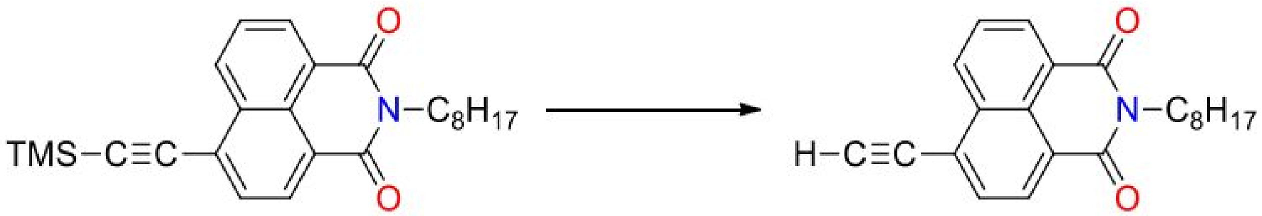

4-trimethylsilyl-C≡C-N-octyl-1,8-naphthalimide (0.95 g, 2.342 mmol) was added to a suspension of potassium carbonate (1.424 g, 10.30 mmol) in deaerated 1:2 (v/v) MeOH / CH_2Cl_2 (50 cm^3), which was then stirred at room temperature for 18 hours. Water (60 cm^3) was added to the reaction solution to quench the reaction. Then the organic solvent was removed *in vacuo* to give an aqueous suspension of the product. The product was then extracted with CH_2Cl_2 (2 x 100 cm^3). The organic layer was washed with water (50 cm^3) and then dried over anhydrous $MgSO_4$. The volume of the product containing solution was reduced *in vacuo* and then the product was purified by column chromatography (SiO_2, CH_2Cl_2) to yield a yellow solid (0.75 g, 96 %).

Found: ^{1}**H NMR** (400 MHz, $CDCl_3$) δ 8.61 (t, J = 6.9 Hz, 1H), 8.49 (d, J = 7.6 Hz, 1H), 7.90 (d, J = 7.6 Hz, 1H), 7.83 – 7.74 (m, 1H), 4.18 – 4.10 (m, 2H), 3.73 (s, 1H), 1.72 (dt, J = 15.3, 7.5 Hz, 2H), 1.46 – 1.18 (m, 10H), 0.86 (t, J = 6.8 Hz, 3H). **ESMS**: m/z = 334.1 (MH+, 100 %).

1.7 Synthesis of 4-trimethylsilyl-$^{13}C\equiv^{13}CH$-N-octyl-1,8-naphthalimide

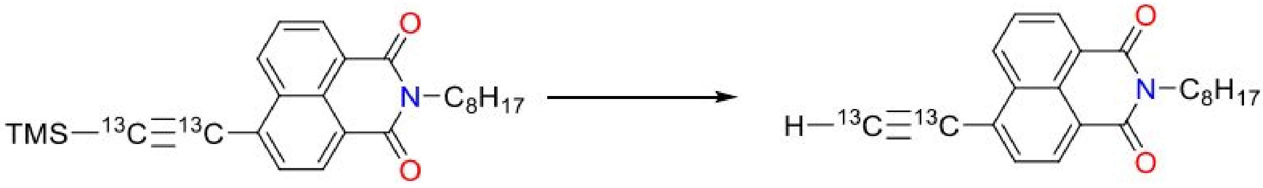

4-TMS-$^{13}C\equiv^{13}C$-N-octyl-1,8-naphthalimide (0.22 g, 0.540 mmol) was added to a suspension of potassium carbonate (0.40 g, 2.89 mmol) in deaerated 1:2 (v/v) MeOH / CH_2Cl_2 (20 cm^3). The reaction mixture was stirred at room temperature for 18 hours. Water (25 cm^3) was then added to the reaction solution to quench the reaction. Then the organic solvent was removed *in vacuo* to give an aqueous suspension of the product. The product was then extracted with CH_2Cl_2 (2 x 50 cm^3), the organic layer was washed with water (40 cm^3) and then dried over anhydrous $MgSO_4$. The volume of the organic solution was reduced *in vacuo*, then the product was purified by column chromatography (SiO_2, CH_2Cl_2) to yield a yellow solid (167 mg, 92%).

Found: ^{1}**H NMR** (400 MHz, $CDCl_3$) δ 8.62 (t, J = 7.6 Hz, 2H), 8.51 (d, J = 7.6 Hz, 1H), 7.91 (dd, J = 7.5, 6.0 Hz, 1H), 7.84 – 7.77 (m, 1H), 4.19 – 4.12 (m, 2H), 3.72 (dd, J = 249.9, 53.9 Hz, 1H), 1.72 (dt, J = 15.3, 7.5 Hz, 2H), 1.48 – 1.14 (m, 10H), 0.86 (t, J = 6.8 Hz, 3H). **ESMS**: m/z = 336.2 (MH+, 100 %).

1.8 Synthesis of N-(4-iodobenzyl)-phenothiazine

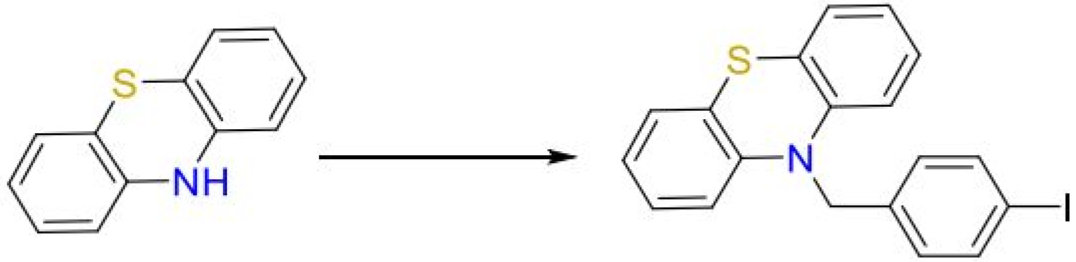

Diisopropylamine (1.8 cm^3, 0.722 g cm^{-3}, 12.843 mmol) was deaerated by purging with argon for 30 minutes. It was then added to dry deaerated THF (60 cm^3) and cooled in an ice bath to 0 °C. n-butyllithium (in hexanes) (4.95 cm^3, 2.5 mol dm^{-3}, 12.375 mmol) was then added dropwise over five minutes. The reaction mixture was then stirred at 0 °C for 45 minutes. Phenothiazine (2.286 g, 11.469 mmol) was dissolved in dry deaerated THF (60 cm^3) and added to the reaction vessel by cannula transfer to give a bright yellow solution.

The reaction mixture was then stirred at 0 °C for 10 minutes before allowing to warm to room temperature. 4-iodobenzylbromide (3.78 g, 12.729 mmol) was dissolved in dry deaerated THF (60 cm^3), this solution was then added to the reaction mixture by cannula transfer. The reaction mixture was then stirred at room temperature for 10 days. Removal of the solvent *in vacuo* yielded an oily residue which was redissolved in CH_2Cl_2 (250 cm^3) and poured quickly into a saturated aqueous NaCl solution (350 cm^3). The organic phase was washed with brine (200 cm^3) and water (200 cm^3) before being dried over anhydrous $MgSO_4$. The product containing solution was then filtered and the solvent was removed *in vacuo* to yield a light brown oily solid. The product was purified by column chromatography (SiO_2, 1:10 CH_2Cl_2: hexane) to yield the purified product as a clear oily solid (3.53 g, 74 %).

Found: **^{1}H NMR** (400 MHz, $CDCl_3$) δ 7.63 (d, J = 8.35 Hz, 2H), 7.04-7.10 (m, 4H), 6.97 (td, J = 1.60, 7.71 Hz, 2H), 6.86 (td, J = 1.03, 7.47 Hz, 2H), 6.60 (dd, J = 0.87, 8.11 Hz, 2H), 5.00 (s, 2H).

1.9 Synthesis of N-(4-trimethylsilyl-C≡C-benzyl)-phenothiazine

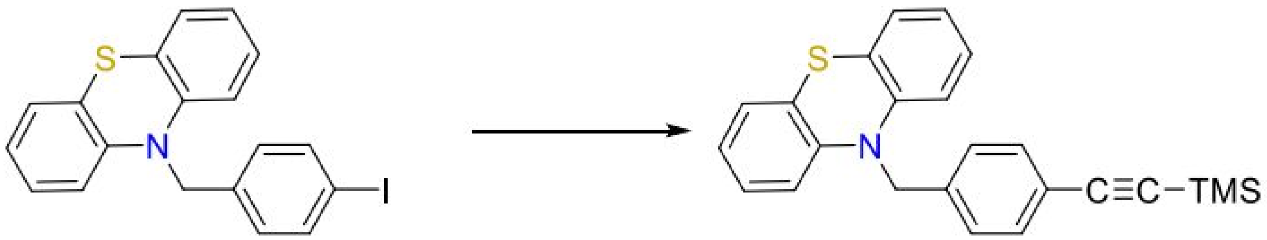

N-(4-iodobenzyl)-phenothiazine (1.00 g, 2.410 mmol), bis(triphenylphosphine) palladium chloride (90 mg, 0.128 mmol, 5 mol %), and copper iodide (53 mg, 0.278 mmol, 12 mol %) were dissolved in deaerated benzene (50 cm^3). Trimethylsilylacetylene (1.35 cm^3, 0.69 g cm^{-3}, 9.552 mmol) was added slowly to the solution over 10 minutes whilst stirred at room temperature. Distilled, deaerated triethylamine (3.5 cm^{-3}, 0.726 g cm^{-3}, 25.1 mmol) was added to the reaction mixture, resulting in a colour change from orange to dark brown. The reaction mixture was stirred at room temperature for five days before the solvent was removed *in vacuo* and the product purified by column chromatography (SiO_2, 1:5 CH_2Cl_2: hexane) to yield the product as a white solid (0.78 g, 84 %).

Found: **^{1}H NMR** (400 MHz, $CDCl_3$) δ 7.42 (d, J = 8.28 Hz, 2H), 7.25 (d, J = 8.16 Hz, 2H), 7.09 (dd, J = 1.56, 7.56 Hz, 2H), 6.96 (td, J = 1.60, 7.68 Hz, 2H), 6.86 (td, J = 1.08, 7.44 Hz, 2H), 6.59 (dd, J = 0.96, 8.16 Hz, 2H), 5.06 (s, 2H), 0.23 (s, 9H).

1.10 Synthesis of N-(4-trimethylsilyl-13C≡13C-benzyl)-phenothiazine

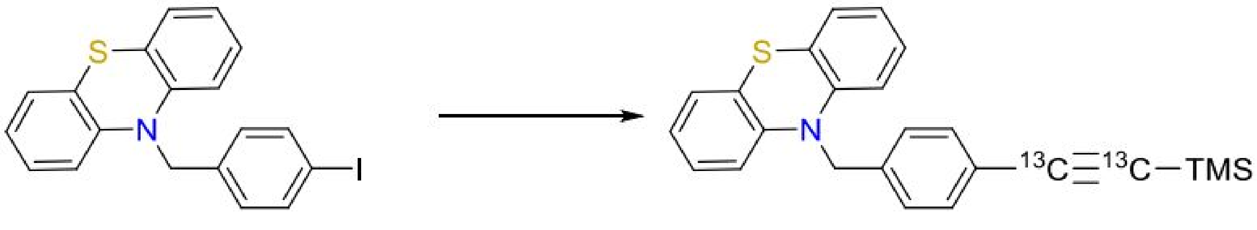

N-(4-iodobenzyl)-phenothiazine (0.25 g, 0.60 mmol), bis(triphenylphosphine) palladium chloride (2 mg, 0.03 mmol, 5 mol%), and copper iodide (14 mg, 0.07 mmol, 12 mol%) were dissolved in deaerated benzene (50 cm^3). Carbon-13 labelled trimethylsilylacetylene (0.35 cm^3, 0.24 g, 2.4 mmol) was added slowly to the solution whilst it was stirred at room temperature over 10 minutes. Distilled, deaerated triethylamine (0.84 cm^3, 6 mmol) was added to the reaction mixture resulting in a colour change from orange to dark brown. The reaction mixture was stirred at room temperature for five days before the solvent was removed *in vacuo* and the product purified by column chromatography (SiO_2, 1:5 CH_2Cl_2: hexane) to yield the product as a white solid (216 mg, 88 %).

Found: **^{1}H NMR** (400 MHz, $CDCl_3$) δ 7.42 (d, J = 8.28 Hz, 2H), 7.25 (d, J = 8.16 Hz, 2H), 7.09 (dd, J = 1.56, 7.56 Hz, 2H), 6.96 (td, J = 1.60, 7.68 Hz, 2H), 6.86 (td, J = 1.08, 7.44 Hz, 2H), 6.59 (dd, J = 0.96, 8.16 Hz, 2H), 5.06 (s, 2H), 0.23 (s, 9H).

1.11 Synthesis of N-(4-C≡CH-benzyl)-phenothiazine

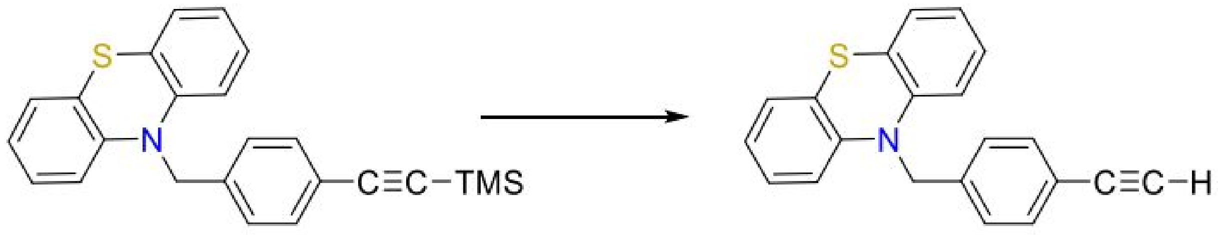

N-(4-trimethylsilyl-C≡C-benzyl)-phenothiazine (0.78 g, 2.02 mmol) was added to a suspension of potassium carbonate (0.7 g, 5.05 mmol) in deaerated 1:1 MeOH:THF (v/v) (50 cm^3). The reaction mixture was stirred rapidly for 24 hours at room temperature. Water (40 cm^3) was added to quench the reaction mixture and then the organic solvent was removed *in vacuo* to give a solid foam like substance suspended in the water. The product was extracted from the aqueous suspension with CH_2Cl_2 (3 x 100 cm^3). The combined organic layers were dried over anhydrous $MgSO_4$ then the solvent was removed *in vacuo* to give the crude product as a brown oil. The crude product was purified by column chromatography (SiO_2, 1:9 CH_2Cl_2: hexane) to yield N-(4-C≡CH-benzyl)-phenothiazine as a clear oil (0.56 g, 89 %).

Found: **^{1}H NMR** (400 MHz, $CDCl_3$) δ 7.44 (d, J = 8.23 Hz, 2H), 7.27 (d, J = 8.34 Hz, 2H), 7.09 (dd, J = 1.56, 7.52 Hz, 2H), 6.96 (td, J = 1.60, 7.51 Hz, 2H), 6.86 (td, J = 1.11, 7.46 Hz, 2H), 6.59 (dd, J = 0.96, 8.16 Hz, 2H), 5.06 (s, 2H), 3.05 (s, 1H). **ESMS**: m/z = 314.1 (MH+, 100 %).

1.12 Synthesis of N-(4-$^{13}C{\equiv}^{13}CH$-benzyl)-phenothiazine

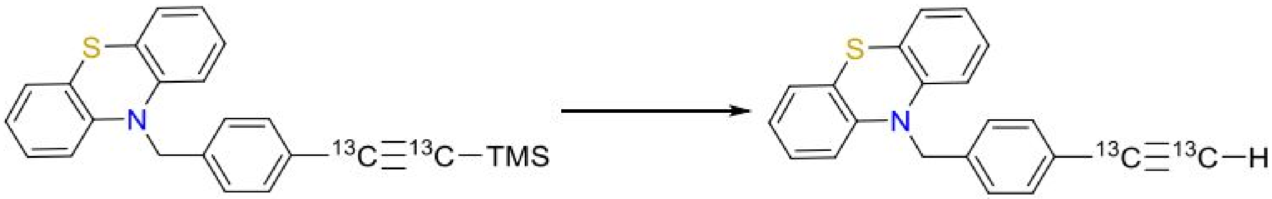

N-(4-trimethylsilyl-$^{13}C{\equiv}^{13}C$-benzyl)-phenothiazine (0.216 g, 0.56 mmol) was added to a suspension of potassium carbonate (193 mg, 1.4 mmol) in deaerated 1:1 MeOH:THF (v/v) (50 cm^3). The reaction mixture was stirred rapidly for 24 hours at room temperature. Water (40 cm^3) was added to quench the reaction mixture, and then the organic solvent was removed *in vacuo* to give a solid foam like substance suspended in the water. The product was extracted from the aqueous suspension with CH_2Cl_2 (3 x 100 cm^{-3}). The combined organic layers were dried over anhydrous $MgSO_4$ then the solvent was removed *in vacuo* to give the crude product as a brown oil. The crude product was purified by column chromatography (SiO_2, 1:9 CH_2Cl_2: hexane) to yield N-(4-$^{13}C{\equiv}^{13}CH$-benzyl)-phenothiazine as a clear oil (158 mg, 91 %).

Found: **^{1}H NMR** (400 MHz, CDCl3) δ 7.44 (d, J = 8.23 Hz, 2H), 7.27 (d, J = 8.34 Hz, 2H), 7.09 (dd, J = 1.56, 7.52 Hz, 2H), 6.96 (td, J = 1.60, 7.51 Hz, 2H), 6.86 (td, J = 1.11, 7.46 Hz, 2H), 6.59 (dd, J = 0.96, 8.16 Hz, 2H), 5.06 (s, 2H), 3.05 (s, 1H). **ESMS**: m/z = 316.1 (MH+, 100 %).

1.13 Synthesis of [*trans*-Pt(PBu3)2(4-C≡C-N-octyl-1,8-naphthalimide)Cl]

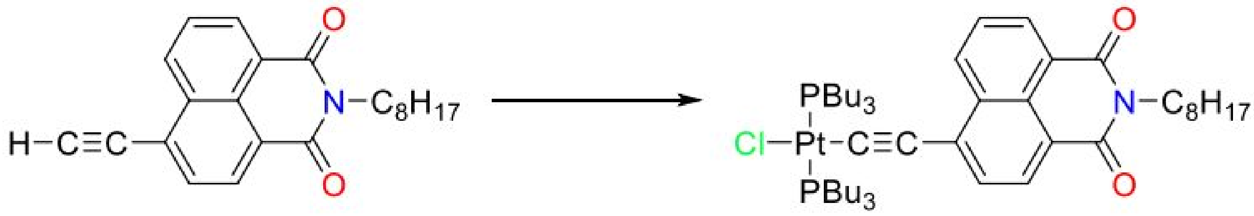

4-C≡CH-N-octyl-1,8-naphthalimide (178 mg, 0.532 mmol) was dissolved in distilled deaerated diisopropylamine (50 cm^3), to this [*cis*-$Pt(PBu_3)_2Cl_2$] (0.5 g, 0.746 mmol) was added and the reaction mixture was refluxed at 84 °C in the dark for 20 hours. The reaction mixture was allowed to cool to room temperature. Then the solvent was removed *in vacuo* to give the crude product as an orange solid. The crude product was purified by column chromatography (SiO_2, CH_2Cl_2) to give the product, [$Pt(PBu_3)_2Cl(C{\equiv}C\text{-}NAPC_8H_{17})$], as the first bright yellow band. The second band to elute was the dimeric side product, [$Pt(PBu_3)_2(C{\equiv}C\text{-}NAP\text{-}C_8H_{17})_2$]. The solvent was removed *in vacuo* to give [$Pt(PBu_3)_2Cl(C{\equiv}C\text{-}NAP\text{-}C_8H_{17})$] as a bright yellow film (268 mg, 52 %).

Found: **^{1}H NMR** (400 MHz, $CDCl_3$) δ 8.72 (dd, J = 8.3, 1.1 Hz, 1H), 8.57 (dd, J = 7.3, 1.0 Hz, 1H), 8.45 (d, J = 7.7 Hz, 1H), 7.67 (dd, J = 8.2, 7.4 Hz, 1H), 7.58 (d, J = 7.7 Hz, 1H), 4.20 – 4.13 (m, 2H), 2.10 – 1.91 (m,

12H), 1.72 (dt, J = 15.3, 7.5 Hz, 2H), 1.67 – 1.49 (m, 12H), 1.49 – 1.19 (m, 22H), 0.98 – 0.79 (m, 21H). **APMS**: m/z = 967.5 (MH+, 100 %).

1.14 Synthesis of [*trans*-Pt(PBu_3)$_2$(4-$^{13}C{\equiv}^{13}C$-N-octyl-1,8-naphthalimide)Cl]

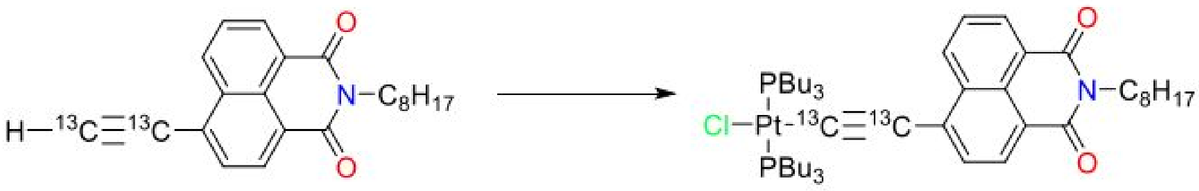

4-$^{13}C{\equiv}^{13}CH$-N-octyl-1,8-naphthalimide (167 mg, 0.498 mmol) was dissolved in distilled deaerated diisopropylamine (50 cm^3), to this [*cis*-Pt(PBu_3)$_2$$Cl_2$] (468 mg, 0.697 mmol) was added and the reaction mixture was refluxed at 84 °C in the dark for 24 hours. The reaction mixture was allowed to cool to room temperature and the solvent removed *in vacuo* to give the crude product as an orange solid. The crude product was purified by column chromatography (SiO_2, CH_2Cl_2) to give the product [Pt(PBu_3)$_2$Cl($^{13}C{\equiv}^{13}C$-NAP-C_8H_{17})] as the first bright yellow band. The second band to elute was the dimeric [Pt(PBu_3)$_2$($^{13}C{\equiv}^{13}C$-NAP-C_8H_{17})$_2$] complex. The solvent was removed *in vacuo* to give [Pt(PBu_3)$_2$Cl($^{13}C{\equiv}^{13}C$-NAP-C_8H_{17})] as a bright yellow film (212 mg, 44 %).

Found: **^{1}H NMR** (400 MHz, $CDCl_3$) δ 8.72 (dd, J = 8.3, 1.1 Hz, 1H), 8.57 (dd, J = 7.3, 1.0 Hz, 1H), 8.45 (d, J = 7.7 Hz, 1H), 7.67 (dd, J = 8.2, 7.4 Hz, 1H), 7.57 (dd, J = 7.6, 5.1 Hz, 1H), 4.20 – 4.13 (m, 2H), 2.10 – 1.91 (m, 12H), 1.72 (dt, J = 15.3, 7.5 Hz, 2H), 1.67 – 1.49 (m, 12H), 1.49 – 1.19 (m, 22H), 0.98 – 0.79 (m, 21H). **APMS**: m/z = 969.5 (MH+, 100 %).

1.15 Synthesis of [*trans*-Pt(PBu_3)$_2$(4-C≡C-N-octyl-1,8-naphthalimide)(N-(4-C≡C-benzyl)-phenothiazine]

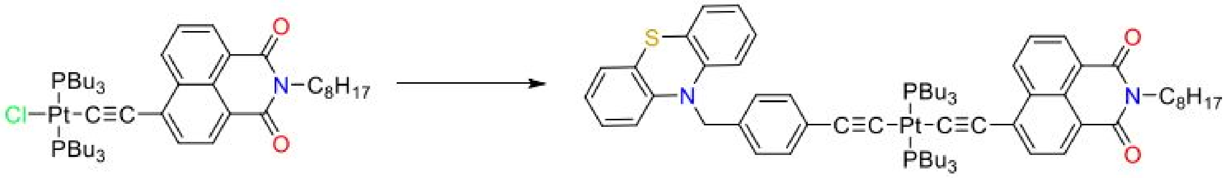

[Pt(PBu_3)$_2$Cl(C≡C-NAP-C_8H_{17})] (100 mg, 0.1 mmol) and N-(4-C≡CH-benzyl)-phenothiazine (93 mg, 0.03 mmol) were both dissolved in diisopropylamine (15 cm^3) in separate flasks and deaerated by purging with argon. The solution of N-(4-C≡CHbenzyl)-phenothiazine was added to the solution of [Pt(PBu_3)$_2$Cl(C≡C-NAP-C_8H_{17})] by cannula transfer. Then copper(I) iodide (6 mg, 0.03 mmol) was added. The reaction mixture was heated in the dark to 40 °C with stirring for 24 hours. The solvent was reduced in vacuo to give an orange/brown solid as the crude product which was then purified by column chromatography (SiO_2, 9:1 CH_2Cl_2: hexane). The product eluted first as a bright yellow band.

Found: ^{1}**H NMR** (400 MHz, $CDCl_3$) δ 8.77 (dd, J = 8.3, 1.1 Hz, 1H), 8.57 (dd, J = 7.3, 1.1 Hz, 1H), 8.44 (d, J = 7.7 Hz, 1H), 7.66 (dd, J = 8.1, 7.5 Hz, 1H), 7.59 (d, J = 7.8 Hz, 1H), 7.25 – 7.21 (m, 2H), 7.15 (d, J = 8.1 Hz, 2H), 7.08 (dd, J = 7.5, 1.5 Hz, 2H), 6.97 (td, J = 8.1, 1.6 Hz, 2H), 6.86 (td, J = 7.5, 1.0 Hz, 2H), 6.64 (dd, J = 8.1, 0.8 Hz, 2H), 5.03 (s, 2H), 4.18 – 4.13 (m, 2H), 2.21 – 2.03 (m, 12H), 1.77 – 1.67 (m, 2H), 1.67 – 1.50 (m, 12H), 1.50 – 1.19 (m, 22H), 0.96 – 0.81 (m, 21H); ^{31}**P NMR** (162 MHz, $CDCl_3$) δ 3.80 ($J_{Pt\text{-}P}$ = 2332 Hz).

1.16 Synthesis of [*trans*-Pt(PBu$_3$)$_2$(4-C≡C-N-octyl-1,8-naphthalimide)(N-(4-^{13}C≡^{13}C-benzyl)-phenothiazine]

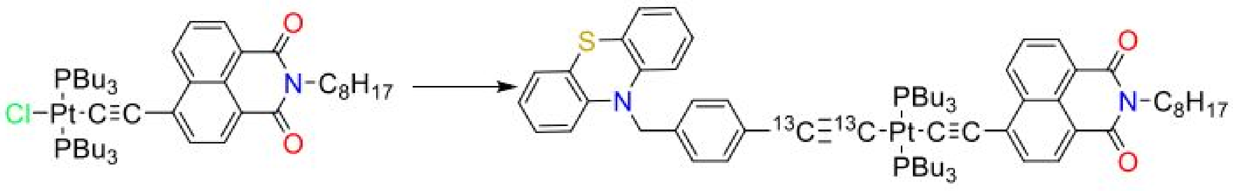

[Pt(PBu$_3$)$_2$Cl(C≡C-NAP-C$_8$H$_{17}$)] (105 mg, 0.109 mmol) and N-(4-^{13}C≡^{13}CH-benzyl)-phenothiazine (95 mg, 0.03 mmol) were both dissolved in diisopropylamine (15 cm^3) in separate flasks and deaerated by purging with argon. The solution of N-(4-^{13}C≡^{13}CH-benzyl) phenothiazine was added to the solution of [Pt(PBu$_3$)$_2$Cl(C≡C-NAP-C$_8$H$_{17}$) by cannula transfer. Then copper(I) iodide (6 mg, 0.03 mmol) was added. The reaction mixture was heated in the dark to 40 °C with stirring for 24 hours. The volume of the solvent was reduced *in vacuo* to give an orange/brown solid as the crude product which was then purified by column chromatography (SiO_2, 9:1 CH_2Cl_2: hexane). The product eluted first as a bright yellow band.

Found: ^{1}**H NMR** (400 MHz, $CDCl_3$) δ 8.77 (dd, J = 8.3, 1.1 Hz, 1H), 8.57 (dd, J = 7.3, 1.1 Hz, 1H), 8.44 (d, J = 7.7 Hz, 1H), 7.66 (dd, J = 8.1, 7.5 Hz, 1H), 7.59 (d, J = 7.8 Hz, 1H), 7.25 – 7.21 (m, 2H), 7.15 (d, J = 8.1 Hz, 2H), 7.08 (dd, J = 7.5, 1.5 Hz, 2H), 6.97 (td, J = 8.1, 1.6 Hz, 2H), 6.86 (td, J = 7.5, 1.0 Hz, 2H), 6.64 (dd, J = 8.1, 0.8 Hz, 2H), 5.03 (s, 2H), 4.18 – 4.13 (m, 2H), 2.21 – 2.03 (m, 12H), 1.77 – 1.67 (m, 2H), 1.67 – 1.50 (m, 12H), 1.50 – 1.19 (m, 22H), 0.96 – 0.81 (m, 21H); ^{31}**P NMR** (162 MHz, $CDCl_3$) δ 3.80 ($J_{Pt\text{-}P}$ = 2330 Hz).

1.17 Synthesis of [*trans*-Pt(PBu$_3$)$_2$(4-^{13}C≡^{13}C-N-octyl-1,8-naphthalimide)(N-(4-C≡C-benzyl)-phenothiazine]

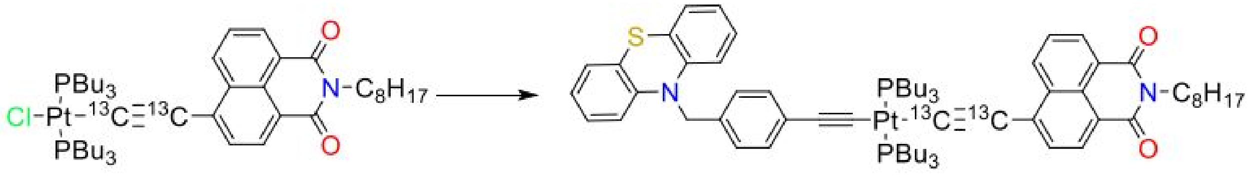

[Pt(PBu$_3$)$_2$Cl(^{13}C≡^{13}C-NAP-C$_8$H$_{17}$)] (110 mg, 0.113 mmol) and N-(4-C≡CH-benzyl) phenothiazine (95 mg, 0.3 mmol) were both dissolved in diisopropylamine (15 cm^3) in separate flasks and deaerated by purging with argon. The solution of N-(4-C≡CHbenzyl) phenothiazine was added to the solution of [Pt(PBu$_3$)$_2$Cl(^{13}C≡^{13}C-NAP-C$_8$H$_{17}$)] by cannula transfer and copper(I) iodide (6 mg, 0.03 mmol) was added. The reaction mixture

was heated in the dark to 40 °C with stirring for 24 hours. The solvent was reduced *in vacuo* to give an orange/brown solid as the crude product which was purified by column chromatography (SiO_2, 9:1 CH_2Cl_2: hexane). The product eluted first as a bright yellow band.

Found: **^{1}H NMR** (400 MHz, $CDCl_3$) δ 8.77 (dd, J = 8.3, 1.1 Hz, 1H), 8.57 (dd, J = 7.3, 1.1 Hz, 1H), 8.44 (d, J = 7.7 Hz, 1H), 7.66 (dd, J = 8.1, 7.5 Hz, 1H), 7.59 (dd, J = 7.6, 5.1 Hz, 1H), 7.25 – 7.21 (m, 2H), 7.15 (d, J = 8.1 Hz, 2H), 7.08 (dd, J = 7.5, 1.5 Hz, 2H), 6.97 (td, J = 8.1, 1.6 Hz, 2H), 6.86 (td, J = 7.5, 1.0 Hz, 2H), 6.64 (dd, J = 8.1, 0.8 Hz, 2H), 5.03 (s, 2H), 4.18 – 4.13 (m, 2H), 2.21 – 2.03 (m, 12H), 1.77 – 1.67 (m, 2H), 1.67 – 1.50 (m, 12H), 1.50 – 1.19 (m, 22H), 0.96 – 0.81 (m, 21H); **^{31}P NMR** (162 MHz, $CDCl_3$) δ 3.80 (J_{Pt-P} = 2330 Hz).

1.18 Synthesis of [*trans*-Pt(PBu_3)$_2$(4-^{13}C≡^{13}C-N-octyl-1,8-naphthalimide)(N-(4- C13≡C13-benzyl)-phenothiazine]

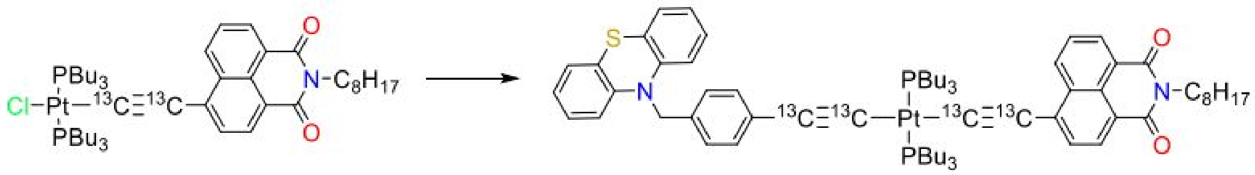

[Pt(PBu_3)$_2$Cl(^{13}C≡^{13}C-NAP-C_8H_{17})] (102 mg, 0.105 mmol) and N-(4-^{13}C≡^{13}CH-benzyl)-phenothiazine (95 mg, 0.3 mmol) were both dissolved in diisopropylamine (15 cm^3) in separate flasks and deaerated by purging with argon. The solution of N-(4-^{13}C≡^{13}CH-benzyl)-phenothiazine was added to the solution of [Pt(PBu_3)$_2$Cl(^{13}C≡^{13}C-NAP-C_8H_{17})] by cannula transfer. Copper(I) iodide (6 mg, 0.03 mmol) was then added and the reaction mixture was heated in the dark to 40 °C with stirring for 24 hours. The solvent was then reduced *in vacuo* to give an orange/brown solid as the crude product which was purified by column chromatography (SiO_2, 9:1 CH_2Cl_2: hexane). The product eluted first as a bright yellow band.

Found: **^{1}H NMR** (400 MHz, $CDCl_3$) δ 8.77 (dd, J = 0.96, 8.28 Hz, 1H), 8.56 (dd, J = 0.92, 7.24 Hz, 1H), 8.44 (d, J = 7.72 Hz, 1H), 7.66 (t, J = 7.84 Hz, 1H), 7.59 (dd, J = 7.6, 5.1 Hz, 1H), 7.24 (d, J = 8.20 Hz, 2H), 7.15 (d, J = 8.20 Hz, 2H), 7.07 (dd, J = 1.48, 7.56 Hz, 2H), 6.97 (td, J = 1.48, 7.86 Hz, 2H), 6.85 (td, J = 0.88, 7.44 Hz, 2H), 6.63 (d, J = 8.16 Hz, 2H), 5.03 (s, 2H), 4.15 (t, J = 7.64 Hz, 2H) 2.05-2.20 (m, 12H), 1.67-1.76 (m, 2H), 1.58-1.67 (m, 12H), 1.20-1.47 (m, 22H), 0.84-0.92 (m, 21H); **^{31}P NMR** (162 MHz, $CDCl_3$) δ 3.80 (J_{Pt-P} = 2330 Hz).

2. Computational Details

Geometry optimisations and harmonic frequency calculations were performed at the University of Sheffield using the SMP version of the Gaussian 09 package, revision D.01.[1] Gaussian was compiled using the Gaussian supplied version of BLAS and ATLAS on the EMT64 architecture.[2,3] The solvent, dichloromethane, was simulated using the integral equation formalism polarisable continuum model (IEFPCM) using the parameters as implemented in Gaussian 09.[4–6] All calculations utilized the PBE0 functional.[7] The Karlsruhe basis set, def2-SVP was employed for all atoms except Pt.[8,9] For these, the Dirac-Hartree-Fock basis set, dhf-SVP,[10] was used instead. Frequencies within the harmonic approximation were calculated for all optimised structures. The absence of imaginary frequencies confirmed that energy minima had been found. For all calculations,

ultrafine integrals were used and no symmetry constraints were applied. Calculated vibrational frequencies were scaled to account for the anharmonicity of the vibrational modes.[11–15]

Anharmonic calculations were performed in the Yggdrasil HPC cluster of the University of Geneva on the reduced subspace of the acetylide stretching, C=O stretching and Ar_{NAP} normal modes (labels as shown in Figures 2 and 3 in the main text). The anharmonic treatment of the vibrations was performed using the Gaussian 16 rev. A.03 implementation of the generalized second-order vibrational perturbation theory (GVPT2), including terms up to the third and fourth derivatives of the potential energy with respect to the normal mode coordinates.[16,17]

2.1. Anharmonic coupling matrices

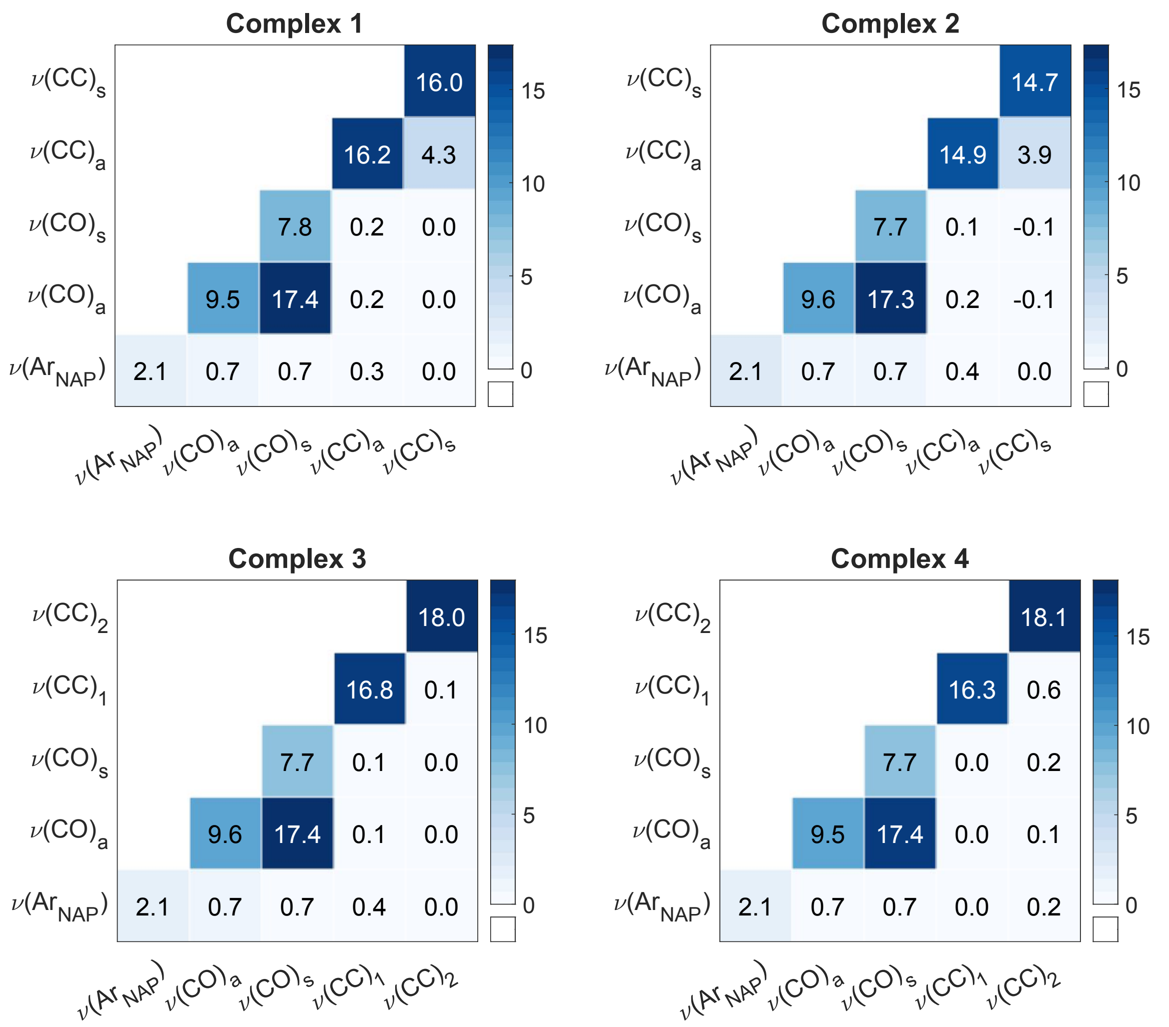

Figure S1 – Anharmonic coupling matrices obtained from Anharmonic frequency calculations in the reduced spaces of the indicated modes. Couplings are given in cm⁻¹ and are calculated as: $\Delta_{ij} = \omega_i + \omega_j - \omega_{ij}$, where ω_{ij} is the frequency of either the overtone (diagonal, $i = j$) or the combination mode (off-diagonal, $i \neq j$). Values smaller than 0.5 cm⁻¹ can be safely considered equal to zero.

3. Ground State FTIR spectral analysis

3.1 Reduced mass analysis for 1 and 2

The effect of $^{12}C \rightarrow {}^{13}C$ isotopic substitution on the acetylide stretching vibrational frequency was estimated with reduced mass analysis. This calculation accurately reproduced the observed shift in vibrational frequency.

Hooke's Law:

$$\nu = \frac{1}{2\pi c}\sqrt{\frac{k}{\mu}} \qquad \text{Eq. S1}$$

Reduced mass formula:

$$\mu = \frac{m_1 \cdot m_2}{m_1 + m_2} \qquad \text{Eq. S2}$$

$$m_1 = m_2 = 12\ gmol^{-1} \qquad \text{Eq. S3}$$

$$m_3 = m_4 = 13\ g\ mol^{-1} \qquad \text{Eq. S4}$$

For $^{12}C{\equiv}^{12}C$ acetylide:

$$\mu = \frac{12\ gmol^{-1} \cdot 12\ gmol^{-1}}{12\ gmol^{-1} + 12\ gmol^{-1}} = 6.0\ g\ mol^{-1} = 3.9 \cdot 10^{24}\ g \qquad \text{Eq. S5}$$

$$k_{12C\equiv 12C} = \mu(2\pi c\nu)^2 = 1.89\ \cdot 10^{11}\ cm^{-1} \qquad \text{Eq. S6}$$

For $^{13}C{\equiv}^{13}C$ acetylide:

$$\mu = \frac{13\ gmol^{-1} \cdot 13\ gmol^{-1}}{13\ gmol^{-1} + 13\ gmol^{-1}} = 6.5\ g\ mol^{-1} = 3.6 \cdot 10^{24}\ g \qquad \text{Eq. S7}$$

$$\nu_{13C\equiv 13\mathrm{C}} = \frac{1}{2\pi c}\sqrt{\frac{k}{\mu}} = 2005\ cm^{-1} \qquad \text{Eq. S8}$$

Calculated vibrational frequency shift of ν(CC)a upon $^{12}C \rightarrow {}^{13}C$ isotopic substitution:

$$\nu_{12C\equiv 12\mathrm{C}} - \nu_{13C\equiv 13\mathrm{C}} = 82\ cm^{-1} \qquad \text{Eq. S9}$$

Experimental vibrational frequency shift of ν(CC)a upon $^{12}C \rightarrow {}^{13}C$ isotopic substitution:

$$2007\ cm^{-1} - 2087\ cm^{-1} = 81\ cm^{-1} \qquad \text{Eq. S10}$$

The reduced mass analysis shows that the observed shift in vibrational frequency in the FTIR data is consistent with expected change based upon $^{12}C \rightarrow {}^{13}C$ isotopic substitution.

3.2 Spectral deconvolution

The normalised ground state FTIR spectra of complexes **1**-**4** in the 1975 – 2125 cm^{-1} region were deconvoluted by fitting of pseudo-Voigt profiles to the experimental data.

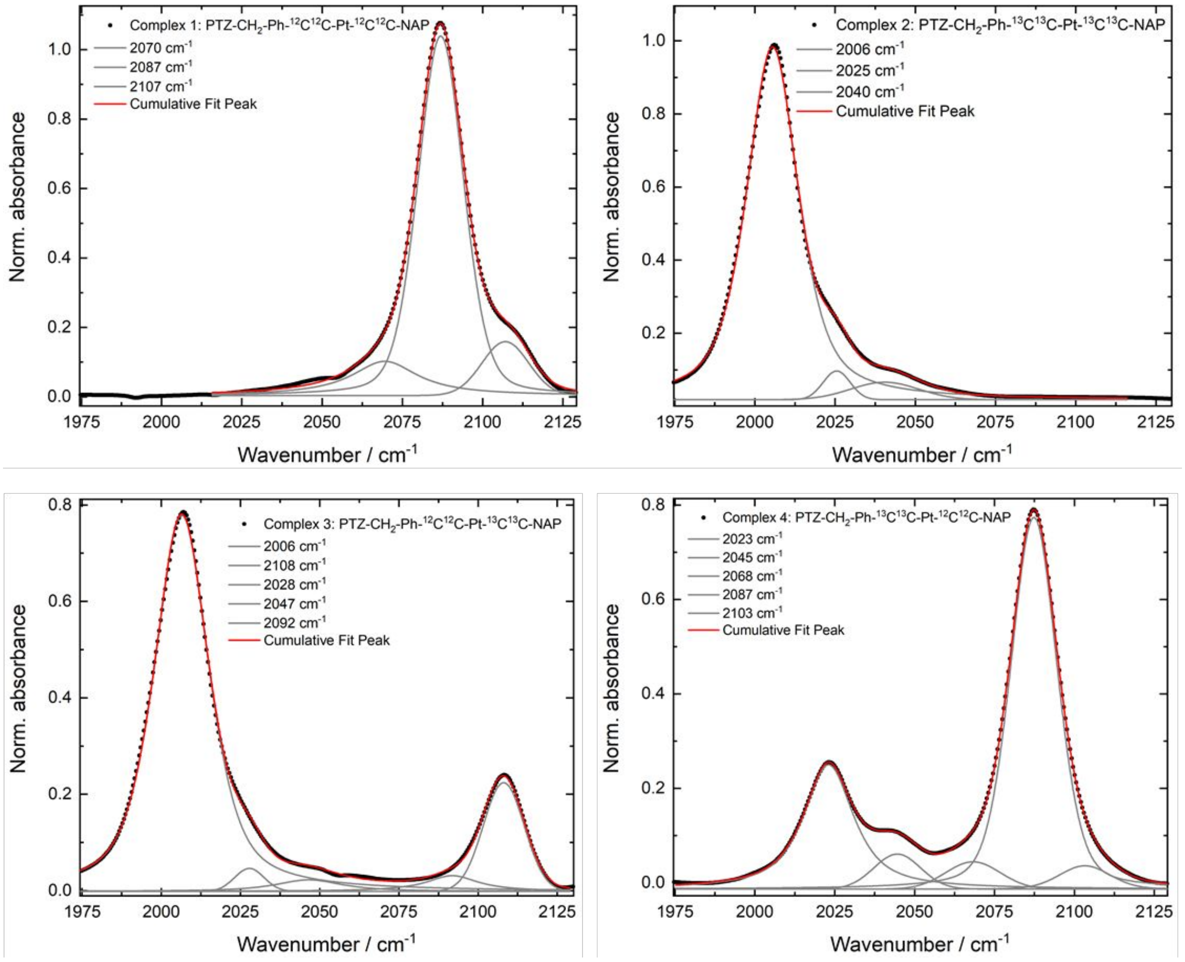

*Figure S2 – Deconvoluted ground state FTIR spectra for complexes **1 – 4** using pseudo-Voigt curves.*

3.3 Frequency scaled harmonic IR spectra

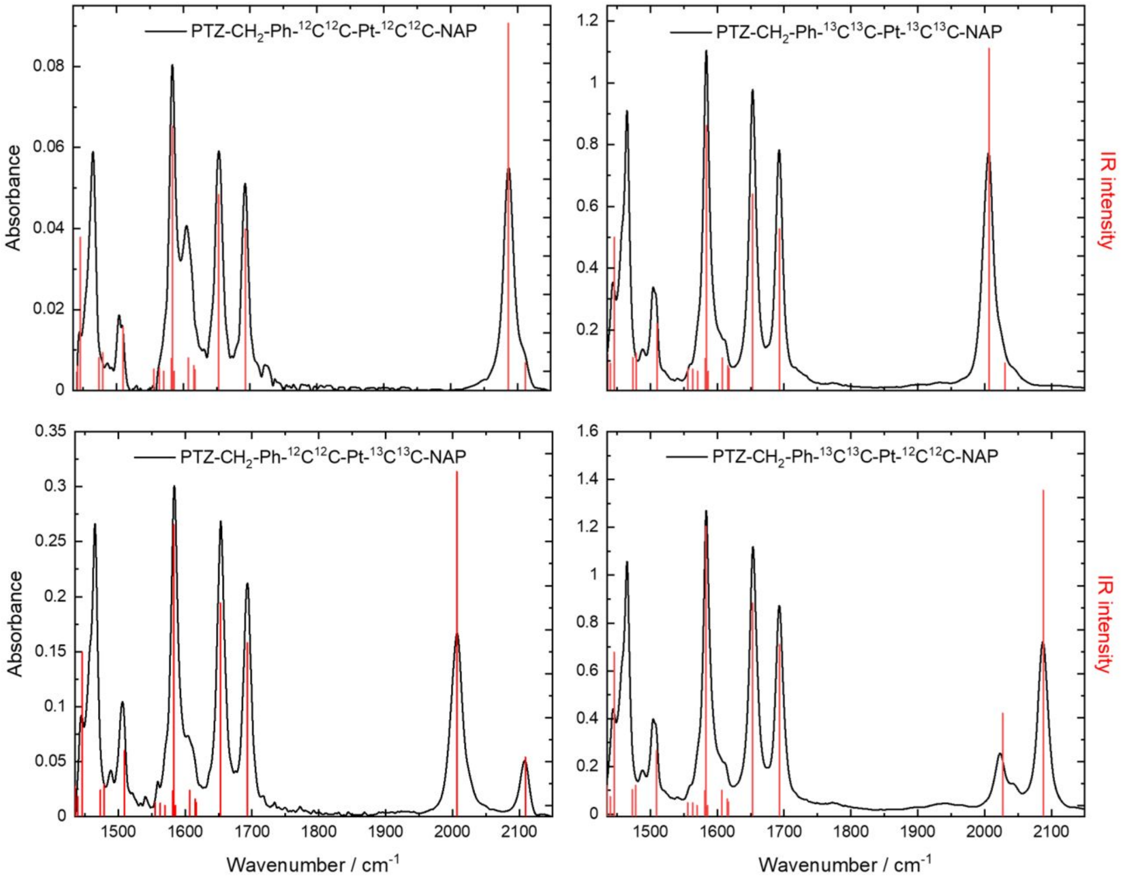

*Figure S3 – Calculated harmonic IR spectra (Red lines) obtained from the optimised electronic ground state geometries of complexes **1-4**, overlaid with the experimental IR spectra recorded in dichloromethane solution within a CaF_2 solution cell. The harmonic frequencies have been scaled to match the frequencies of the experimental data.*

*Table S1 - Harmonic scaling factors for the calculated vibrational frequencies of **1 – 4**.*

**Complex**	**Harmonic IR Frequency Scaling Factor**	
	Other Modes	*$v(CO_a)$, $v(CO_s)$*
**1**	0.955	0.935
**2**	0.955	0.935
**3**	0.955	0.935
**4**	0.955	0.935

4. Supplementary 2D-IR Spectra

The following figures represent an overview of all collected datasets that are discussed in the main text.

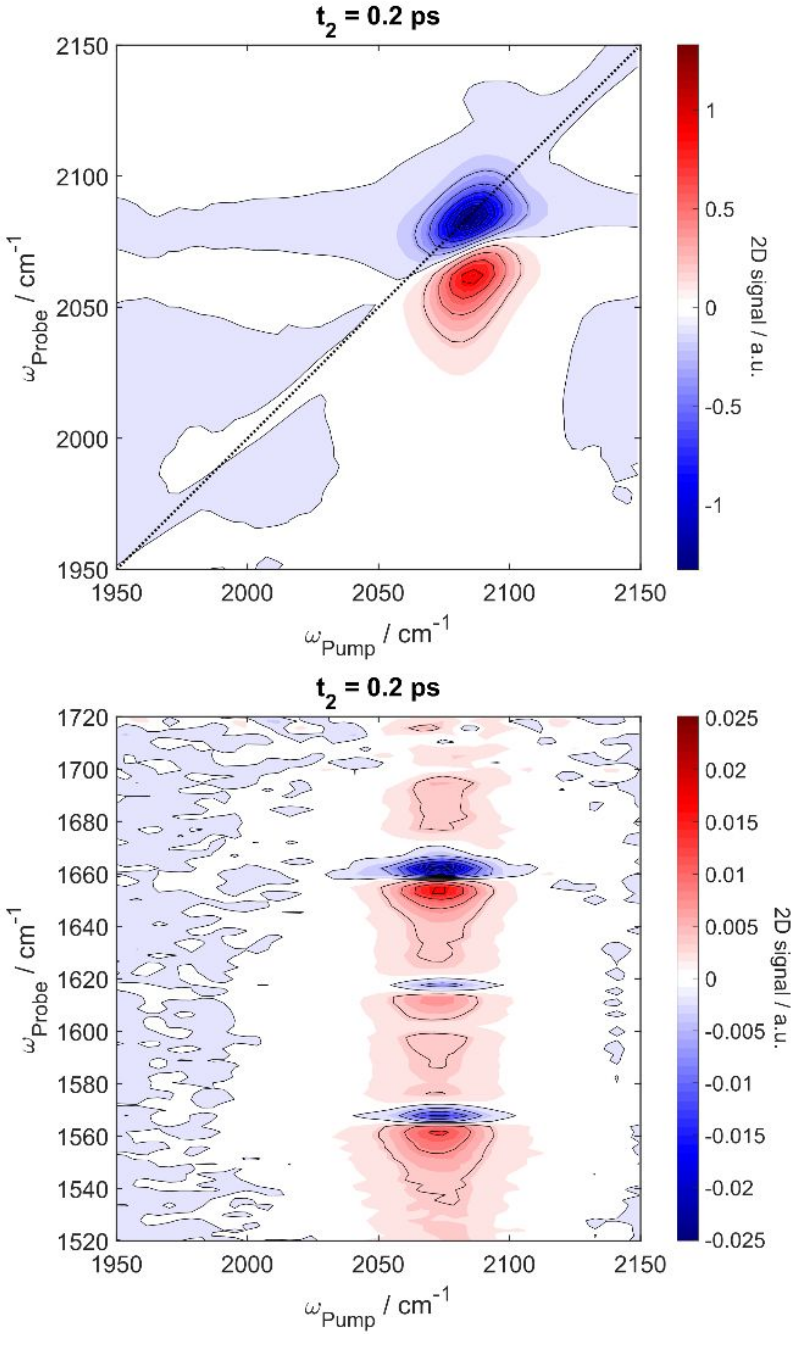

*Figure S4 – All 2D-IR spectra obtained from complex **1** following excitation in the 1950 – 2150 cm^{-1} region of the spectrum, with the pump centred at 2080 cm^{-1}.*

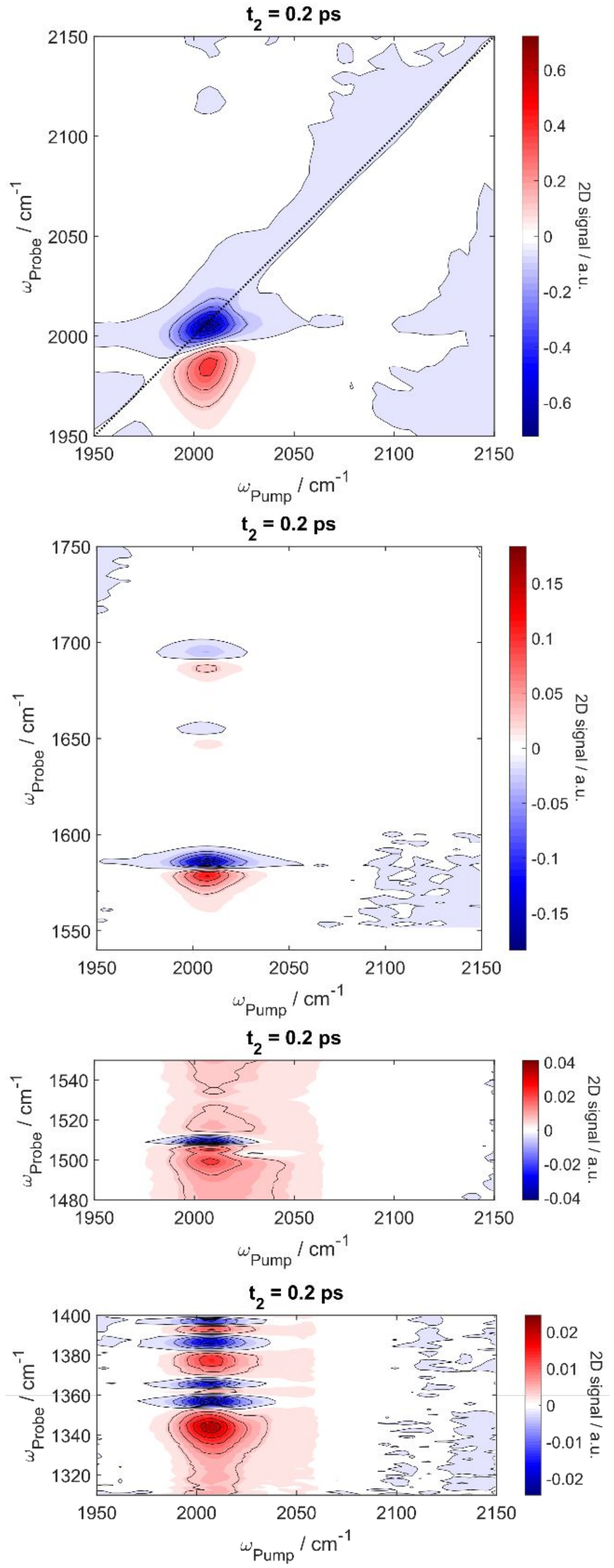

*Figure S5 – All 2D-IR spectra obtained from complex **2** following excitation in the 1950 – 2150 cm*$^{-1}$ *region of the spectrum, with the pump centred at 2020 cm*$^{-1}$*.*

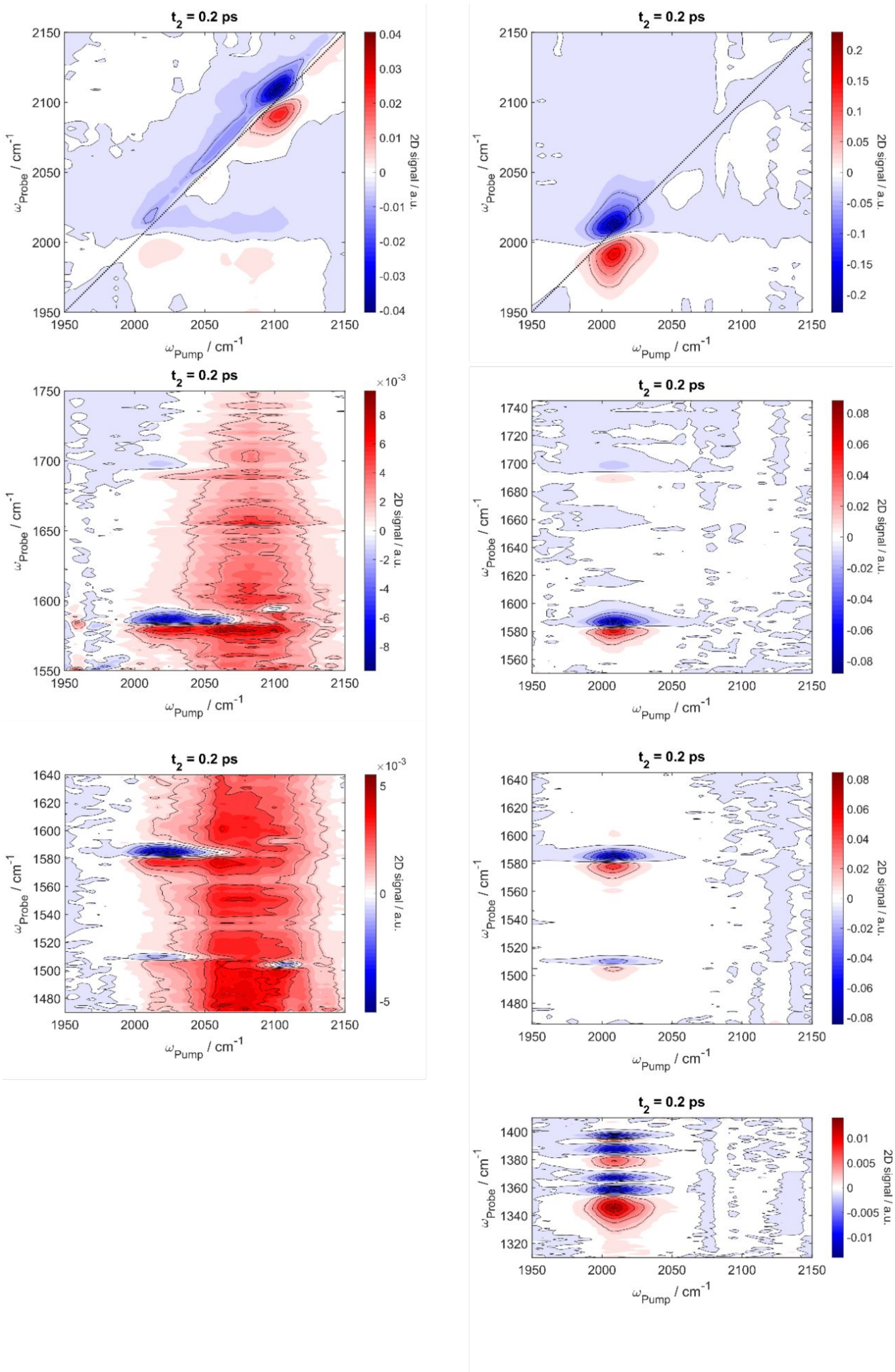

Figure S6 – All 2D-IR spectra obtained from complex ***3*** *following excitation in the 1950 – 2150 cm^{-1} region of the spectrum, with the pump centred at 2020 or 2080 cm^{-1}.*

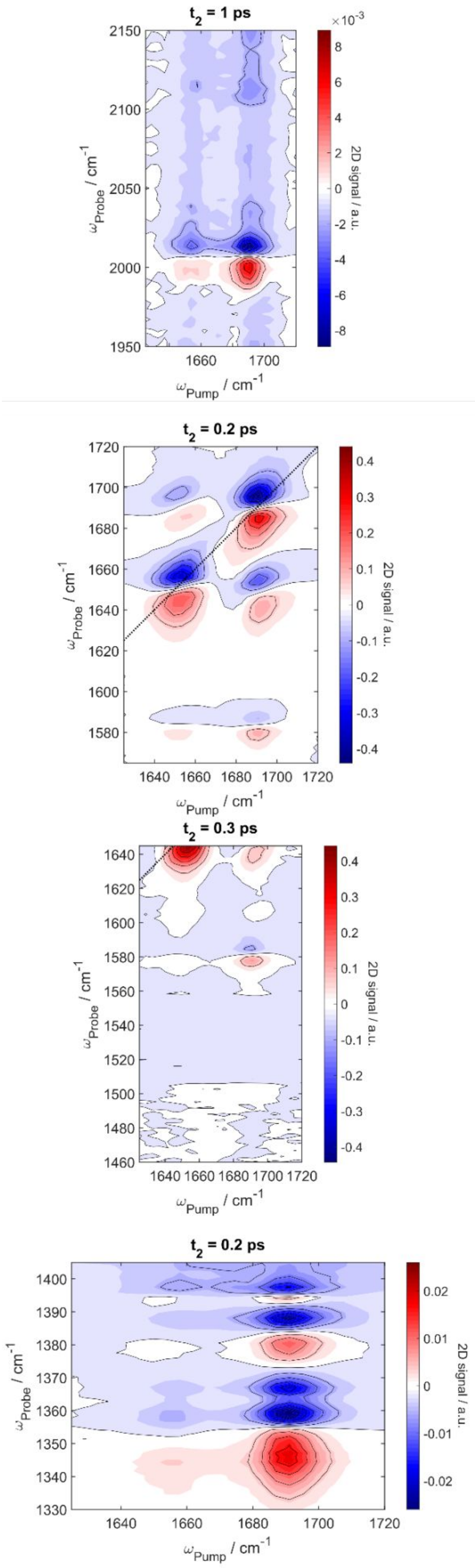

*Figure S7 – All 2D-IR spectra obtained from complex **3** following excitation in the 1620 – 1720 cm^{-1} region of the spectrum, with the pump centred at 1700 cm^{-1}.*

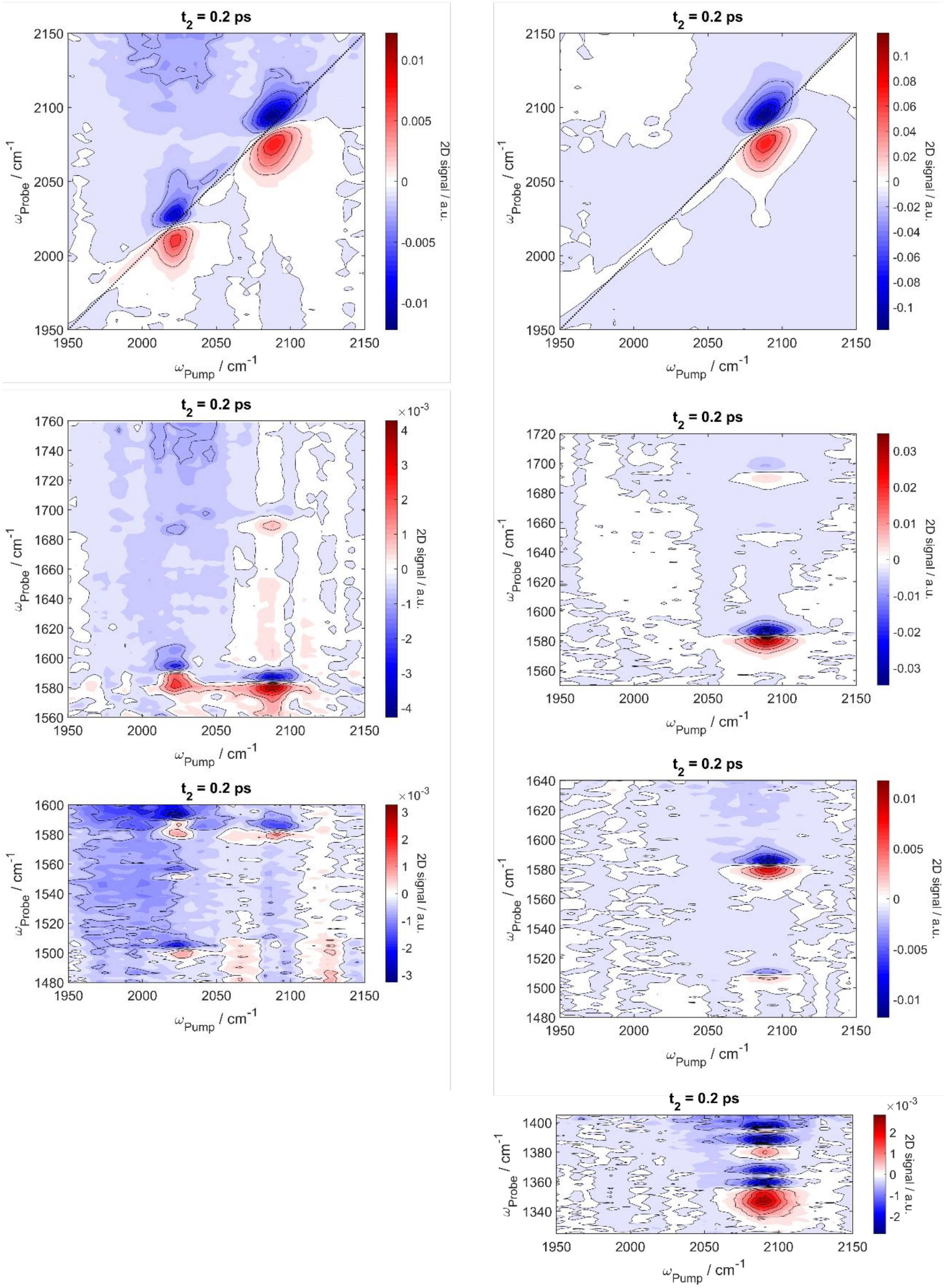

*Figure S8 – All 2D-IR spectra obtained from complex **4** following excitation in the 1950 – 2150 cm^{-1} region of the spectrum, with the pump centred at 2020 or 2080 cm^{-1}.*

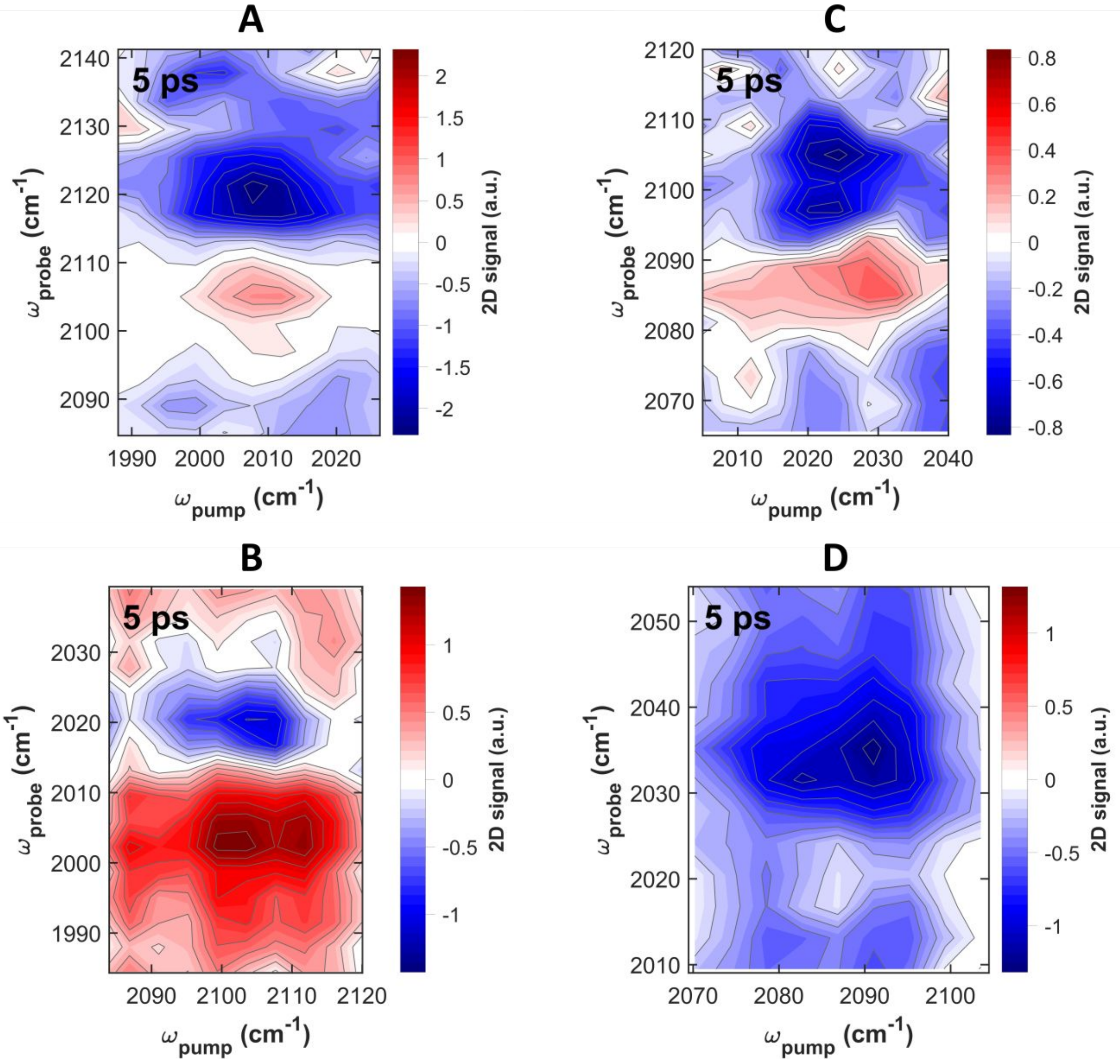

*Figure S9 – 2D-IR spectra of complexes **3** and **4**, cropped around the cross-peaks of the acetylide stretching vibrations formed by population transfer during t_2. A) Complex **3**, $\omega_{pump} \approx 2010$ cm^{-1} ($^{13}C^{13}C$), $\omega_{probe} \approx 2100$ cm^{-1} ($^{12}C^{12}C$). B) Complex **3**, $\omega_{pump} \approx 2100$ cm^{-1} ($^{12}C^{12}C$), $\omega_{probe} \approx 2010$ cm^{-1} ($^{13}C^{13}C$). C) Complex **4**, $\omega_{pump} \approx 2020$ cm^{-1} ($^{13}C^{13}C$), $\omega_{probe} \approx 2100$ cm^{-1} ($^{12}C^{12}C$). Complex **4**, $\omega_{pump} \approx 2090$ cm^{-1} ($^{12}C^{12}C$), $\omega_{probe} \approx 2020$ cm^{-1} ($^{13}C^{13}C$).*

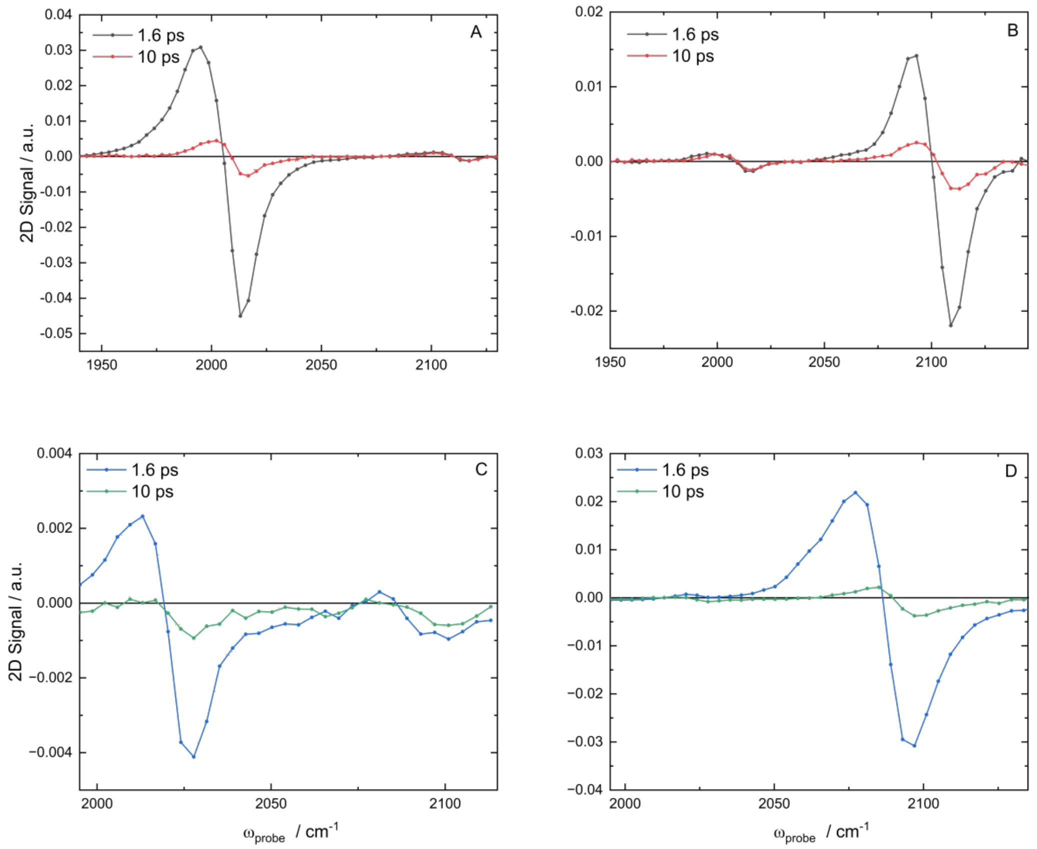

*Figure S10 – Cross-sections of the 2D-IR spectra of complexes **3** and **4**, taken at the peak maxima of the acetylide stretching vibrations along the ω_{pump} axis. A) Complex **3**, $\omega_{pump} \approx 2012\ cm^{-1}$ ($^{13}C^{13}C$. B) Complex **3**, $\omega_{pump} \approx 2104\ cm^{-1}$ ($^{12}C^{12}C$). C) Complex **4**, $\omega_{pump} = 2024\ cm^{-1}$ ($^{13}C^{13}C$). Complex **4**, $\omega_{pump} = 2091\ cm^{-1}$ ($^{12}C^{12}C$).*

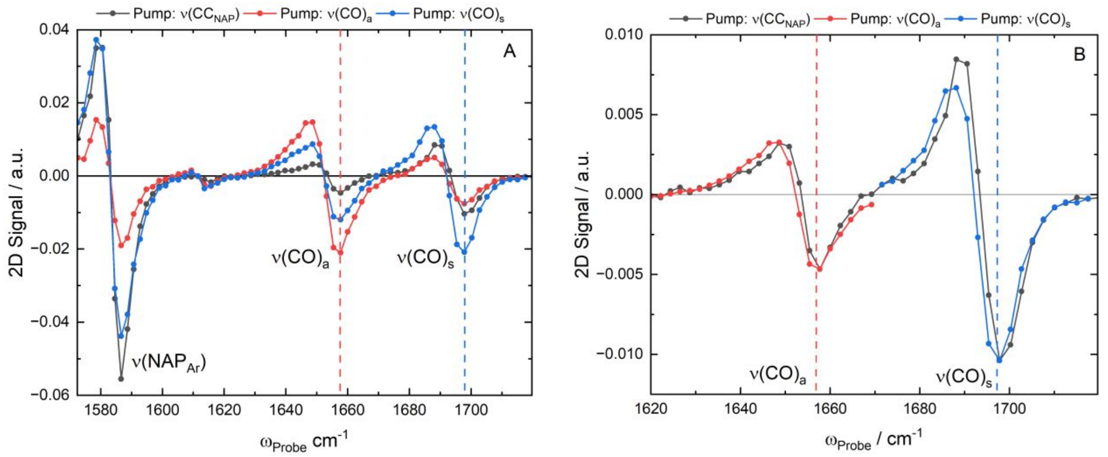

*Figure S11 – A) Cross-sections of the 2D-IR spectra of complex **3** (as a representative example), comparing the lineshapes of the ν(NAP_{Ar}), ν$(CO)_a$, and ν$(CO)_s$ peak pairs following excitation of the ν(CC_{NAP}), ν$(CO)_a$ or ν$(CO)_s$ modes, as stated on the graph legends. The data in graph B have been rescaled such that the bleach intensity of the diagonal cross-sections is the same as the off-diagonal cross-sections for lineshape comparison purposes.*

5. Vibrational coupling between ν(CC)$_a$ and ν(CC)$_s$

Complex 1

The dynamics of the ν(CC)$_a$ diagonal and off-diagonal signals were studied by taking cross-sections of the 2D-IR spectra of **1** at pump axis intersects of 2086 cm^{-1}, and 2120 cm^{-1}, as well as at a probe intersect of 2109 cm^{-1}. The corresponding diagonal (ν$_{1\text{-}2}$(CC)$_a$) and off-diagonal ([ν$_{0\text{-}1}$(CC)$_a$]*) excited state absorption bands were obscured by the much stronger ν$_{0\text{-}1}$(CC)$_a$ bleach. Excitation of the ν(CC)$_a$ mode resulted in the grow in of three observable bands along the 2086 cm^{-1} excitation cross section (Figure S12c). These grew in with IRF convoluted time constants. However, the dynamics of the ν(CC)$_a$/ν(CC)$_s$ off-diagonal bleach (2074, 2109 cm^{-1}) signal were different, where the IVR rate was faster, and the VET rate was slower compared to the ν(CC)$_a$ diagonal band (Figure S12b,d). The differences in IVR and VET rate for these modes is possibly a result of the fact that the ν(CC)$_a$ mode is primarily localised on the NAP-CC group, whilst the ν(CC)$_s$ mode is primarily localised on the PTZ-CC group, as shown by the calculations in the main text.

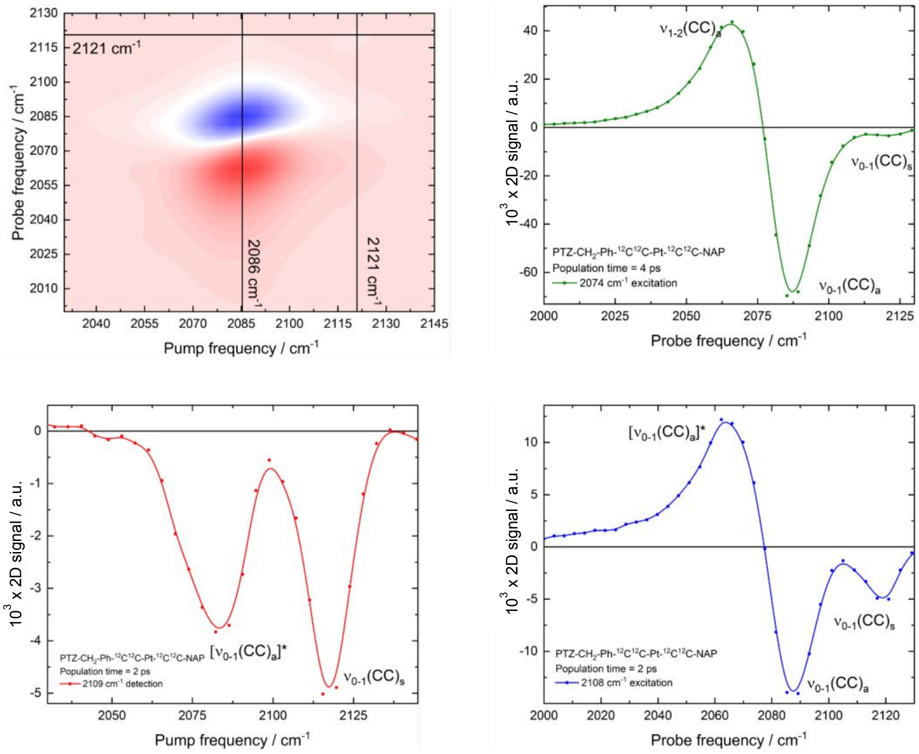

*Figure S12 – Cross section 2D-IR spectra of **1** taken at pump axis intersects of 2074 and 2109 cm^{-1}(c, d) and a probe axis intersect of 2109 (b) cm^{-1} as shown on the 2D contour map (a).*

Complex 2

Off-diagonal signals in the acetylide region for **2** were treated in the same manner as described for **1**. It was not possible to clearly distinguish the ν(CC)$_a$/ν(CC)$_s$ cross peaks from the ν(CC)$_a$ diagonal signal due to the smaller frequency separation of the two fundamental vibrations (Figure S12). In addition, the ν(CC)$_s$ bleach sits within the band envelope of the ν(CC)$_a$ bleach. These peak overlaps prevented accurate modelling of the growth and decay constants of the bands in this region associated with the ν(CC)$_s$ mode. Therefore, few conclusions could be drawn about the dynamics of vibrational energy transfer between the ν(CC)$_a$ and ν(CC)$_s$ modes in this complex.

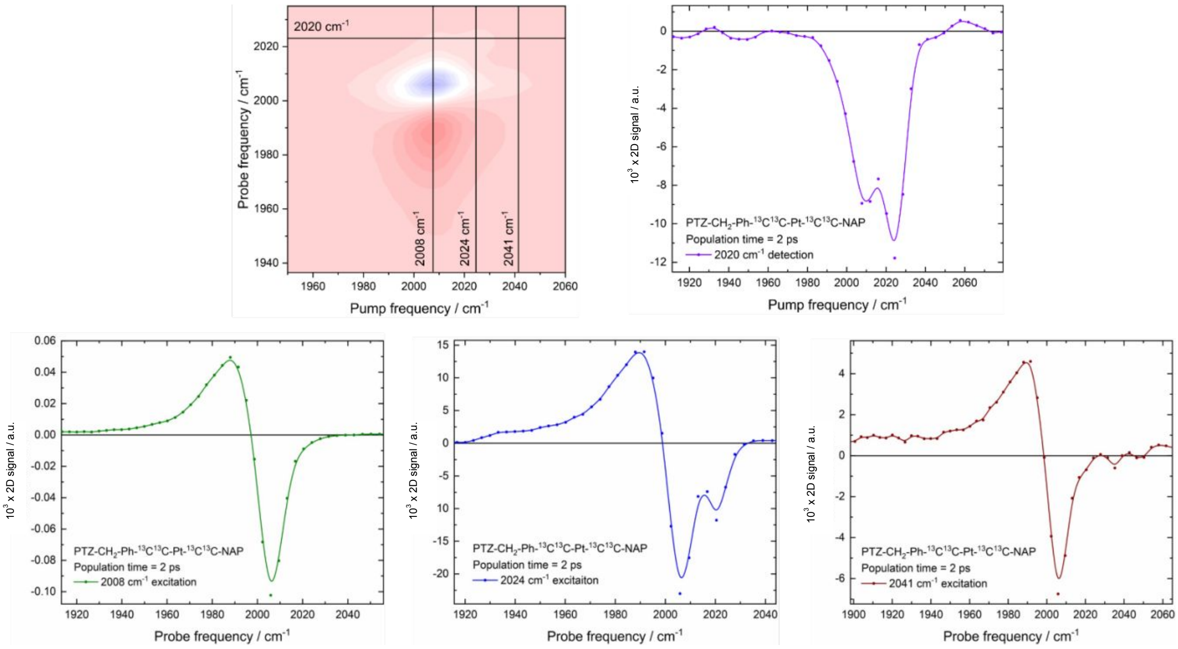

*Figure S13 – Cross section 2D-IR spectra of **2** taken at pump axis intersects of 2008, 2024, and 2041 cm^{-1}(c, d, e) and a probe axis intersect of 2020 (b) cm^{-1} as shown on the 2D contour map (a).*

6. Kinetics

Kinetic time constants were obtained from fitting of the experimental kinetic traces with a biexponential function. The resulting τ_1 and τ_2 values represent the biexponential decay rates of the diagonal peaks, or the growth and decay behaviour of the cross-peaks. For each peak pair in Table S2, the amplitude of the ground state bleach was subtracted from the excited state absorption band at each time delay to remove baseline fluctuations from the kinetic trace and improve the signal: noise ratio prior to fitting.

Table S2 – Time constants and exponential amplitudes obtained from exponential fitting of the 2D-IR kinetic traces obtained for the specified peak pair. The uncertainties provided here originate from the exponential fitting procedure and do not take into account the 200 fs instrument response of the 2DIR spectrometer.

Complex	Pump	Probe	A_1	τ_1 / ps	A_2	τ_2 / ps
**1**	ν(CC)$_a$	ν(CC)$_a$	2.3 ± 0.031 (95%)	0.91 ± 0.013	0.20 ± 0.032 (5%)	3.7 ± 0.51
**1**	ν(CC)$_a$	ν(CO)$_a$	-0.002 ± 5x10^{-4} (16%)	1.9 ± 0.48	0.01 ± 3x10^{-4} (83%)	13 ± 1.2
**1**	ν(CC)$_a$	ν(CO)$_s$	-0.018 ± 0.002 (23%)	1.4 ± 0.20	0.06 ± 0.001 (77%)	9.8 ± 0.60
**2**	ν(^{13}CC)$_a$	ν(^{13}CC)$_a$	0.88 ± 0.0048 (91%)	0.63 ± 0.0071	0.089 ± 0.0029 (9%)	7.7 ± 0.75
**2**	ν(^{13}CC)$_a$	ν(CO)$_a$	-0.014 ± 5x10^{-4} (30%)	0.55 ± 0.034	0.032 ± 2x10^{-4} (70%)	9.2 ± 0.23
**2**	ν(^{13}CC)$_a$	ν(CO)$_s$	-0.02 ± 6x10^{-4} (22%)	0.45 ± 0.022	0.073 ± 2x10^{-4} (78%)	8.5 ± 0.10
**3**	ν(NAP-^{13}CC)	ν(NAP-^{13}CC)	0.97 ± 0.0099 (68%)	0.63 ± 0.011	0.45 ± 0.010 (32%)	3.9 ± 0.31
**3**	ν(NAP-^{13}CC)	ν(CC-PTZ)	-0.81 ± 0.35 (40%)	2.21 ± 1.0	1.2 ± 0.23 (60%)	13 ± 7.7
**3**	ν(CC-PTZ)	ν(CC-PTZ)	0.49 ± 0.019 (41%)	0.21 ± 0.012	0.71 ± 0.0045 (59%)	4.5 ± 0.061
**3**	ν(CC-PTZ)	ν(NAP-^{13}CC)	/	/	0.84 ± 0.015	21 ± 1.30
**3**	ν(NAP-^{13}CC)	ν(CO)$_a$	-0.0057 ± 0.0004 (1%)	0.40 ± 0.051 (99%)	0.010 ± 2.0x10^{-4}	12 ± 0.84
**3**	ν(NAP-^{13}CC)	ν(CO)$_s$	-0.0096 ± 2.1x10^{-4} (1%)	0.70 ± 0.043	0.030 ± 1.6x10^{-4} (99%)	10 ± 0.23
**3**	ν(CC-PTZ)	ν(CO)$_a$	-0.0011 ± 3.6x10^{4} (18%)	2.6 ± 1.34	0.005 ± 0.042 (82%)	137 ± 1334**

**3**	ν(CC-PTZ)	ν(CO)$_s$	-0.0016 ± 3.5x10^{4} (32%)	2.6 ± 0.62	0.0034 ± 5.4x10^{4} (68%)	25 ± 13
**4**	ν(NAP-CC)	ν(NAP-CC)	0.19 ± 0.0026 (91%)	0.95 ± 0.022	0.018 ± 0.0024 (9%)	6.5 ± 1.5
**4**	ν(NAP-CC)	ν(^{13}CC-PTZ)	-0.0033 ± 1.1x10^{-4} (65%)	1.4 ± 0.11	0.0018 ± 6.4x10^{-4} (35%)	31 ± 21
**4**	ν(^{13}CC-PTZ)	ν(^{13}CC-PTZ)	0.010 ± 4.4x10^{-5} (50%)	0.48 ± 0.042	0.010 ± 5.9x10^{-4} (50%)	2.4 ± 0.12
**4**	ν(^{13}CC-PTZ)	ν(NAP-CC)	-0.0015 ± 0.0014 (42%)	2.7 ± 1.8	0.0021 ± 0.0013 (58%)	9.3 ± 6.0
**4**	ν(NAP-CC)	ν(CO)$_a$	-0.0034 ± 8.1x10^{-5} (31%)	0.77 ± 0.046	0.0077 ± 6.0x10^{-5} (69%)	11 ± 0.36
**4**	ν(NAP-CC)	ν(CO)$_s$	-0.0076 ± 1.4x10^{-4} (31%)	1.0 ± 0.036	0.017 ± 1.0x10^{-4} (69%)	9.8 ± 0.20
**4**	ν(^{13}CC-PTZ)	ν(CO)$_a$	-5.9x10^{-4} ± 1.32x10^{-4}	1.3 ± 0.61	4.7x10^{-4} ± 1.1x10^{-4}	10 ± 6.6
**4**	ν(^{13}CC-PTZ)	ν(CO)$_s$	-0.0016 ± 1.9x10^{-4} (53%)	1.8 ± 0.25	0.0014 ± 1.79x10^{-4} (47%)	8.8 ± 1.72

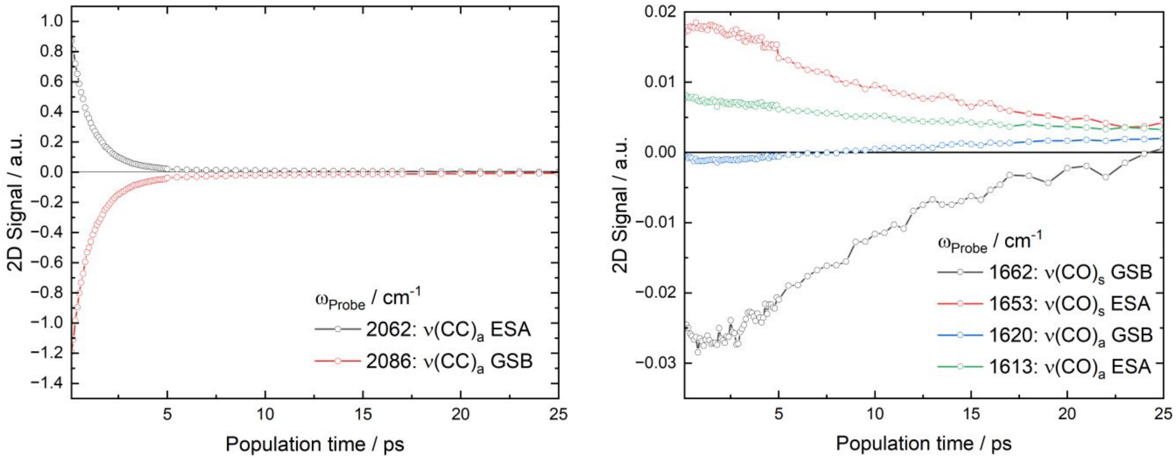

*Figure S14 – Kinetic traces obtained from Complex **1** following IR excitation of the $\nu(CC)_a$ mode at 2086 cm^{-1}, and detection at the probe frequencies stated on the graph legends.*

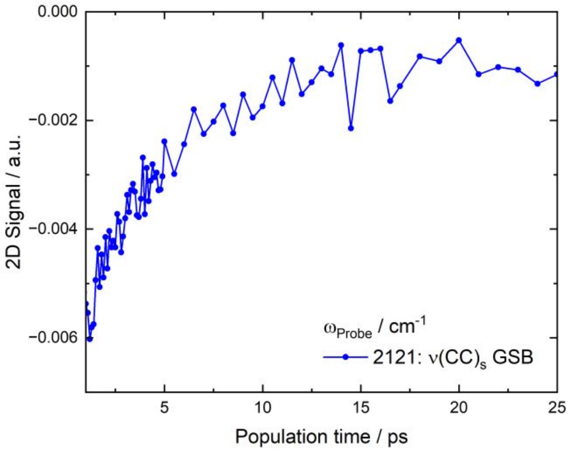

*Figure S15 – Kinetic trace obtained from complex **1** for the diagonal $\nu(CC)_s$ ground state bleach at 2121 cm^{-1}.*

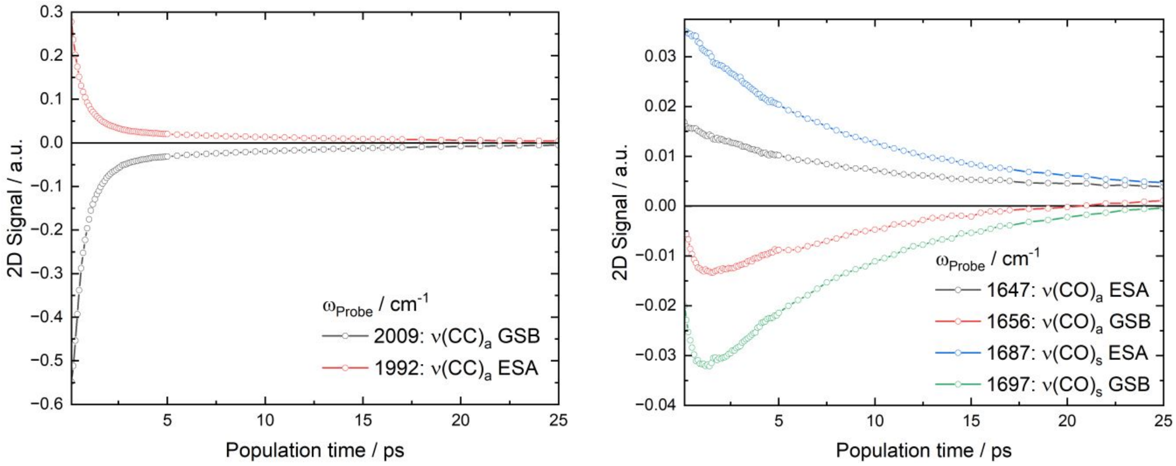

*Figure S16 – Kinetic traces obtained from Complex **2** following IR excitation of the $\nu(CC)_a$ mode at 2008 cm^{-1}, and detection at the probe frequencies stated on the graph legends.*

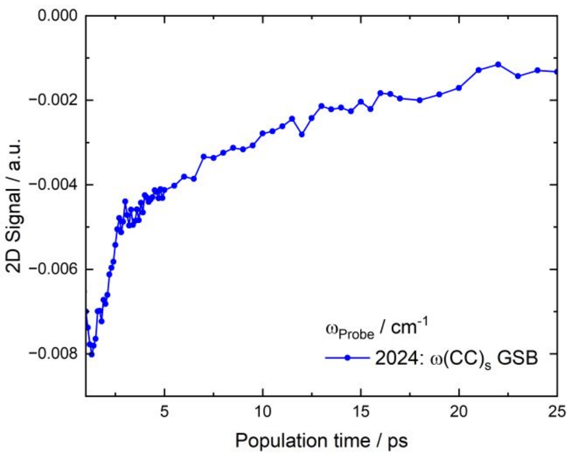

*Figure S17 – Kinetic trace obtained from complex **1** for the diagonal ν(CC)$_s$ ground state bleach at 2024 cm^{-1}.*

7. Spectral diffusion

7.1 Theoretical background

The instantaneous fluctuations of the vibrational frequencies with respect to the time average are related to the observed lineshapes in the 2D-IR spectra. Spectral diffusion occurs over the timescale in which these fluctuations take place and can be evaluated by the *frequency-frequency correlation function* (FFCF), $C(t)$, which is defined as $C(t) = \langle \delta\omega(t)\delta\omega(0) \rangle$. The Kubo model of the FFCF contains an instantaneous decay component that accounts for homogeneous broadening, as well as one (or more) exponential decay components that model inhomogeneous contributions.[18]

$$C(t) = \frac{\delta(t)}{T_2} + \sum_i \Delta_i^2 e^{-\frac{t}{\tau_i}} \qquad \text{Eq. S11}$$

The kinetics of spectral diffusion are related to the decay of the FFCF and can be extracted from 2D-IR spectra through analysis of the 2D lineshapes. Most analysis methods yield the normalised FFCF, $C'(t)$.

$$\text{Norm. FFCF} = C'(t_2) = \frac{C(t_2)}{C(0)} = \frac{\langle \delta\omega(t_2)\delta\omega(0) \rangle}{\langle \delta\omega(0)^2 \rangle} \qquad \text{Eq. S12}$$

The full FFCF can be recovered by simultaneous fitting of the 2D-IR and linear (FTIR) absorption spectra,[19] but its dynamics are independent of the normalisation factor. Several methods have been shown to extract the spectral diffusion rates from 2D-IR spectra, including the centre line slope (CLS),[20–22] the correlation coefficient of a 2D Gaussian function (which are directly equivalent to the normalised FFCF),[23] and the inhomogeneity index (I.I.),[24] which is defined as:

$$I.I.(t_2) = \frac{A_R(t_2) - A_{NR}(t_2)}{A_R(t_2) + A_{NR}(t_2)} \qquad \text{Eq. S13}$$

Where A_R and A_{NR} are the amplitudes of the time dependent rephasing and non-rephasing spectra, respectively. The I.I. is related to the normalised FFCF by:

$$C'(t_2) = \sin\left[\frac{\pi}{2} \times I.I.(t_2)\right] \qquad \text{Eq. S14}$$

7.2 Acetylide modes

The selected method for measurement of the spectral diffusion rate was Centre Line Slope analysis, where the change in tilt angle of the ν_{1-2} and ν_{0-1} bands is plotted over time, this angle approaches zero as spectral diffusion occurs. This analysis was done with software developed at the UKRI STFC Central Laser Facility by Sebastian Gorgon and is available online (https://github.com/sebgorgon/2D-IR-spectral-diffusion). The 2D-IR spectra were fit by two 2D-Gaussian peaks to obtain the gradient of the centre line slope at each t_2 delay. The resulting exponential data was then fit by an exponential function to yield the rates of spectral diffusion. The centre frequencies of the two peaks were also used to obtain the anharmonic shift of each peak pair.

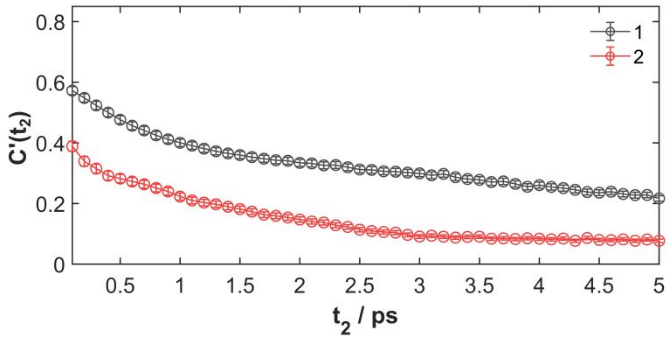

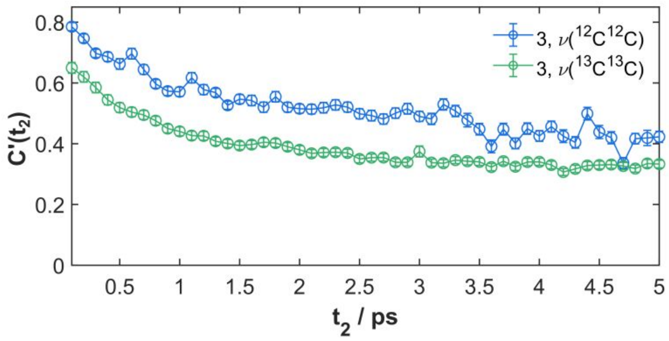

*Figure S18 – Estimated frequency-frequency correlation functions for the acetylide vibrational modes of **1** – **3**, calculated using the centre line slope method by fitting of 2D gaussian peaks to the 2D-IR data at each population time. The displayed error bars are calculated from the uncertainty in the fit of each 2D gaussian.*

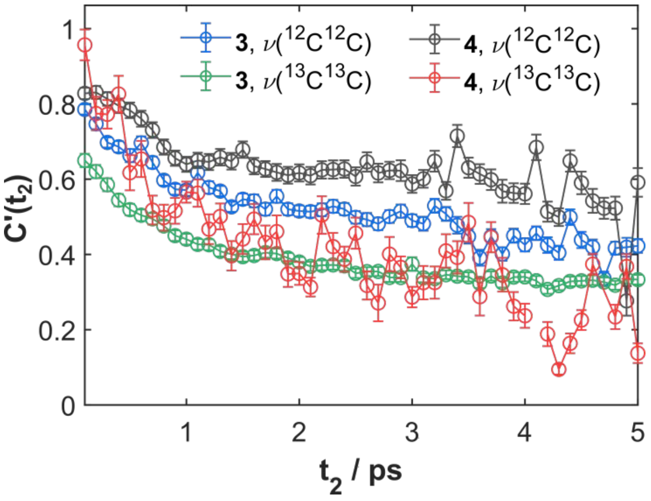

*Figure S19 – Estimated frequency-frequency correlation functions for the acetylide vibrational modes of **3** and **4**, calculated using the centre line slope method by fitting of 2D gaussian peaks to the 2D-IR data at each population time. The displayed error bars are calculated from the uncertainty in the fit of each 2D gaussian.*

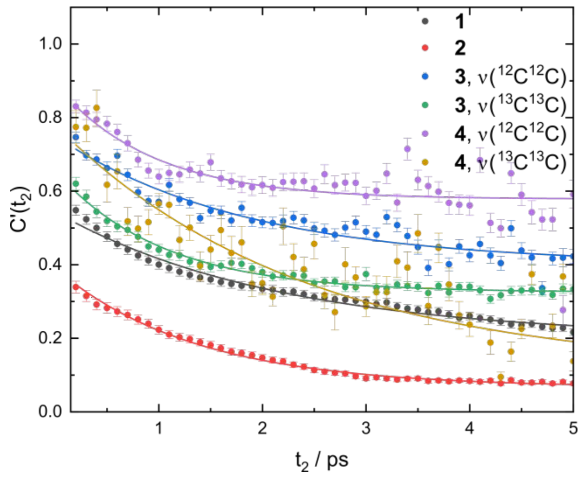

*Figure S20 – Estimated frequency-frequency correlation functions for the acetylide vibrational modes of **1** – **3**, calculated using the centre line slope method by fitting of 2D gaussian peaks to the 2D-IR data at each population time (dots). The displayed error bars are calculated from the uncertainty in the fit of each 2D gaussian. The solid lines are exponential fits of each FFCF, the fit parameters are provided in Table S3.*

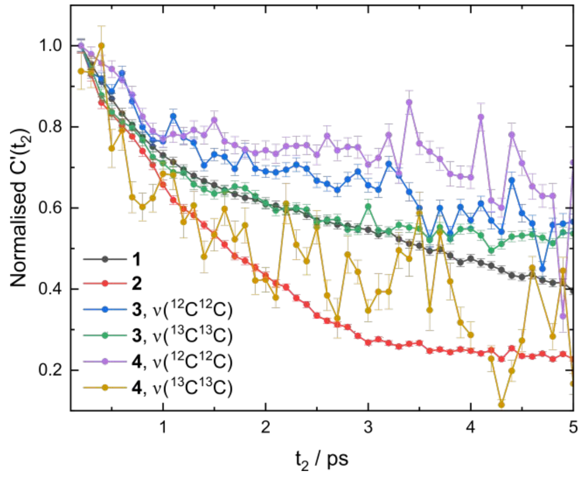

*Figure S21 – Normalised estimated frequency-frequency correlation functions for the acetylide vibrational modes of **1** – **3**, calculated using the centre line slope method by fitting of 2D gaussian peaks to the 2D-IR data*

at each population time (dots). The displayed error bars are calculated from the uncertainty in the fit of each 2D gaussian. The FFCFs are normalised to the value at t_2 = 200 fs.

Table S3 – Exponential fitting parameters obtained from fitting of the Norm. FFCFs obtained by 2D-Gaussian fitting of the diagonal peaks in the 2D-IR spectra.

Complex	Mode	FFCF Amplitude	FFCF Time constant / ps
**1**	$\nu(CC)_a$	0.34 ± 0.0091	2.2 ± 0.17
**2**	$\nu(^{13}C^{13}C)_a$	0.32 ± 0.0067	1.4 ± 0.048
**3**	$\nu(NAP\text{-}^{13}C^{13}C)$	0.33 ± 0.011	1.0 ± 0.056
**3**	ν(CC-PTZ)	0.34 ± 0.018	1.8 ± 0.31
**4**	ν(NAP-CC)	0.31 ± 0.033	0.93 ± 0.20
**4**	$\nu(PTZ\text{-}^{13}C^{13}C)$	0.67 ± 0.065	2.4 ± 0.85

7.3 Carbonyl modes

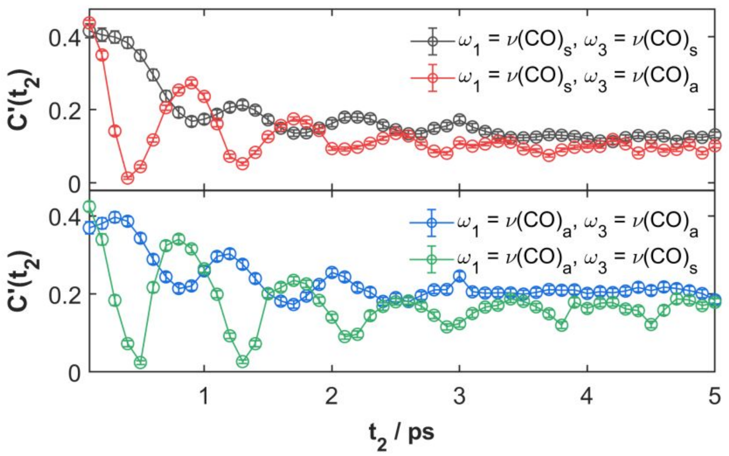

*Figure S22 – Estimated frequency-frequency correlation functions for the carbonyl vibrational modes of **3**, calculated using the centre line slope method by fitting of 2D gaussian peaks to the 2D-IR data at each population time. The displayed error bars are calculated from the uncertainty in the fit of each 2D gaussian.*

An exponential decay multiplied with a damped cosine function was used to fit the oscillating FFCF and obtain the rate of spectral diffusion (Eq. S1). Here A is the exponential amplitude, t_2 is the population delay, τ is the exponential lifetime, ω is the oscillation period (in picoseconds), ϕ is the phase offset of the oscillation, and y_0 is a static offset. The obtained fit parameters are given in Table S2. The oscillation period (*ca.* 30 cm^{-1}) obtained from these fits does not match the frequency separation between the $\nu(CO)_s$ and $\nu(CO)_a$ modes. However, fitting of a single exponential function to the oscillating FFCF, followed by Fourier transformation of the oscillating residual signal does yield the expected oscillation frequency of 40 cm^{-1} (Figure S24). This discrepancy is attributable to greater uncertainty in the oscillation parameters obtained from the convolved exponential and damped cosine fitting data.

$$A_1 e^{-\frac{t_2}{\tau_1}} + A_2 e^{-\frac{t_2}{\tau_2}} \times \cos(2\pi\omega t_2 + \phi) + y_0 \quad \text{Eq. S15}$$

*Table S4 – Fitting parameters obtained by least-squares minimisation of Eq. S1 to the oscillating FFCFs obtained from centre line slope analysis of the ν(CO) peak pairs obtained from complex **3**.*

	ν(CO)$_a$ Pump ν(CO)$_a$ Probe	ν(CO)$_a$ Pump ν(CO)$_s$ Probe	ν(CO)$_s$ Pump ν(CO)$_s$ Probe	ν(CO)$_s$ Pump ν(CO)$_a$ Probe
A_1	0.20 ± 0.016	0.25 ±0.069	0.32 ± 0.024	0.16 ± 0.017
τ_1 / ps (FFCF decay)	0.72 ± 0.071	0.45 ± 0.11	0.78 ± 0.064	1.1 ± 0.16
A_2	-0.091 ± 0.010	0.31 ± 0.029	-0.10 ± 0.020	0.30 ± 0.02
τ_2 / ps (Oscillation dephasing)	1.9 ± 0.26	1.4 ± 0.14	1.4 ± 0.26	0.97 ± 0.066
ω / ps	1.2 ± 0.010	1.2 ± 0.013	1.1 ± 0.021	1.2 ± 0.011
ϕ	0.42 ± 0.099	-0.18 ± 0.10	0.11 ± 0.18	-0.33 ± 0.069
y_0	0.20 ± 0.002	0.16 ± 0.0043	0.13 ± 0.003	0.092 ± 0.0038

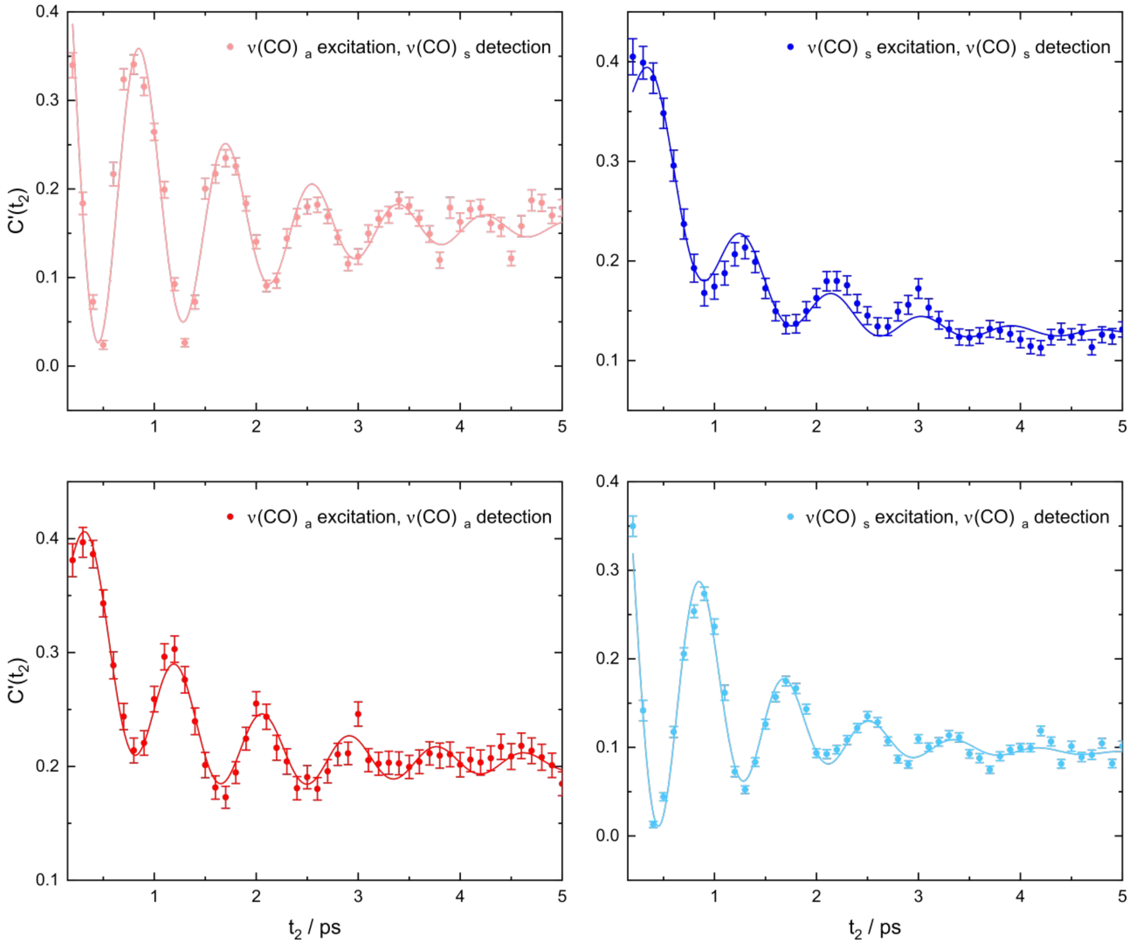

*Figure S23 – Estimated frequency-frequency correlation functions for the carbonyl vibrational modes of **4**, calculated using the centre line slope method by fitting of 2D gaussian peaks to the 2D-IR data at each population time. The solid lines are fit curves produced using least squares minimisation of Eq. S1.*

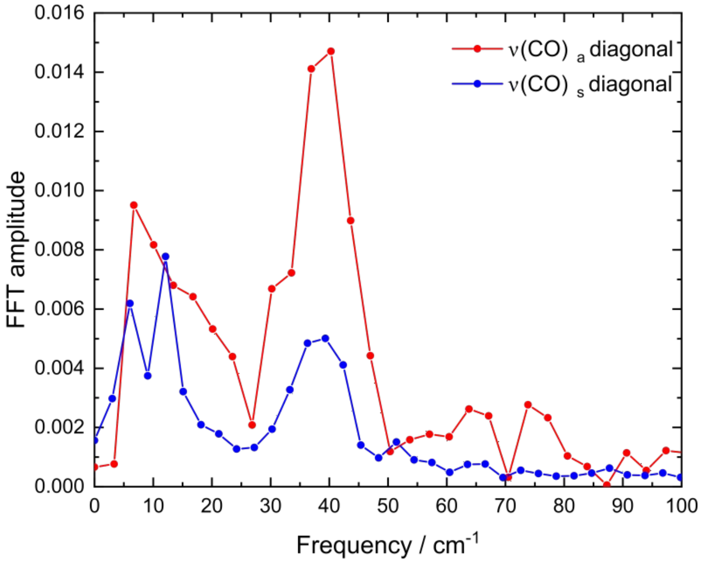

Figure S24 - Oscillation spectrum obtained by Fourier transformation of the oscillating residual data obtained from fitting of the FFCFs obtained by centre line slope analysis of the diagonal ν(CO) peak pairs in complex 3.

8. Dynamic Anharmonicities

8.1 Fitting of Voigt profiles

The data shown in Figure 7 of the main text are deconvolved with Voigt profiles. The Voigt profile [$V(x;\sigma,\gamma)$], centred at a given frequency (x') is defined by the convolution of a Gaussian [$G(x;\sigma)$] and a Lorentzian profile [$L(x;\gamma)$]:

$$V(x;\sigma,\gamma) \equiv \int_{-\infty}^{+\infty} G(x';\sigma)L(x-x';\gamma)dx' \quad \text{Eq. S16}$$

It can be shown that this integral can be evaluated as:

$$V(x;\sigma,\gamma) = \frac{\Re[\omega(z)]}{\sigma\sqrt{2\pi}} \quad \text{Eq. S17}$$

$$z = \frac{x+i\gamma}{\sigma\sqrt{2}} \quad \text{Eq. S18}$$

Where $\Re[\omega(z)]$ refers to the real part of the Faddeeva function evaluated at z. For a Voigt profile centred about x_0, one can substitute x for $x - x_0$. The fitting routine was implemented in Python (v3.12.0) using the *wofz* function and the *curve_fit* module from the SciPy library (v.1.11.4).[25] The deconvolution shown in Figure 7 in the main text was done by first evaluating the early time trace and obtaining the w_0, σ, and γ values of the GSB and ESA. The late time trace was then fit by fixing w_0 and allowing the other parameters to vary freely. In the limiting case where γ or σ are equal to zero then $V(x;\sigma,\gamma)$ simplifies to a Lorentzian peak [$L(x;\gamma)$] or Gaussian peak [$G(x';\sigma)$], respectively.

8.2 Comparing anharmonicities from 2D Gaussian fitting and Voigt fitting

It should be noted that the absolute anharmonicities and peak shifts obtained from Voigt fitting of the slices are different than those obtained from 2D Gaussian fitting. Since the 2D Gaussian fits take into account more than one pump frequency at the same time, we consider them a more reliable indicator of the anharmonicity, even though there might be a slight overestimation of the anharmonicities due to the largely homogeneous character of the vibrational transitions. Nevertheless, both fitting methods remain valid for the interpretation of our data and do not change our conclusions regarding the dynamic anharmonic shift and the relative magnitudes of the experimental anharmonicities and their comparison with those obtained from DFT calculations.

8.3 Dynamic anharmonicity change data for all complexes

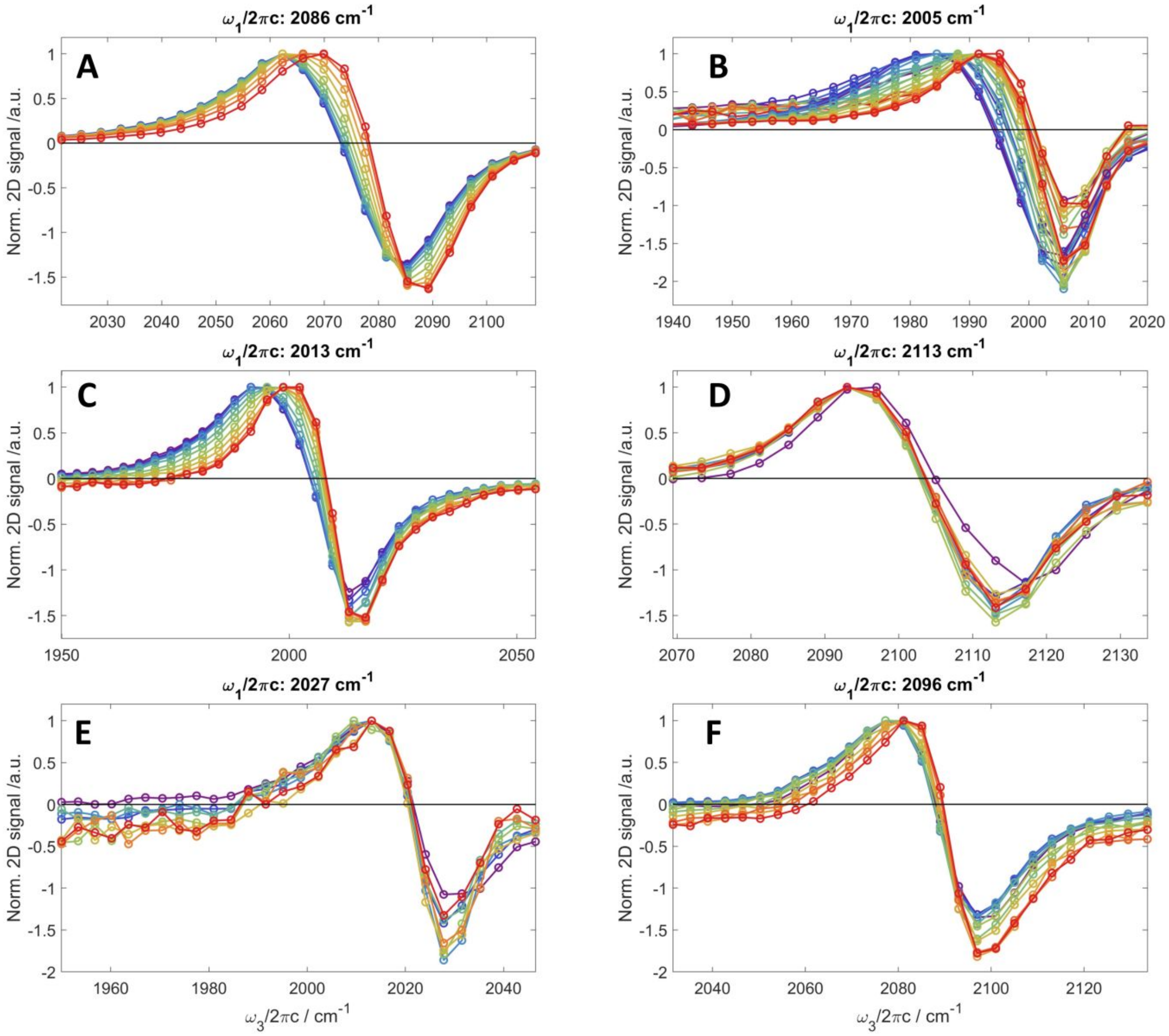

*Figure S25 – Normalised cross-sections of the 2D-IR spectra along the probe axis, intersecting the pump frequency maximum of the acetylide peak pairs in complexes **1** (A), **2** (B), **3** (C,D), and **4** (E,F), at the excitation frequencies stated above each graph.*

9. Oscillations in the kinetic traces of the NAP carbonyls

The kinetic traces for the NAP ligand modes, particularly the $\nu(CO)_s$ and $\nu(CO)_a$ peak pairs, were imprinted with a coherent oscillation. This was isolated by exponential fitting of the kinetic trace, which removes the signal decay components and leaves the oscillation in the residual data. The oscillating residual was Fourier transformed to obtain its frequency. The $\nu(CO)_s/\nu(CO)_a$ cross-peaks were found to oscillate with a frequency of 43 cm^{-1}, matching the peak-to-peak separation between these modes and confirming the presence of a coherent state in the population time.

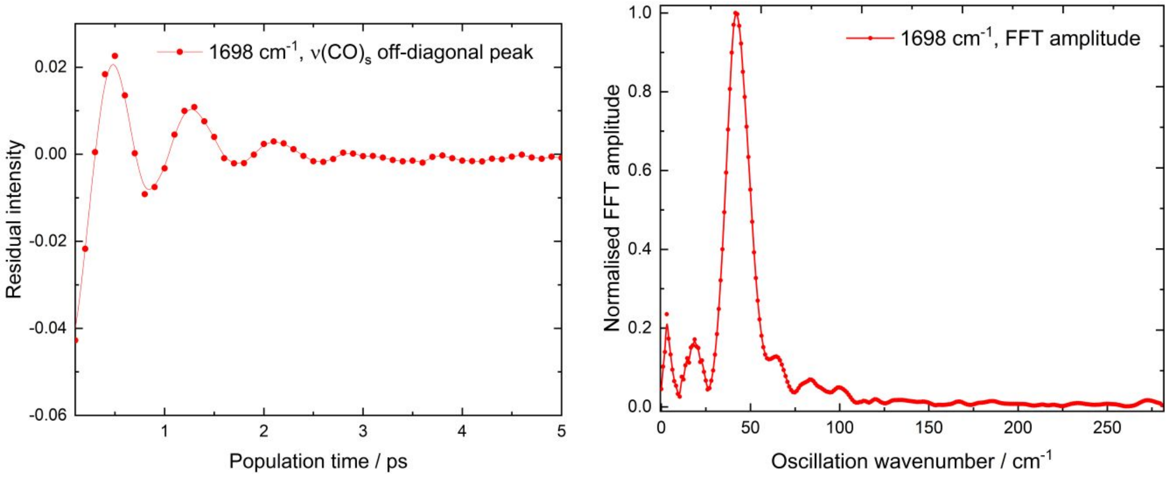

*Figure S26 – Left) Example oscillating residual data obtained after biexponential fitting of the kinetic trace obtained at ω_{Pump} = 1653 cm^{-1}, ω_{Probe} = 1698 cm^{-1}, for complex **3**. Right) Oscillation spectrum obtained by Fourier transformation of the oscillating residual data.*

10. Experimental details for 2D-IR spectroscopy

2D-IR experiments were conducted at the LIFEtime facility of the CLF-STFC Rutherford Appleton Laboratory. The LIFEtime 2D-IR spectrometer has been described in detail elsewhere.[26] Briefly, the setup operates at 100 kHz and comprises two synchronised Yb:KGW regenerative amplifiers (Light Conversion Pharos, 6 W and 15 W) pumping three optical parametric amplifiers (OPAs, Light Conversion Orpheus-one and Orpheus-HP). The 6 W Pharos (180 fs pulse width) pumps two Orpheus-one OPAs to generate two independently tuneable mid-IR probe beams at energies of roughly 0.3 μJ / pulse at ~5 μm wavelength, delivered separately to the sample area via adjustable delay lines and then dispersed spectrally onto two separate 128 element mercury cadmium telluride (MCT) array detectors (Infrared Associates). Digitisation at 100 kHz is achieved using two Infrared Systems 'FPAS units'. Meanwhile, the 15 W Pharos (280 fs pulse width) pumps an Orpheus-HP OPA, generating roughly 1.7 μJ / pulse of mid-IR light at ~5 μm wavelength. This beam passes through a germanium acousto-optic pulse shaper (Phasetech Spectroscopy) and is delivered *via* an adjustable delay line to the sample area. With the pump and probe beams focussed into the sample to ~ 50 μm spot sizes using a 75 mm focal length 90 degree off-axis parabolic mirror and overlapped in space and time, shot-to-shot phase cycling ('four-frame') was used to extract the required third order signals. 2D-IR spectra were acquired by using the pulse shaper to generate two collinear pulses and then recording the third

order signals from the dispersed probe beam as interferograms (22 fs step size out to a final time of 4026 fs) that were then Fourier transformed to give the 2D-IR spectra. The pump OPA was tuned to centre frequencies of either 1680, 2010, or 2080 cm^{-1} and the two probe OPAs were tuned to 1370, 1520, 1680 or 2050 cm^{-1} for the data in the main text. The polarisation of the probe beams and the pump beam were set parallel to the optical bench.

11. References

(1) Frisch, M. J.; Trucks, G. W.; Schlegel, H. B.; Scuseria, G. E.; Robb, M. A.; Cheeseman, J. R.; Scalmani, G.; Barone, V.; Petersson, G. A.; Nakatsuji, H.; Li, X.; Caricato, M.; Marenich, A. V.; Bloino, J.; Janesko, B. G.; Gomperts, R.; Mennucci, B.; Hratchian, H. P.; Ortiz, J. V.; Izmaylov, A. F.; Sonnenberg, J. L.; Williams-Young, D.; Ding, F.; Lipparini, F.; Egidi, F.; Goings, J.; Peng, B.; Petrone, A.; Henderson, T.; Ranasinghe, D.; Zakrzewski, V. G.; J. Gao, N. R.; Zheng, G.; Liang, W.; Hada, M.; Ehara, M.; Toyota, K.; Fukuda, R.; Hasegawa, J.; Ishida, M.; Nakajima, T.; Honda, Y.; Kitao, O.; Nakai, H.; Vreven, T.; Throssell, K.; Montgomery, J. A.; Jr.; Peralta, J. E.; Ogliaro, F.; Bearpark, M. J.; Heyd, J. J.; Brothers, E. N.; Kudin, K. N.; Staroverov, V. N.; Keith, T. A.; Kobayashi, R.; Normand, J.; Raghavachari, K.; Rendell, A. P.; Burant, J. C.; Iyengar, S. S.; Tomasi, J.; Cossi, M.; Millam, J. M.; Klene, M.; Adamo, C.; Cammi, R.; Ochterski, J. W.; Martin, R. L.; Morokuma, K.; Farkas, O.; Foresman, J. B.; Fox, D. J. Gaussian09. Gaussian Inc: Wallingford CT 2016.

(2) Whaley, R. C.; Petitet, A. Software: Practice and Experience. *Softw. Pract. Exp.* **2005**, *35*, 101–121.

(3) Whaley, R. C.; Petitet, A.; Dongarra, J. J. Automated Empirical Optimization of Software and the {ATLAS} Project. *Parallel Comput.* **2001**, *27* (1--2), 3–35.

(4) Pascual-ahuir, J. L.; Silla, E.; Tuñon, I. GEPOL: An Improved Description of Molecular Surfaces. III. A New Algorithm for the Computation of a Solvent-excluding Surface. *J. Comput. Chem.* **1994**, *15* (10), 1127–1138. https://doi.org/10.1002/jcc.540151009.

(5) Miertuš, S.; Scrocco, E.; Tomasi, J. Electrostatic Interaction of a Solute with a Continuum. A Direct Utilizaion of AB Initio Molecular Potentials for the Prevision of Solvent Effects. *Chem. Phys.* **1981**, *55* (1), 117–129. https://doi.org/10.1016/0301-0104(81)85090-2.

(6) Miertuš, S.; Tomasi, J. Approximate Evaluations of the Electrostatic Free Energy and Internal Energy Changes in Solution Processes. *Chem. Phys.* **1982**, *65* (2), 239–245. https://doi.org/10.1016/0301-0104(82)85072-6.

(7) Adamo, C.; Barone, V. Toward Reliable Density Functional Methods without Adjustable Parameters: The PBE0 Model. *J. Chem. Phys.* **1999**, *110* (13), 6158–6170. https://doi.org/10.1063/1.478522.

(8) Weigend, F.; Furche, F.; Ahlrichs, R. Gaussian Basis Sets of Quadruple Zeta Valence Quality for Atoms H-Kr. *J. Chem. Phys.* **2003**, *119* (24), 12753–12762. https://doi.org/10.1063/1.1627293.

(9) Weigend, F.; Ahlrichs, R. Balanced Basis Sets of Split Valence, Triple Zeta Valence and Quadruple Zeta Valence Quality for H to Rn: Design and Assessment of Accuracy. *Phys. Chem. Chem. Phys.* **2005**, *7* (18), 3297–3305. https://doi.org/10.1039/b508541a.

(10) Weigend, F.; Baldes, A. Segmented Contracted Basis Sets for One- and Two-Component Dirac-Fock Effective Core Potentials. *J. Chem. Phys.* **2010**, *133* (17). https://doi.org/10.1063/1.3495681.

(11) Scott, A. P.; Radom, L. Harmonic Vibrational Frequencies: An Evaluation of HartreeFock, Møller-Plesset, Quadratic Configuration Interaction, Density Functional Theory, and Semiempirical Scale Factors. *J. Phys. Chem.* **1996**, *100* (41), 16502–16513.

(12) Kesharwani, M. K.; Brauer, B.; Martin, J. M. L. Frequency and Zero-Point Vibrational Energy Scale Factors for Double-Hybrid Density Functionals (and Other Selected Methods): Can Anharmonic Force Fields Be Avoided? **2014**. https://doi.org/10.1021/jp508422u.

(13) Laury, M. L.; Carlson, M. J.; Wilson, A. K. Vibrational Frequency Scale Factors for Density Functional Theory and the Polarization Consistent Basis Sets. *J. Comput. Chem.* **2012**, *33* (30), 2380–2387. https://doi.org/10.1002/jcc.23073.

(14) Halls, D. M.; Velkovski, J.; Schlegel, H. B. Harmonic Frequency Scaling Factors for Hartree- Fock, S-VWN, B-LYP, B3-LYP, B3-PW91 and MP2 with the Sadlej PVTZ Electric Property Basis Set. *Theor. Chem. Acc.* **2001**, *105*, 413--421. https://doi.org/10.1007/s002140000204.

(15) Irikura, K. K.; Johnson, R. D.; Kacker, R. N. Uncertainties in Scaling Factors for Ab Initio Vibrational

Frequencies. *J. Phys. Chem. A* **2005**, *109* (37), 8430–8437. https://doi.org/10.1021/jp052793n.

(16) Frisch, M. J.; Trucks, G. W.; Schlegel, H. B.; Scuseria, G. E.; Robb, M. A.; Cheeseman, J. R.; Scalmani, G.; Barone, V.; Petersson, G. A.; Nakatsuji, H.; Li, X.; Caricato, M.; Marenich, A. V; Bloino, J.; Janesko, B. G.; Gomperts, R.; Mennucci, B.; Hratchian, H. P.; Ortiz, J. V; Izmaylov, A. F.; Sonnenberg, J. L.; Williams-Young, D.; Ding, F.; Lipparini, F.; Egidi, F.; Goings, J.; Peng, B.; Petrone, A.; Henderson, T.; Ranasinghe, D.; Zakrzewski, V. G.; Gao, J.; Rega, N.; Zheng, G.; Liang, W.; Hada, M.; Ehara, M.; Toyota, K.; Fukuda, R.; Hasegawa, J.; Ishida, M.; Nakajima, T.; Honda, Y.; Kitao, O.; Nakai, H.; Vreven, T.; Throssell, K.; Montgomery, J. A., J.; Peralta, J. E.; Ogliaro, F.; Bearpark, M. J.; Heyd, J. J.; Brothers, E. N.; Kudin, K. N.; Staroverov, V. N.; Keith, T. A.; Kobayashi, R.; Normand, J.; Raghavachari, K.; Rendell, A. P.; Burant, J. C.; Iyengar, S. S.; Tomasi, J.; Cossi, M.; Millam, J. M.; Klene, M.; Adamo, C.; Cammi, R.; Ochterski, J. W.; Martin, R. L.; Morokuma, K.; Farkas, O.; Foresman, J. B.; Fox, D. J. Gaussian 16, Revision A.03. Gaussian, Inc.: Wallingford CT 2016.

(17) Bloino, J. A VPT2 Route to Near-Infrared Spectroscopy: The Role of Mechanical and Electrical Anharmonicity. *J. Phys. Chem. A* **2015**, *119* (21), 5269–5287. https://doi.org/10.1021/jp509985u.

(18) Hamm, P.; Zanni, M. *Concepts and Methods of 2D Infrared Spectroscopy*; Cambridge University Press: Cambridge, 2011. https://doi.org/10.1017/CBO9780511675935.

(19) Robben, K. C.; Cheatum, C. M. Least-Squares Fitting of Multidimensional Spectra to Kubo Line-Shape Models. *J. Phys. Chem. B* **2021**, *125* (46), 12876–12891. https://doi.org/10.1021/acs.jpcb.1c08764.

(20) Kwak, K.; Park, S.; Finkelstein, I. J.; Fayer, M. D. Frequency-Frequency Correlation Functions and Apodization in Two-Dimensional Infrared Vibrational Echo Spectroscopy: A New Approach. *J. Chem. Phys.* **2007**, *127* (12), 124503. https://doi.org/10.1063/1.2772269.

(21) Kwak, K.; Rosenfeld, D. E.; Fayer, M. D. Taking Apart the Two-Dimensional Infrared Vibrational Echo Spectra: More Information and Elimination of Distortions. *J. Chem. Phys.* **2008**, *128* (20), 204505. https://doi.org/10.1063/1.2927906.

(22) Fenn, E. E.; Fayer, M. D. Extracting 2D IR Frequency-Frequency Correlation Functions from Two Component Systems. *J. Chem. Phys.* **2011**, *135* (7). https://doi.org/10.1063/1.3625278.

(23) Guo, Q.; Pagano, P.; Li, Y.-L. L.; Kohen, A.; Cheatum, C. M. Line Shape Analysis of Two-Dimensional Infrared Spectra. *J. Chem. Phys.* **2015**, *142* (21), 212427. https://doi.org/10.1063/1.4918350.

(24) Duan, R.; Mastron, J. N.; Song, Y.; Kubarych, K. J. Direct Comparison of Amplitude and Geometric Measures of Spectral Inhomogeneity Using Phase-Cycled 2D-IR Spectroscopy. *J. Chem. Phys.* **2021**, *154* (17), 174202. https://doi.org/10.1063/5.0043961.

(25) Virtanen, P.; Gommers, R.; Oliphant, T. E.; Haberland, M.; Reddy, T.; Cournapeau, D.; Burovski, E.; Peterson, P.; Weckesser, W.; Bright, J.; van der Walt, S. J.; Brett, M.; Wilson, J.; Millman, K. J.; Mayorov, N.; Nelson, A. R. J.; Jones, E.; Kern, R.; Larson, E.; Carey, C. J.; Polat, \.Ilhan; Feng, Y.; Moore, E. W.; VanderPlas, J.; Laxalde, D.; Perktold, J.; Cimrman, R.; Henriksen, I.; Quintero, E. A.; Harris, C. R.; Archibald, A. M.; Ribeiro, A. H.; Pedregosa, F.; van Mulbregt, P.; SciPy 1.0 Contributors. {SciPy} 1.0: Fundamental Algorithms for Scientific Computing in Python. *Nat. Methods* **2020**, *17*, 261–272. https://doi.org/10.1038/s41592-019-0686-2.

(26) Donaldson, P. M.; Greetham, G. M.; Shaw, D. J.; Parker, A. W.; Towrie, M. A 100 KHz Pulse Shaping 2D-IR Spectrometer Based on Dual Yb:KGW Amplifiers. *J. Phys. Chem. A* **2018**, *122* (3), 780–787. https://doi.org/10.1021/acs.jpca.7b10259.
